# Coralline algal calcification: A morphological and process-based understanding

**DOI:** 10.1371/journal.pone.0221396

**Published:** 2019-09-26

**Authors:** Merinda C. Nash, Guillermo Diaz-Pulido, Adela S. Harvey, Walter Adey

**Affiliations:** 1 Department of Botany, National Museum of Natural History, Smithsonian Institution, Washington DC, United States of America; 2 Research School of Earth Sciences, Australian National University, Canberra, ACT, Australia; 3 Griffith School of Environment and Science, and Australian Rivers Institute, Coast and Estuaries, Nathan Campus, Griffith University, Nathan, Queensland, Australia; 4 Department of Ecology, Environment and Evolution, La Trobe University, Bundoora, Victoria, Australia; Helmholtz-Zentrum fur Ozeanforschung Kiel, GERMANY

## Abstract

**Research purpose and findings:**

Coralline algae are key biological substrates of many carbonate systems globally. Their capacity to build enduring crusts that underpin the formation of tropical reefs, rhodolith beds and other benthic substrate is dependent on the formation of a calcified thallus. However, this important process of skeletal carbonate formation is not well understood. We undertook a study of cellular carbonate features to develop a model for calcification. We describe two types of cell wall calcification; 1) calcified primary cell wall (PCW) in the thin-walled elongate cells such as central medullary cells in articulated corallines and hypothallial cells in crustose coralline algae (CCA), 2) calcified secondary cell wall (SCW) with radial Mg-calcite crystals in thicker-walled rounded cortical cells of articulated corallines and perithallial cells of CCA. The distinctive banding found in many rhodoliths is the regular transition from PCW-only cells to SCW cells. Within the cell walls there can be bands of elevated Mg with Mg content of a few mol% higher than radial Mg-calcite (M-type), ranging up to dolomite composition (D-type).

**Model for calcification:**

We propose the following three-step model for calcification. 1) A thin (< 0.5 μm) PCW forms and is filled with a mineralising fluid of organic compounds and seawater. Nanometer-scale Mg-calcite grains precipitate on the organic structures within the PCW. 2) Crystalline cellulose microfibrils (CMF) are extruded perpendicularly from the cellulose synthase complexes (CSC) in the plasmalemma to form the SCW. 3) The CMF soaks in the mineralising fluid as it extrudes and becomes calcified, retaining the perpendicular form, thus building the radial calcite. In *Clathromorphum*, SCW formation lags PCW creating a zone of weakness resulting in a split in the sub-surface crust. All calcification seems likely to be a bioinduced rather than controlled process. These findings are a substantial step forward in understanding how corallines calcify.

## Introduction

Calcifying red algae are key components of many marine ecosystems globally. One of their main values is substrate provision [1,2] via their formation of calcified structures. The *Peyssonnelia* species mineralise aragonite [3–5], whereas the coralline algae (Corallinales, Sporolithales and Hapalidiales) mineralise Mg-calcite within their cell walls [6–10]. Thick crusts of crustose coralline algae (CCA) bind and cement together coral reefs [9,11,12] and bioherms and biostromes in the tropics [13] and the subarctic [14]. CCA can grow over and bind loose substrate, providing habitat for many other marine organisms in these environments [12,14]. Rhodoliths are key parts of near-shore marine ecosystems globally [15–19]. Fine branching articulated (i.e. geniculate) coralline algae are also key ecosystem components of many shallow, near-shore exposed and tide-pool environments [20–22]. It is calcification in the cell wall of the coralline algae that enables provision of these ecosystem components.

Despite the importance of coralline cell wall calcification in providing these ecosystem services, until recently there has been limited work on coralline algal calcification processes, particularly when compared to the abundance of studies on other calcifiers such as corals, molluscs and foraminifera. At present, there is no comprehensive model of calcification for coralline algae. The overriding motivation for this study is to understand how coralline algae calcify so that this information can be used for both predicting future changes in calcification with climate change and for improving CCA paleo-environmental proxies.

Climate archiving using CCA [14,23–25] has greatly increased interest in calcification mechanisms in the coralline algae. Banding of thick and thin cell walls has been attributed to winter and summer growth [18], although there are conflicting results showing sub-annual banding [26]. Increases in magnesium are attributed to warming temperatures and the thin-walled ‘summer growth’ cells have elevated magnesium. Recent concerns regarding the impacts of rising atmospheric *p*CO_2_ and consequent declining seawater pH (ocean acidification) on carbonate formation has driven contemporary studies on calcification in coralline algae (e.g. [27–33]). The focus has been on the interaction between pH and calcification, the presumption being that calcification is driven by the saturation state of seawater (e.g. [27,34]). However, there are conflicting results indicating that saturation state of the ambient seawater may not be a key driver of calcification [35–37].

Calcification in coralline algae has been viewed as a ‘one box’, or ‘one process’ model (e.g. [37]). A full understanding of calcification is frustrated by the lack of detailed knowledge on how the Mg-calcite is formed, the controls on calcification, and whether the formation process is the same for all types of skeletal carbonate present within coralline algae. There is recognition of cell wall and interfilament carbonate as being morphologically different. The cell wall forms Mg-calcite orientated radial perpendicular to the cell wall (termed radial calcite from now on) whereas the interfilament may have shorter grains orientated parallel to the nearest flat surface [38,39] or as clumps forming a deltoid shape in the *Clathromorphum* species [14]. However, there has been no attempt to separate the role of seawater carbonate concentration in the formation of these differing skeletal parts. While recent work has used boron isotopes (δ^11^B) as a proxy for pH at the site of calcification in CCA and articulated coralline algae [40], studying the isotopic composition of individual anatomical components separately is frustrated by the sub-micron scale of the anatomical components and associated skeletal features. Even utilizing state-of-the-art techniques in laser ablation Inductively coupled plasma mass spectrometry (ICPMS) this level of organization cannot be examined for δ^11^B pH proxies. Thus it is not possible to identify if there is a difference in chemical signatures between the cell wall and interfilament.

The role of photosynthesis in influencing calcification is also unclear. Fundamentally photosynthesis is a controller of calcification via the provision of substrate [41]. It is also proposed to have an active role by locally elevating internal pH, leading to mineral precipitation in calcifying algae [42–44]. However, there is evidence that photosynthesis is not directly required for calcification to proceed as experimental work has demonstrated a decoupling between photosynthesis and calcification [37,45]. Furthermore, there are calcified non-photosynthetic parasitic CCA [38,46,47] and CCA continue to grow in Subarctic/Arctic winter darkness [48,49].

In addition to the uncertainties around the influence of seawater saturation state, internal pH and photosynthesis on calcification, there are conflicting propositions on how much control coralline algae exert over calcification processes and how calcification proceeds, specifically whether calcification is controlled, or induced as a result of a physiological process undertaken for a purpose other than mineral formation. Calcification is presumed induced in green algae [42,43] however there are suggestions of controlled calcification in coralline algae [6].

As detailed studies on sub-micron scale coralline algal biomineral characteristics that could inform understanding of calcification processes are lacking, much of the discussion on coralline biomineralisation has looked to the abundant literature on corals (e.g. [50–53]), molluscs (e.g. [54] and foraminifera (e.g. [55]). However, these calcifiers are all marine animals whereas, the coralline algae are marine “plants” [56,57]. Thus, relying on interpretations of skeletal formation within marine animals may confuse interpretation of calcification processes in the coralline cell walls.

To understand the potential impacts of climate change on coralline algal calcification, it is necessary to accurately identify the differing calcified components within the coralline crust and cell walls, and then determine whether the differing components have different processes of calcification [40]. Until this has been achieved, an accurate understanding of climate change impacts on calcification will be frustrated. Building that understanding is the motivation for this study.

### Aim of this study

In this study, we set out to combine existing knowledge of plant cell wall formation with high-magnification observations of cell wall features in coralline algae to develop a testable model of calcification. Specifically; we identify the multiple types of calcification and organic features within the coralline algae cell walls and interfilament regions and consider how each of these may form, given our present understanding of plant cell wall formation, organic composition, mineralisation processes and carbon and oxygen isotopic exchange. To identify the cell wall and calcification components we started with a detailed investigation using scanning electron microscopy (SEM-EDS) to identify the common calcification features of CCA (non-geniculate) and articulated (geniculate) corallines. From this, we build a calcification component schematic and then propose a biomineralisation model. We then adapt the model to account for unique features of the *Clathromorphum* species, an important coralline algal genus dominant in the Subarctic region. *Peyssonnelia* species were not studied and it is not known if aspects if these models could be applied to *Peyssonnelia* species calcification.

### Paper structure

As many SEM figures are required to support the development of the model, and in order to facilitate streamlined reading, the paper structure is as follows; background information, proposed calcification model components followed by relevant SEM figures for each model component, discussion, concluding summary and methods. The supporting analytical results and sample detail tables (S1–S3 Tables) are in the supplementary information.

## Background information

### Ultrastructure features of coralline algae and current understanding of calcification

#### Internal anatomy of corallines

Numerous terms have been widely used to describe coralline internal anatomy. For the purposes of this paper, in CCA the thallus is composed of the hypothallus (those parts of the thallus orientated more-or-less parallel to the substrate) and the perithallus (those parts of the thallus orientated more-or-less perpendicular to the surface). The epithallus is the surface layer/s of the crust. All CCA have an intercalary meristem (cambium equivalent) of specific length (depending upon genus); this meristem produces upwards the overlying epithallus and underlying perithallus [58–60]. Cell elongation typically occurs gradually from the meristem for 3–10 cells down before reaching mature size except for *Clathromorphum* that complete all cellular growth in the meristem. Well-defined lateral channels form below the zone of cell elongation. These channels may be present as cell fusions—large holes of ill-defined shape, or smaller rounded holes (secondary pit connections) in the cell walls of adjacent cells of different filaments. The interfilament area (middle lamella in plants) is calcified and ranges in thickness from <0.5 to 2 microns [14,38,39]. The perithallial cells have thick (1–2 microns) cell walls. The base of the crust has a hypothallus of varying thickness, depending upon species, which typically forms the initial layer of crust laid down from the settled spore [58,60,61]. The hypothallial cells are elongate and thin walled [38]. These thin hypothallial-style cells also form from the perithallial meristem where the living crust has been wounded and rapid growth is required to cover the damaged area, and within emptied conceptacles where cells have regrown [14]. Some rhodolith-forming genera, e.g. *Lithothamnion*, can form internal bandings of alternating thin-walled elongate cells, high in Mg, to shorter, thicker walled cells, lower in Mg content [18,26].

Articulate coralline branches differ from CCA as they have a central medulla (orientated more less- parallel to the thallus surface) and peripheral cortex (orientated more-or-less perpendicular to the thallus surface). The medullary cells are elongated and thin-walled (< 500 nm) and there may be a layers of shorter more rounded cortical cells between the medullary and the surficial epithallus [62,63].

That actual mechanism of mineral formation is not definitively known. Historical studies of calcification in coralline algae have alternatively proposed that radial calcite is formed by 1) insertion of calcite crystals between radially-arranged polysaccharide fibrils [6] or 2) as mineral formation within an organic matrix [64]. The proposal by Cabioch and Giraud [6] would require the calcite crystal to be formed within the cell and pushed into the cell wall, perhaps similarly to coccolith plates. Borowitzka [43] proposed that coralline algae have semi-organised calcification, suggesting they may control the mineral formation. However, more recent experimental work has suggested that at least for the articulate coralline *Amphiroa*, the organism exerts little control over the mineral formation process [65].

### Cell walls in higher plants and fleshy macroalgae

**Fig 1:**
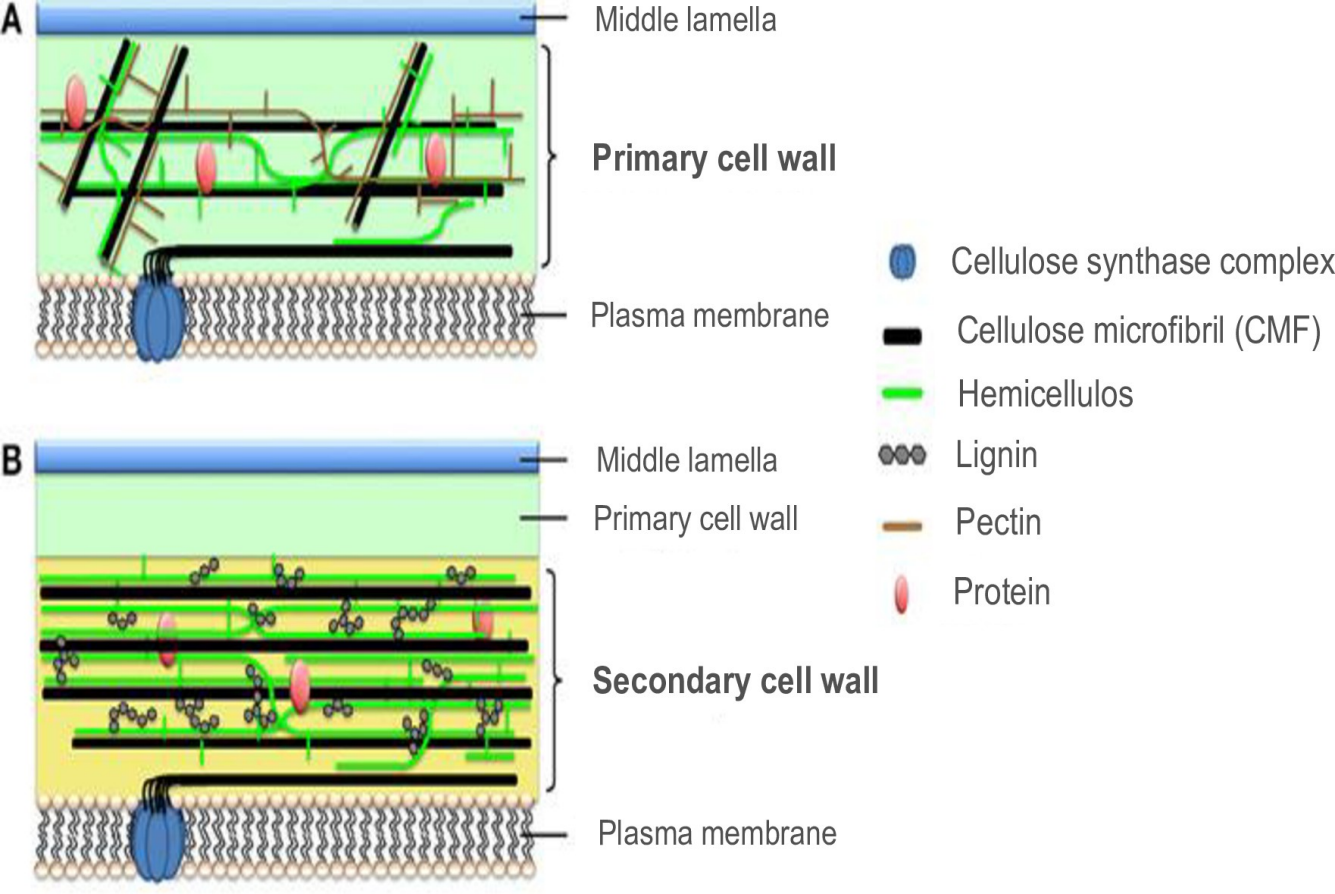
**Cellulose formation in primary (A) and secondary plant cell walls (B).** Figure republished from [67] under a CC BY license, with permission from Frontiers, Copyright 2015.

Plant cells are enclosed by a lipid plasma membrane (plasmalemma) (Fig 1) [66,67] and the cell wall (primary cell wall). Between the cells is the middle lamella, which is pectin-rich and acts to glue adjoining cells together. Mature cells have additionally a secondary cell wall whereas newly growing or wound-repair cells generally only have primary cell walls. The thinner primary cell walls allow extension and shape flexibility [68]. Cellulose forms the bulk of the cell wall in plants [66,69] and algae [70]. Cellulose is a crystalline polysaccharide with molecules of linear chains of (1,4) β-linked-d-glucan (repeating monomers of glucose attached end to end) [66]. Crystalline cellulose microfibrils (CMF) are formed by Cellulose Synthase Complexes (CSC) within the plasmalemma (Fig 1) [67,69,71]. The CSC is a protein complex that can move within the membrane [72]. In plants, the CSC group in sixes in a rosette configuration. Each CSC polymerizes a single β-1,4-glucan cellulose chain that initially extrudes perpendicular to the membrane, then folds over, clumping with the other five individual chains to form the CMF. In the secondary cell wall of plants, the process of cellulose formation is particularly important as the concentrically-arranged lamellae of cellulose provides the structural strength enabling vertical growth and development of thick woody stems [73,74]. Primary and secondary cell walls have hemicellulose microfibrils, a matrix polysaccharide, also extruded from the plasma membrane. The secondary cell wall in plants has lignin present that further adds to the strength of the secondary cell wall. Both green and red algae can have primary and secondary cell walls [70], similarly to higher plants. The decalcified joints of the articulated coralline algae *Calliarthron cheilosporioides* have lignin and secondary cell walls [75]. Green algae have both rosette CSC, and CSC in linear patterns referred to as terminal complexes whereas red fleshy (non-calcified) algae are only recorded as having the linear pattern CSC [70]. There is no published work, as far as we are aware, on the cellulose formation mechanisms in calcified red algae.

The plasmalemma serves as a general permeability barrier between the cell vacuole and the cell wall because most water-soluble (polar) molecules cannot cross the lipid bi-layer [76]. The plasma cell membranes are themselves organelles and have a wide variety of molecular pumps and channels embedded in the membrane. Small, uncharged molecules can move through the membrane without the pumps. However, larger charged molecules require active ion transport to cross the membrane into the cell vacuole. This requirement for active transport means the plasmalemma is effectively an impermeable membrane. The outer perimeter of the cell wall is a permeable organic mesh [77] and active ion transport is not required.

#### Clues for calcification drivers- differences in organic compounds between fleshy red algae and coralline algae

The internal physical structure of coralline algae is similar to most fleshy red algae in being based in compact arrays of filaments (cells of common ancestry connected by primary pits, but differs in having an intercalary meristem) [1,6,78]. The intriguing possibility has been raised that calcification may be a process that would proceed commonly in fleshy algae, if specific calcification inhibitors were absent [43]. Considering the similarity in internal anatomical structure, we can look to the organic compounds that are present in calcifying algae but absent in fleshy algae (or the inverse), for potential clues as to drivers (or inhibitors) of calcification.

Analyses of organic compounds from 24 corallines, both articulated and crustose, found the key differences to fleshy red algae were the presence of a nonconventional sulfated xylogalactan polysaccharide, which has high quantities of xylose [79–82] and the absence of 3,6-anhydrogalactose, which is always present in fleshy red algae [79]. Alginic acids have been implicated in calcification in corallines [81,83] because these polymers have a high affinity for bivalent cations, especially calcium [79,84]. However, Bilan and Usov [79] raise a note of caution that the alginates may be from endolithic organisms within the corallines and not directly involved in calcification. A pectin-like substance has been identified in cell ends of articulated coralline algae [62]. Collagen and chitin have been identified in *Clathromorphum compactum* [85], but it is unknown if these are common to all corallines. The literature is summarised in Table 1.

**Table 1:** Summary of organic compounds known to be present or absent in coralline algae in contrast to fleshy red algae.

Compound	Notes	Reference
Sulfated xylogalactan	Found in all corallines so far	Bilan and Usov 2001 [79] Martone et al., 2010 [80] Navarro et al., 2011 [81] Malagoli et al., 2014 [82]
Alginic acid	Found in all corallines so far. Concentrated in cell walls of *Lithothamnion heterocladum*, appears not present in interfilament	Bilan and Usov 2001 [79] Okasaki et al., 2009 [83] Navarro et al., 2011 [81]
**Absent** 3,6-anyhdrosgalactose	**Not found** in any corallines, always present in fleshy red algae	Bilan and Usov 2001 [79]
2,3-dimethylated galactose in xylogalacton	More in the crustose *L*. *heterocladum* than reported for articulates	Navarro et al., 2011 [81]
Collagen and chitin	Found in *Clathromorphum*	Rahman and Halfar, 2014 [85]

Coralline algae also have the ability to decalcify parts of their skeleton in the formation of cell fusions, reproductive conceptacles [14] and articulated joints (genicula) in the articulated corallines, e.g. *Calliarthron cheilosporioides* [80]. While decalcification and consequent anatomical features are an important part of coralline physiology, and need to be considered in a final analysis of calcification in these organisms, the process of decalcification or formation of cell fusion, or pit connections, will not be considered further in this study.

#### Mineralisation of cellulose: Insights from bone regeneration studies

While there has been little work done on the role of organic substrates in the biomineralisation processes of coralline algae, in the medical field of bone regeneration, the capacity of seaweed-derived polysaccharides such as alginates, to induce mineralisation has been widely studied and applied (e.g. [86–88]). Indeed, naturally derived biopolymers have been making a contribution to the field of regenerative medicine for decades [89,90]. Natural polymers such as cellulose, alginates, hemicellulose, lignin and chitin are turned into hydrogels that are then used as tissue regeneration substrates. Furthermore, advantage can be taken of the cellulose structure to use as a scaffold for bone implants. As an example, processed cellulose soaked in a calcium-rich solution then placed in simulated body fluids formed a mineralised coating (calcium phosphate) within days (e.g. [86,91]) and it has been proposed that regenerated cellulose viscous sponges can be used as implantable bone tissue (e.g. [92,93]). These medical studies provide insight into the potential processes of mineralisation that we can look to in developing a model for coralline calcification.

### Features of plant and bone studies relevant to developing the calcification model

From the published plant and bone studies and cellular features identified in our extensive SEM imaging, there are several key components that we used to inform our calcification model.

1. Firstly, the presence of a secondary cell wall in higher plants and many fleshy red and green algae, indicates that consideration of calcification must include an assessment of secondary cell wall formation in coralline cells. Our assessment (based on SEM images) indicated that the elongated thin-walled cells that form the central medullary cells of articulated corallines and the CCA hypothallial cells form only a primary calcified cell wall. In contrast, the articulated coralline cortical cells and CCA perithallial cells form a secondary cell wall. That leads us to the second key consideration.
2. The main structural difference between the primary and secondary cell walls in plants, is the thicker SCW that is due to the formation of CMF. We look to the cellulose formation as the first step in identifying the process of the SCW radial calcification and consider calcification of the cellulose substrate.
3. Understanding how calcification of cellulose could proceed is informed by the bone regeneration studies. Key to those studies is the use of the cellulose as the scaffold and a calcium-rich fluid mixed with the organism’s body fluids to induce mineral formation without biological activity.
4. Finally, we incorporate the information on organic compounds, present and absent (Table 1), to propose the organism ‘body fluid’ that may be the key to inducing or enabling calcification of, or on, the organic components with the primary and secondary cell walls.

## Calcification model

**Fig 2:**
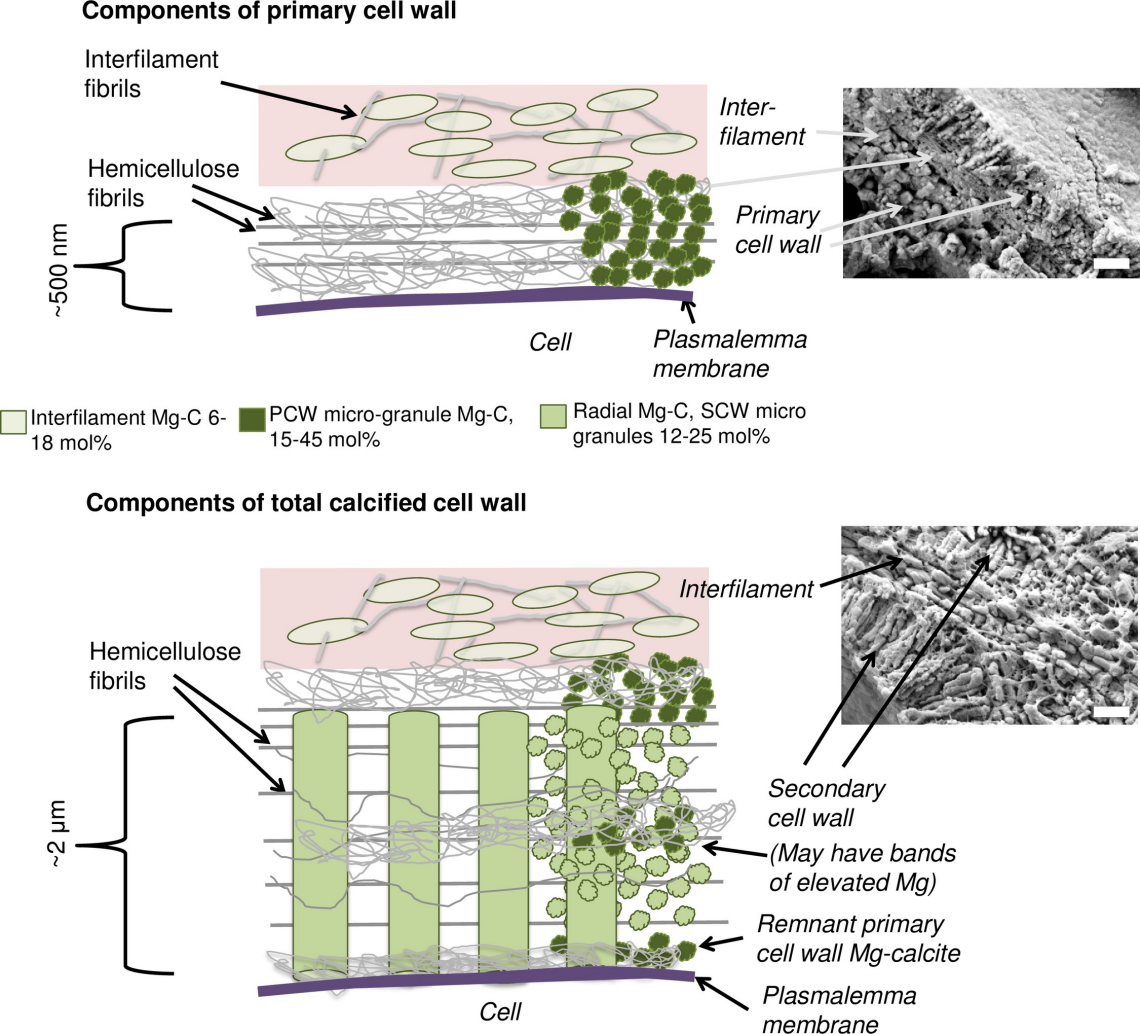
Cell wall components. Not drawn to scale. The pink shading represents the interfilament area. SEM images; top- example of hypothallial cell wall with PCW-only calcification, *Phymatolithon laevigatum*; bottom, example of perithallial cell wall with SCW calcification, *Kvaleya epilaeve*, scale bars- 500 nm. SEM images insets enlarged in 14B (top SEM) and 24B (bottom SEM).

**Fig 3:**
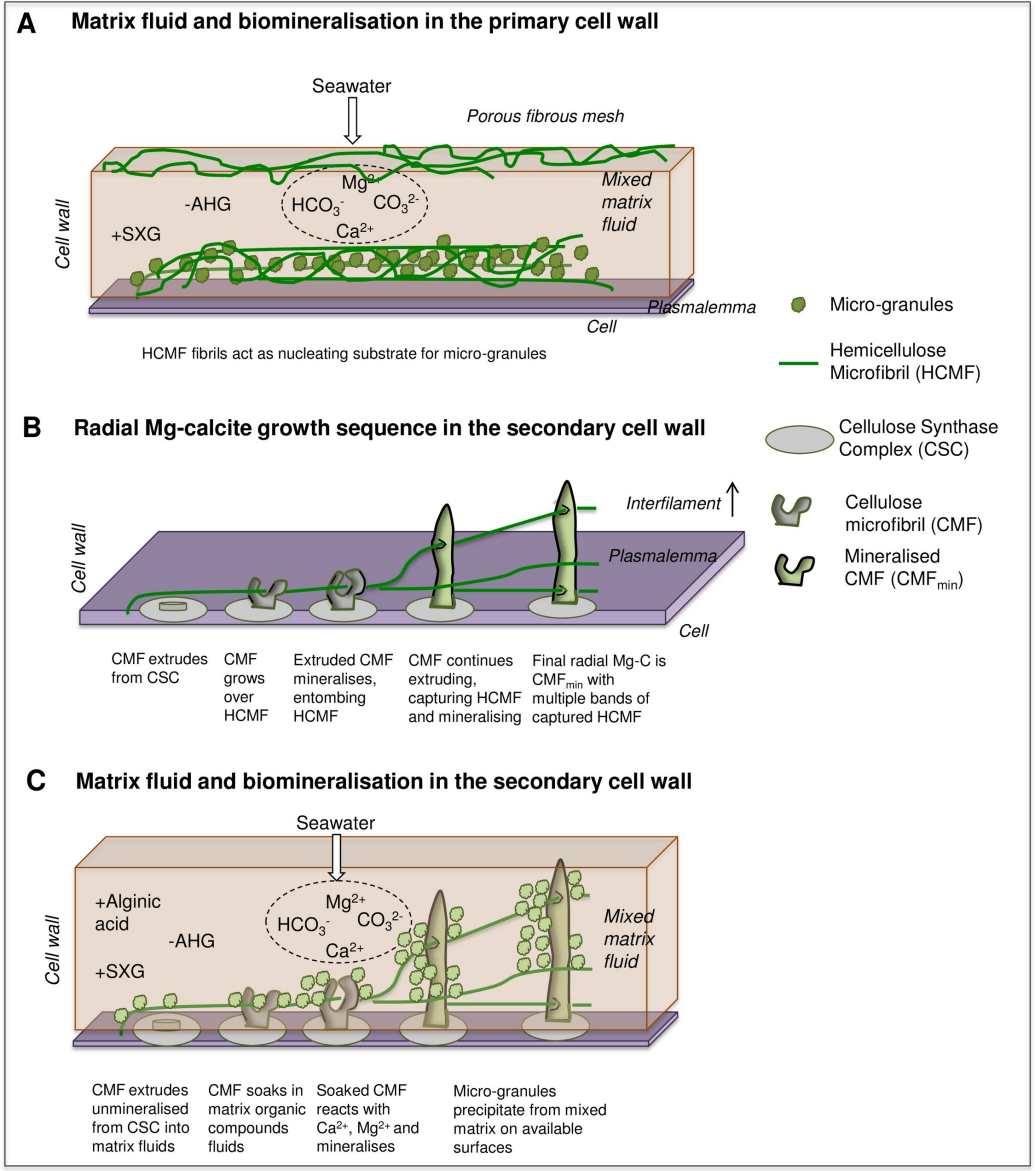
Proposed interaction between organic substrate, organic fluids and seawater for biomineralisation. AHG: 3,6-anyhdrosgalactose. SXG: sulfated xylogalactan.

**Fig 4:**
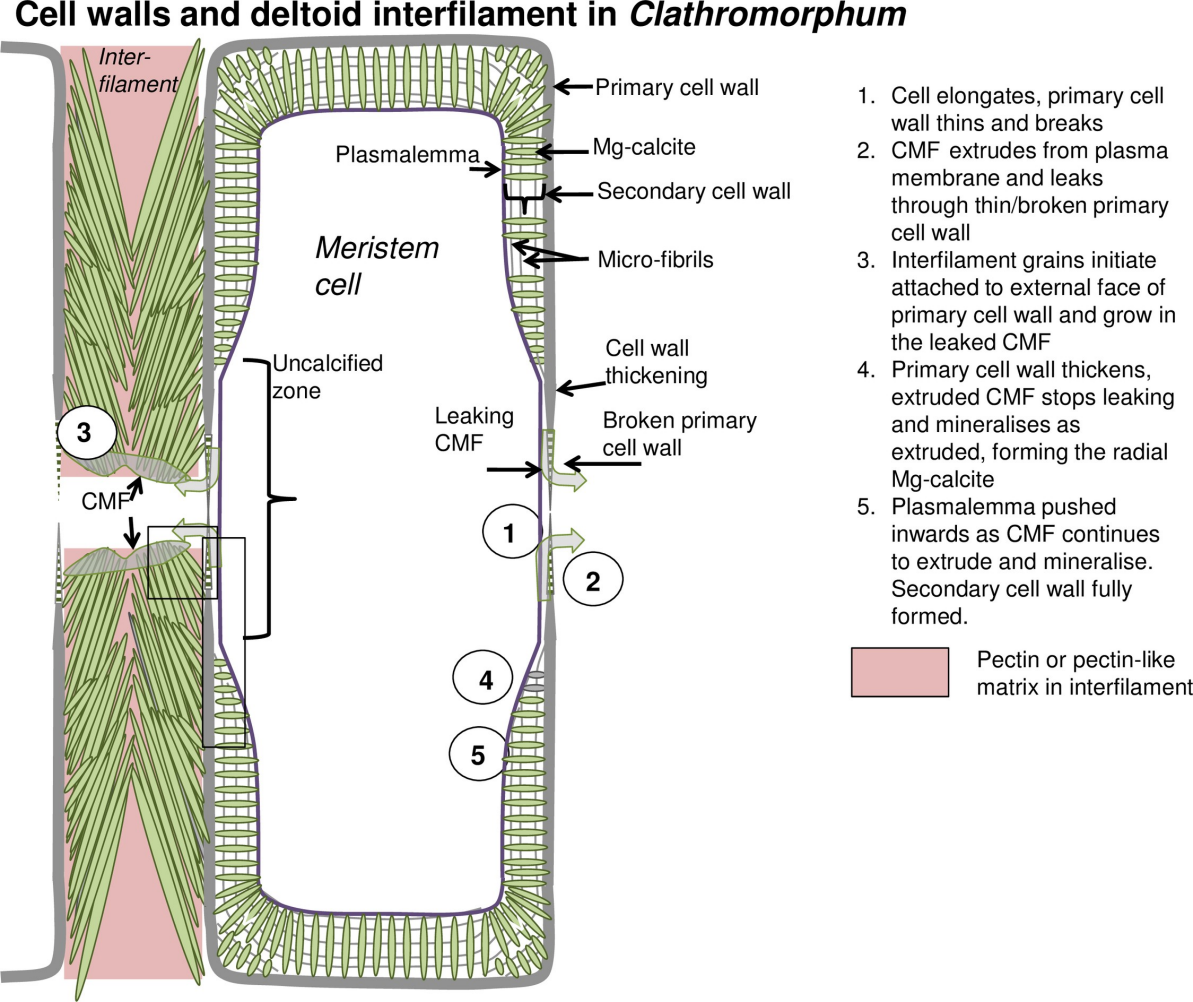
Cell development at meristem split for *Clathromorphum nereostratum*, *C*. *circumscriptum* and *C*. *compactum*. Black boxes enlarged (Fig 5).

**Fig 5:**
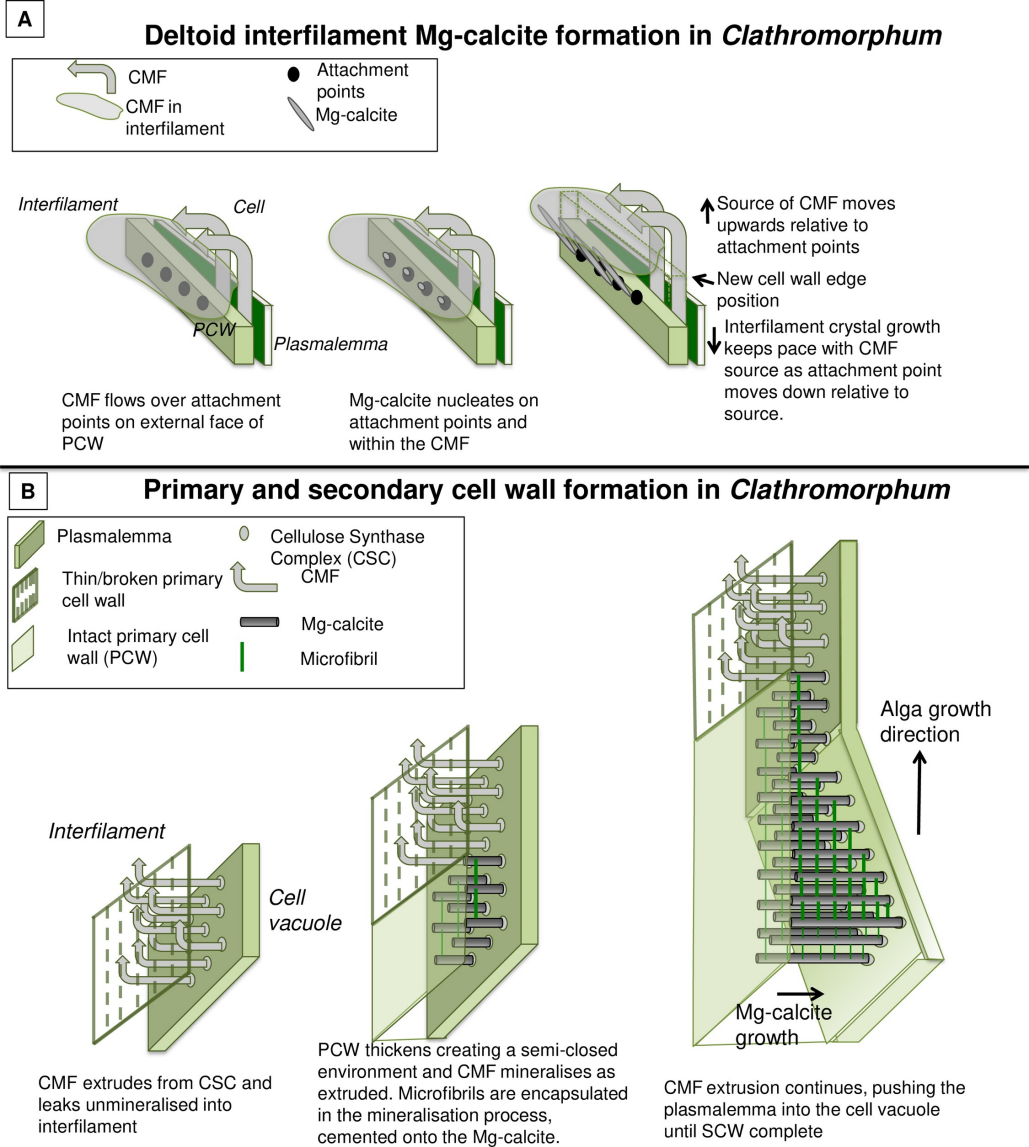
Clathromorphum deltoid interfilament and development of primary (PCW) and secondary cell wall (SCW). (A) Deltoid interfilament calcification in *Clathromorphum*. Interfilament crystals grow until touching crystals growing out from opposite cell walls as in Fig 4. Attachment points- it is not known what these are, but they must be consistently present. Possibly rough spots on the external wall surface, or PCW compounds that attract the CMF. (B) Development of primary and secondary cell wall in *Clathromorphum* (deltoid species).

The following paragraphs are a summary of the components and proposed processes of calcification in coralline algae. The model (Figs 2–5) is drawn from the SEM imaging and Mg-content analyses of a range of species (S1–S3 Tables). The model does not include surficial epithallial cells. The coralline algae organic composition was not analysed for this study and we rely upon information in published literature to develop role of matrix organics. In the remainder of the paper, reference to plants includes both higher plants and fleshy algae. Genera studied (S3 Table) included **CCA**: *Lithothamnion*, *Lithophyllum*, *Porolithon*, *Hydrolithon*, *Sporolithon*, *Spongites*, *Neogoniolithon*, *Kvaleya*, *Leptophytum*, *Phymatolithon*, *Clathromorphum*, *Titanoderma*, *Mesophyllum*, *Pneophyllum*, *Mastophora* and unidentified epiphytes. **Articulated coralline algae:** *Amphiroa*, *Jania*, *Corallina*, *Metagoniolithon*, *Lithothrix* and *Mesophyllum* (species = 37, total number of samples n = 96).

### Carbonate component model

#### Cell walls

**Fig 6:**
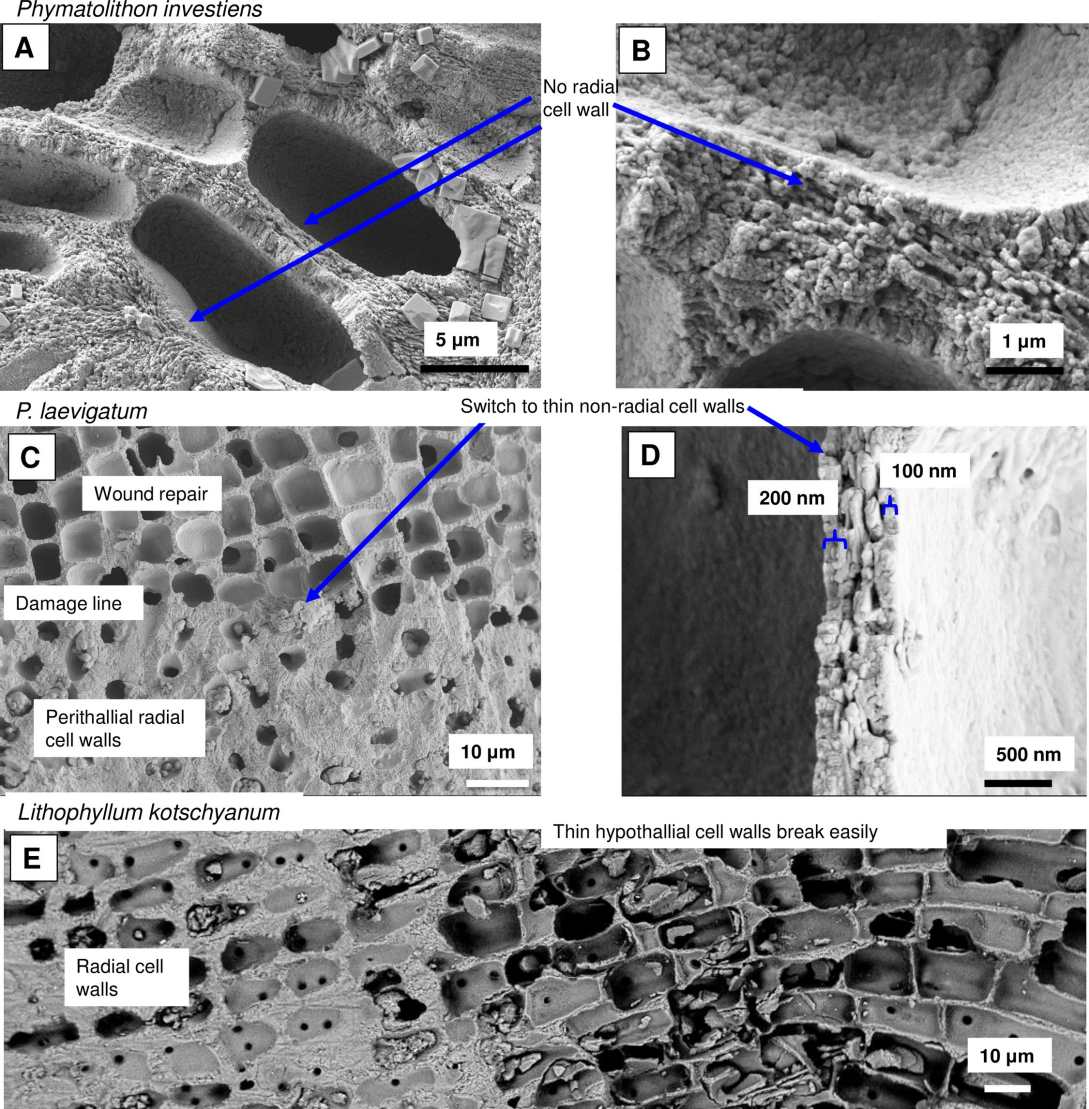
Hypothallial cell walls. (A, B) *Phymatolithon investiens*, (North Norway). The cell wall calcification is inconsistent. Adjacent cell walls with and without substantial calcification. (C, D) *P*. *laevigatum*, (Newfoundland). Cell wall switches from thick SCW to thin PCW for wound repair. (E) *Lithophyllum* kotschyanum (Ryukyu Islands, Japan). Thin cell walls of elongate PCW cells break easily. Elongate cells range in shape from straight rectangular to curved rectangular. A, B, C, D reproduced from [39] under a CC BY license, with permission from John Wiley and sons, Copyright 2017.

**Fig 7:**
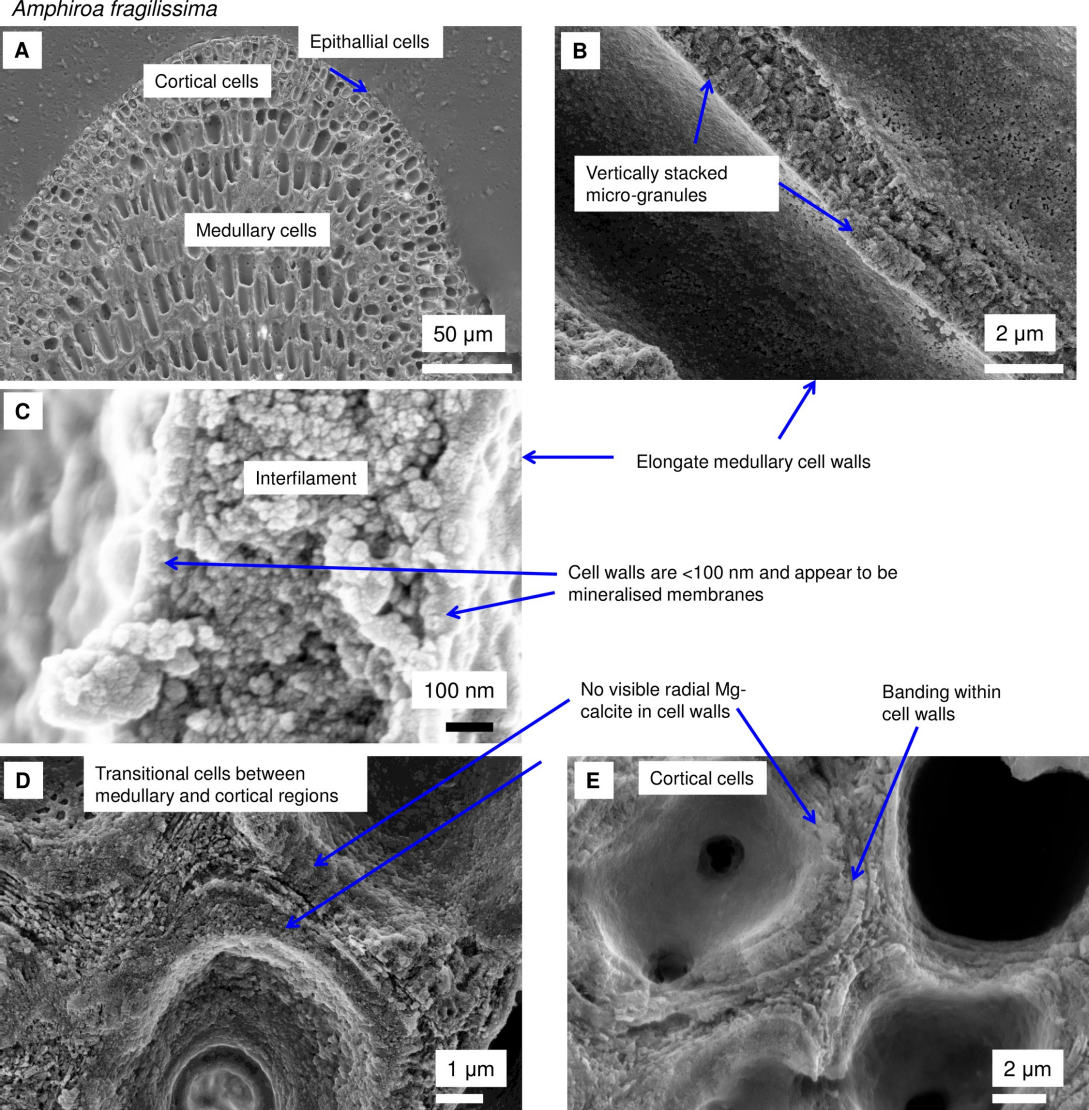
Cell walls in *Amphiroa fragilissima*, (Gold Coast, Queensland, Australia). (A) Overview. (B, C) Medullary cell walls are similar to hypothallial cell walls in CCA having a range of calcification types from a thin mineral coating on a membrane to vertically stacked micro-granules. (D, E) Rounded cortical cells do not have visible radial calcite. There is visible banding within the cell wall.

**Fig 8:**
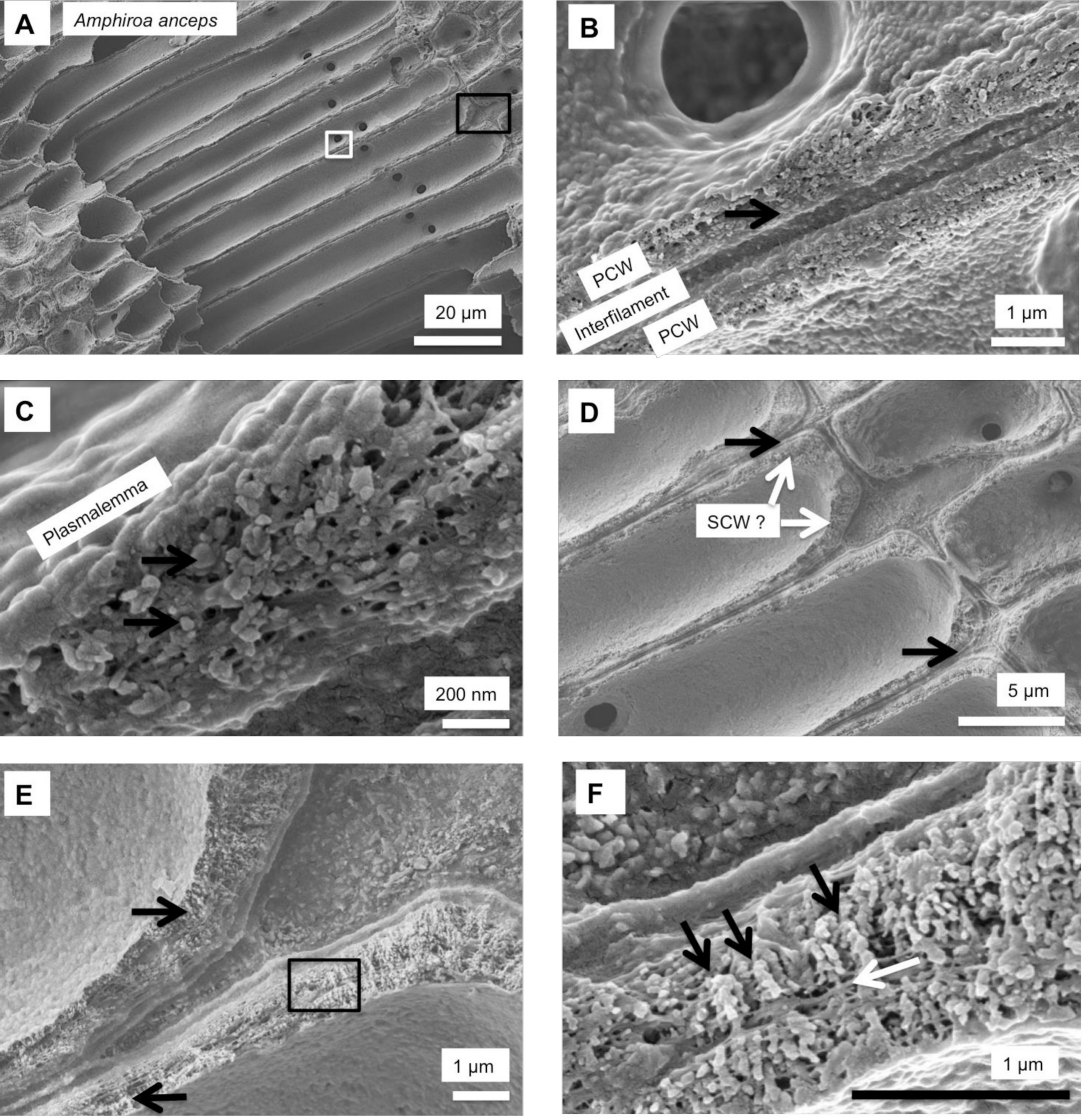
Primary cell wall of medullary cells *Amphiroa anceps*, (Warrnambool, Victoria, Australia). Etched for 35 minutes. (A) Overview of medullary cells. White box enlarged in B, C. Black box enlarged in D, E and F. (B) Etching has removed interfilament grains exposing organic material between cells. (C) PCW wall has irregular shaped grains encapsulated in organic fibrils (black arrows). Fibrils concentrated at external edge of cell wall forming continuous mesh. (D) Concentration of organic fibrils at cell wall edge visible rimming the cell wall (black arrows). The cell walls are thickest at the corners and there may be secondary cell wall formation (white arrows). (E) Vertical alignment of Mg-calcite grains in cell corners (black arrows). Black box enlarged in F. (F) Grains perpendicular to cell wall, possible SCW (black arrows). Laminar fibrils through cell wall (white arrow).

**Fig 9:**
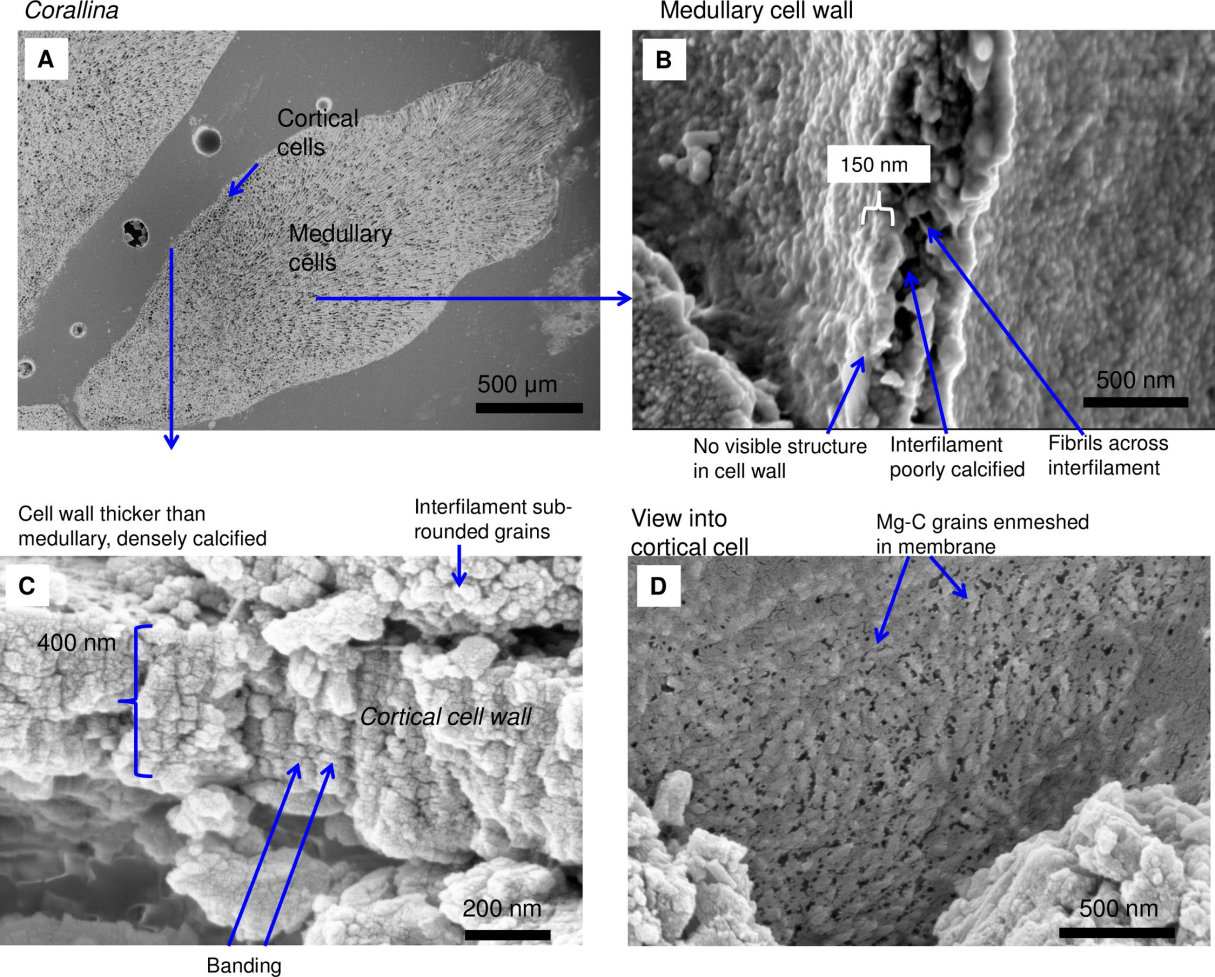
*Corallina* sp., (Coffs Harbour, New South Wales, Australia). (A) Cortical cells are only present along the long edges and not at the apical tip (growth tip). (B) In contrast to the *Amphiroa*, the medullary cell walls are very poorly calcified. (C, D) Cortical cell walls are thicker and more densely calcified than the medullary cells.

**Fig 10:**
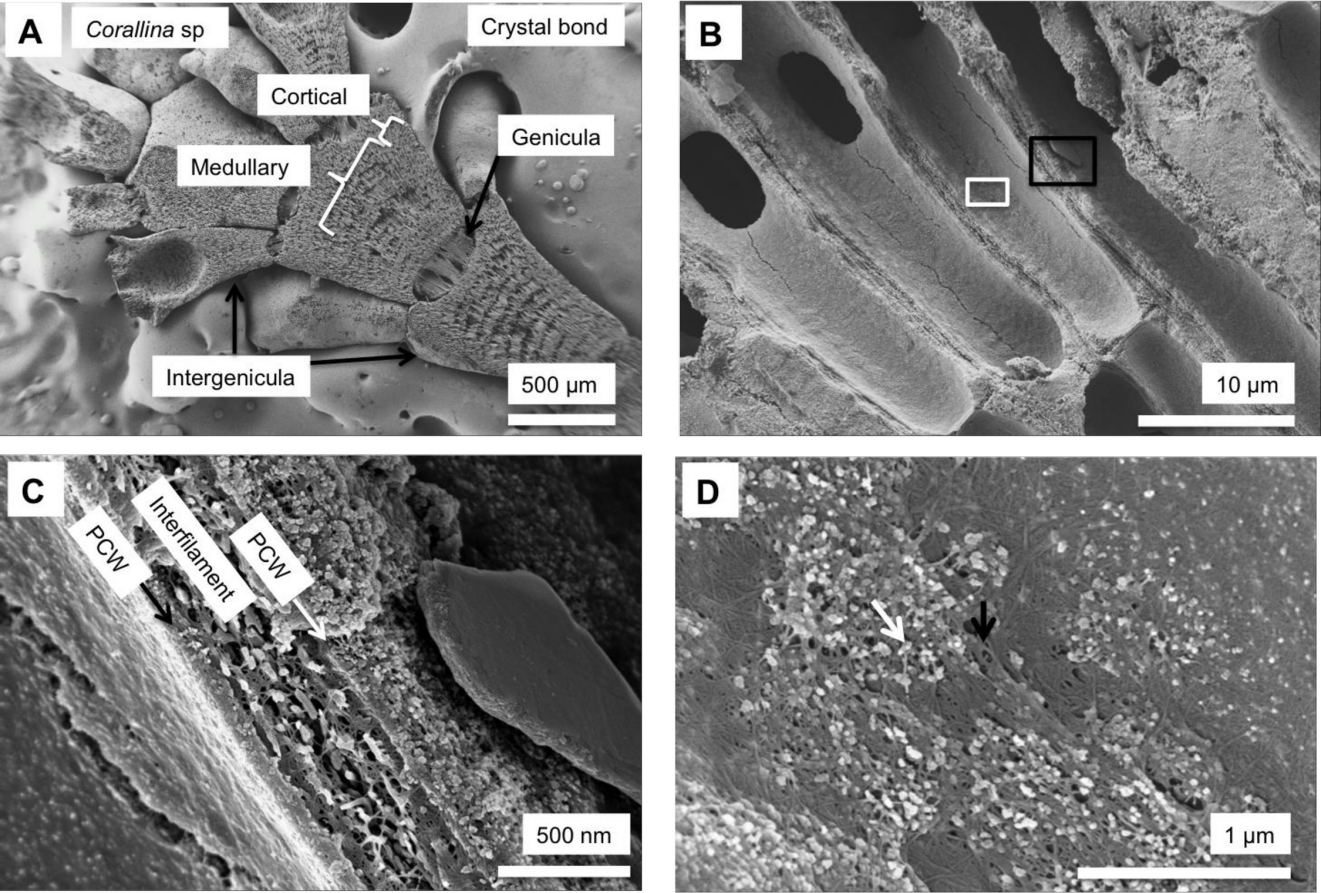
Medullary cells in *Corallina* sp., (Coffs Harbour, New South Wales, Australia). Sample etched for 35 minutes. (A) Overview. (B) Medullary PCW cell walls are thin and flexible. Black box enlarged in C. White box enlarged in D. (C) Cell walls are less than 100 nm wide. The majority of the carbonate is interfilament Mg-calcite. Interfilament grains are elongate and have fibrils attached. (D) View of cell wall from within the cell. Carbonate granules (white arrow) are encapsulated within a mesh of organic fibrils (black arrow).

**Fig 11:**
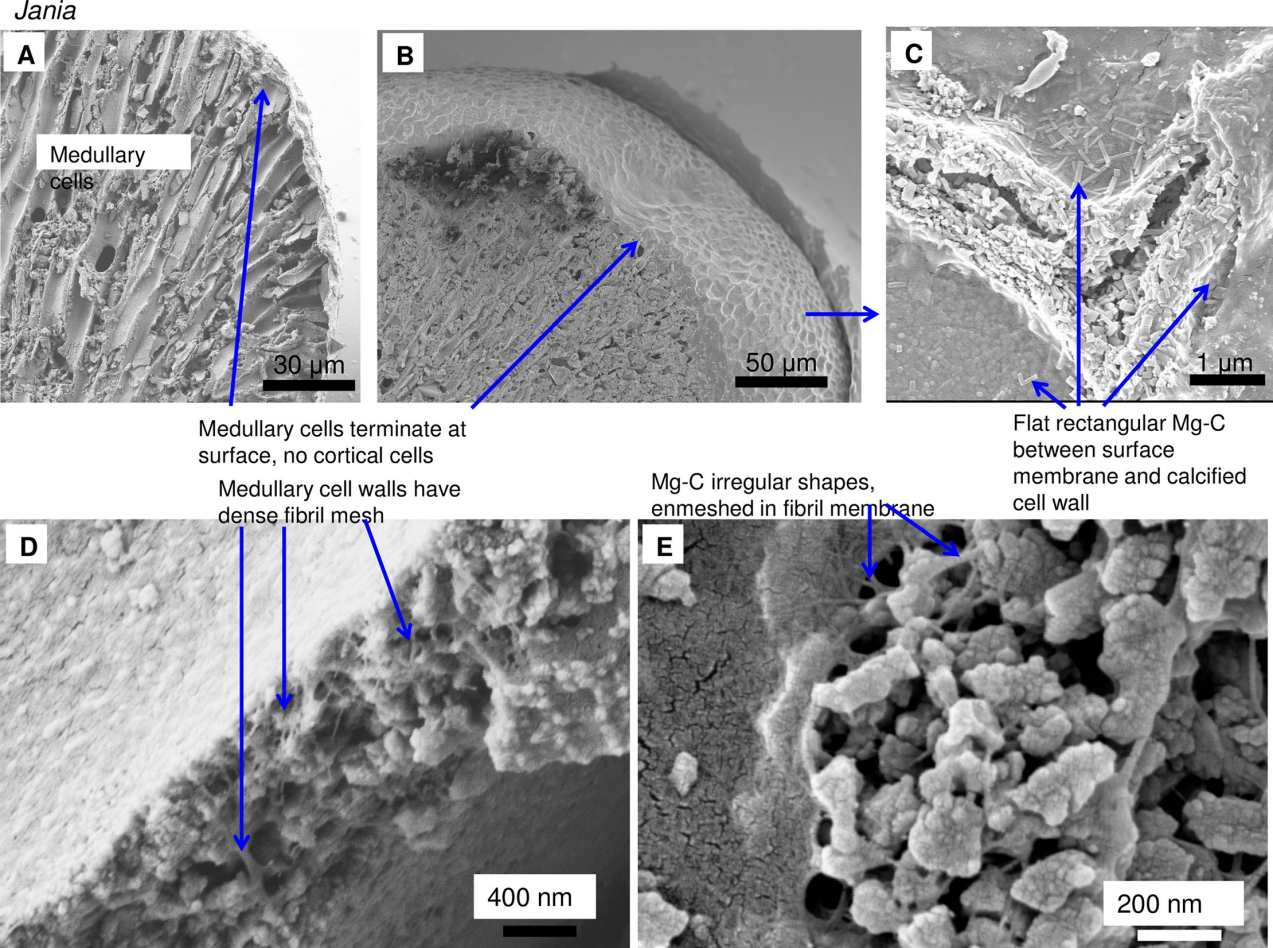
*Jania rosea*, (Tathra, New South Wales, Australia). (A, B) Medullary cells extend to the apical tip and there is no cortical cell layer. (C) Flat rectangular Mg-calcite grains form within the surface membrane. (D, E) Medullary cell walls have dense fibrillar mesh and irregular Mg-calcite grains are enmeshed within this.

**Fig 12:**
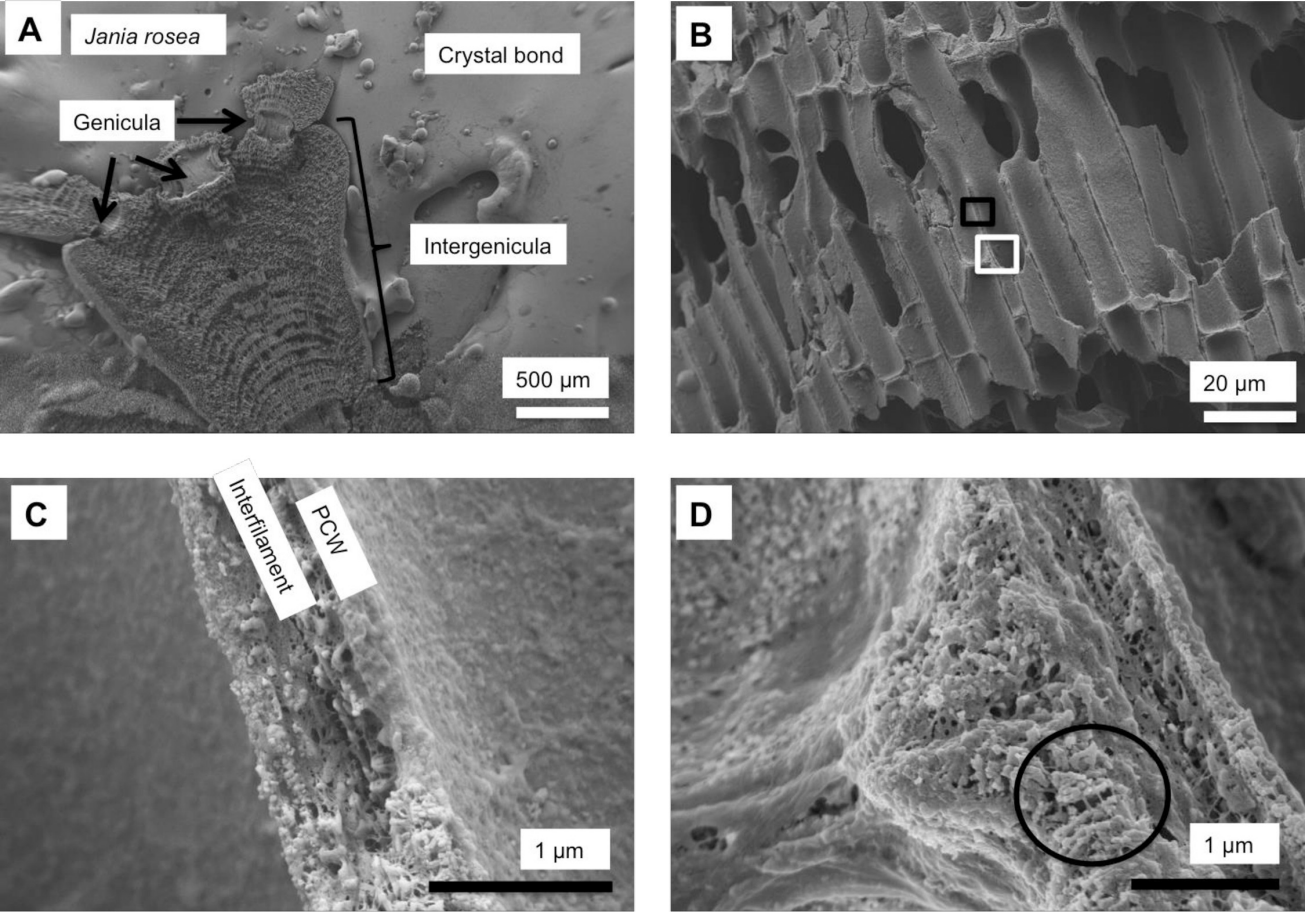
Medullary cells in *Jania rosea*, (Tathra, New South Wales, Australia). Etched for 35 minutes. (A) Overview. (B) Medullary PCW cells have large, broken edged cell fusions. Cell walls are thin. Black box enlarged in C, White box enlarged in D. (C) Cell walls are less than 200 nm wide. Interfilament has been removed by etching. (D) Possible development of radial Mg-calcite (in black circle) in cell wall corners.

**Fig 13:**
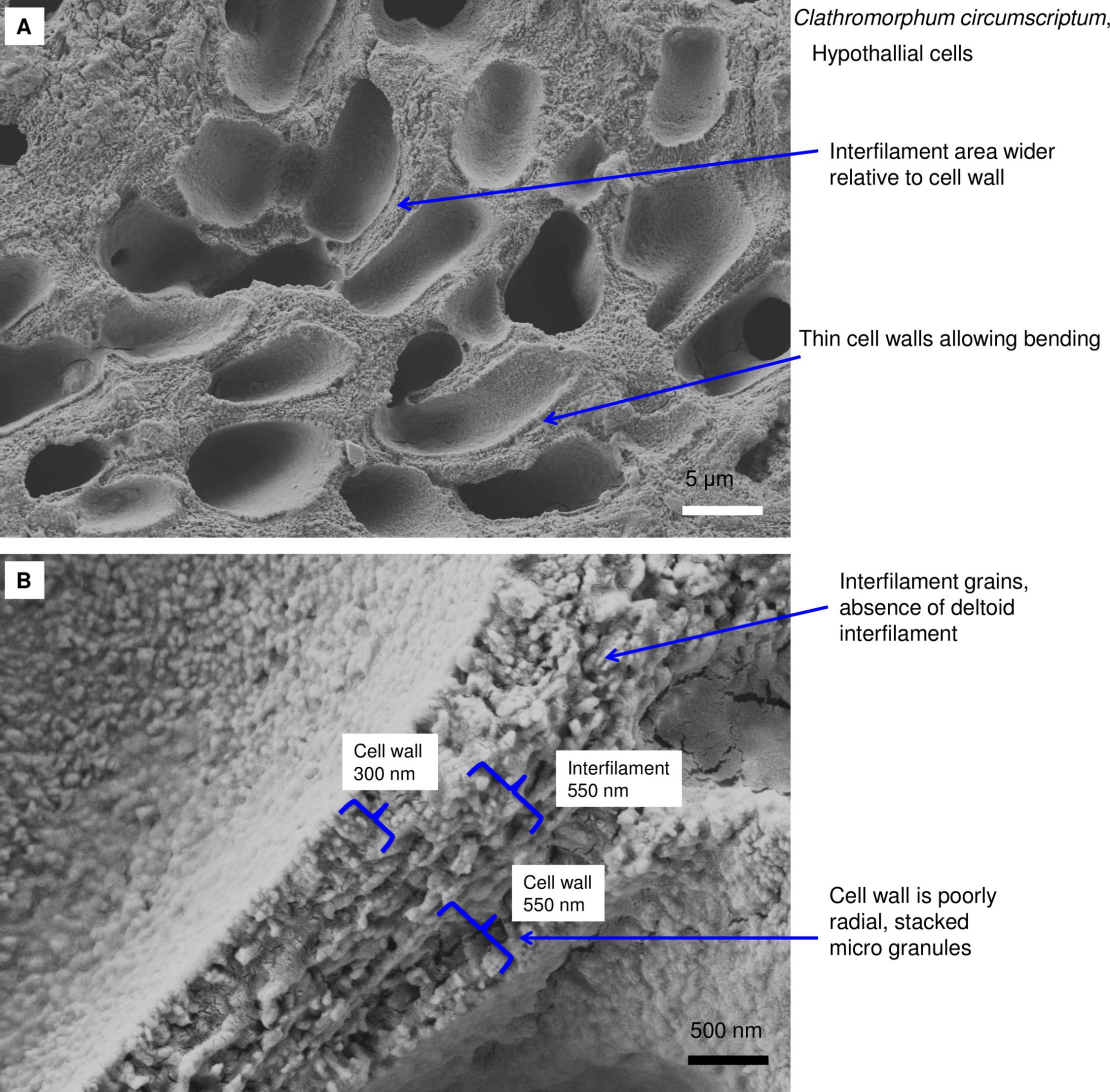
Hypothallial PCW cells in *Clathromorphum circumscriptum*, (Norway). (A) Hypobasal with curved thin cell walls. (B). Cell walls are thinner than perithallial cell walls. There is no radial Mg-calcite present. Interfilament is similar to interfilament in other species and there is no deltoid interfilament as is normally present between *Clathromorphum* perithallial cells.

**Fig 14:**
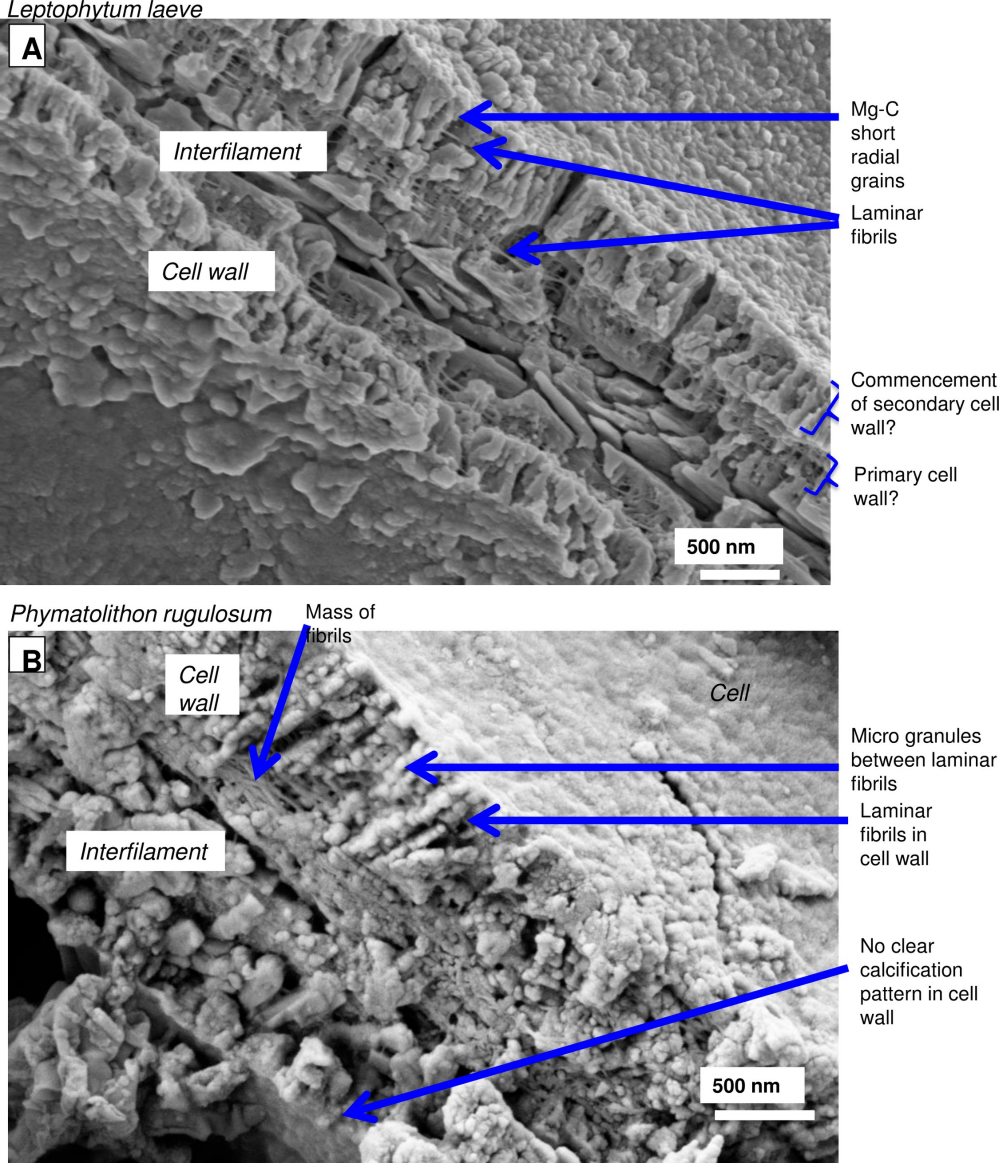
Close up of typical hypothallial cell wall features. (A) Laminar fibrils are present throughout the perithallial cell walls of both *Leptophytum leave* (Labrador). (B) *P*. *rugulosum*, Newfoundland. Cell walls may have vertically aligned Mg-calcite or no clear calcification pattern. Figure A reproduced from [38] Creative Commons Attribution 3.0 License, Copyright Author(s) 2018. Figure B reproduced with permission from [39]).

**Fig 15:**
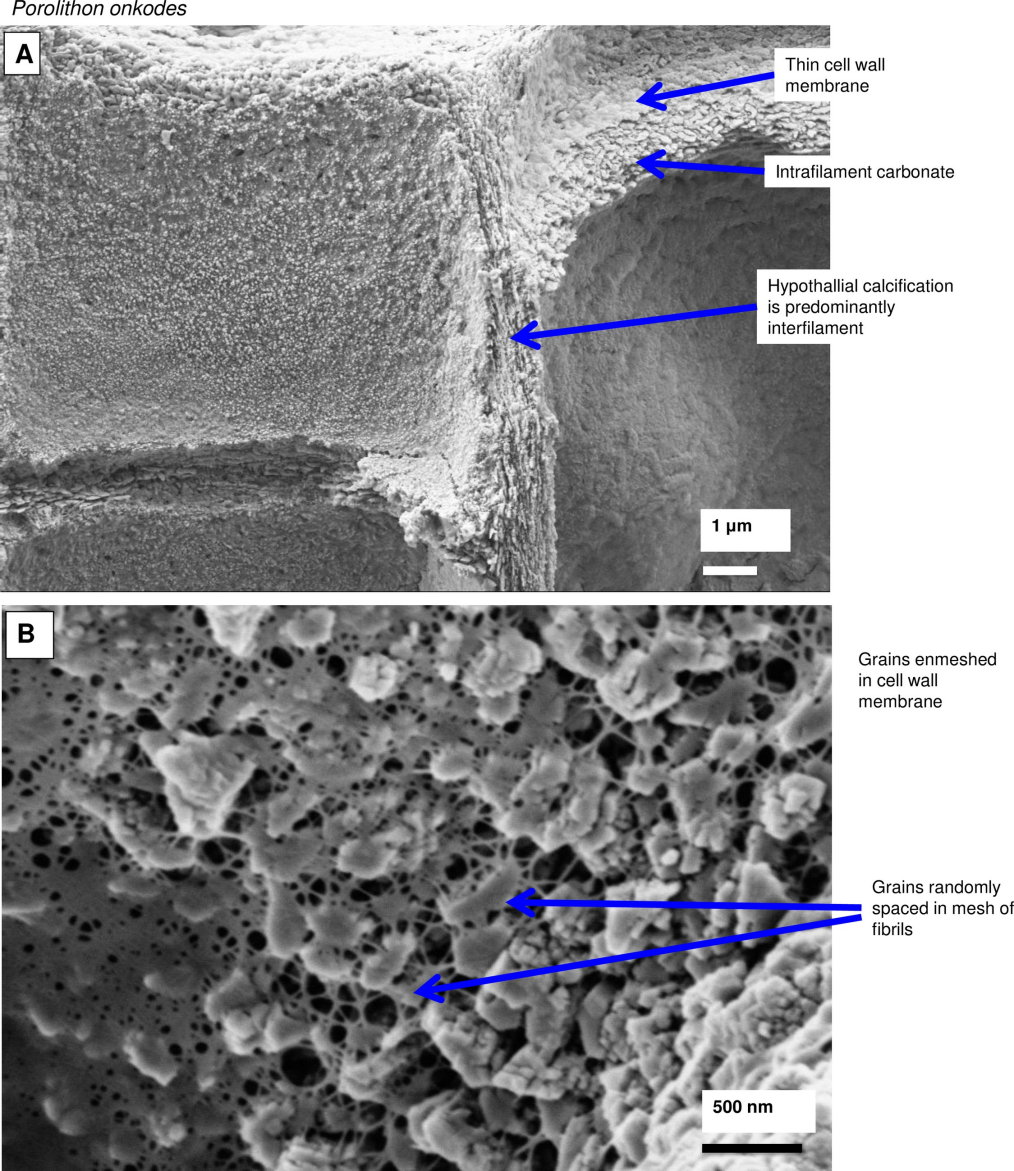
Hypothallial PCW cells in *Porolithon onkodes*, (reef flat, Heron Is. Great Barrier Reef, Australia). (A) No visible cell wall structure. Carbonate is predominantly interfilament or intrafilament. (B) Thin layer of Mg-calcite in PCW. Mg-calcite is randomly spaced and shaped grains enmeshed in a mass of fibrils. Figure reproduced with permission from [10].

**Fig 16:**
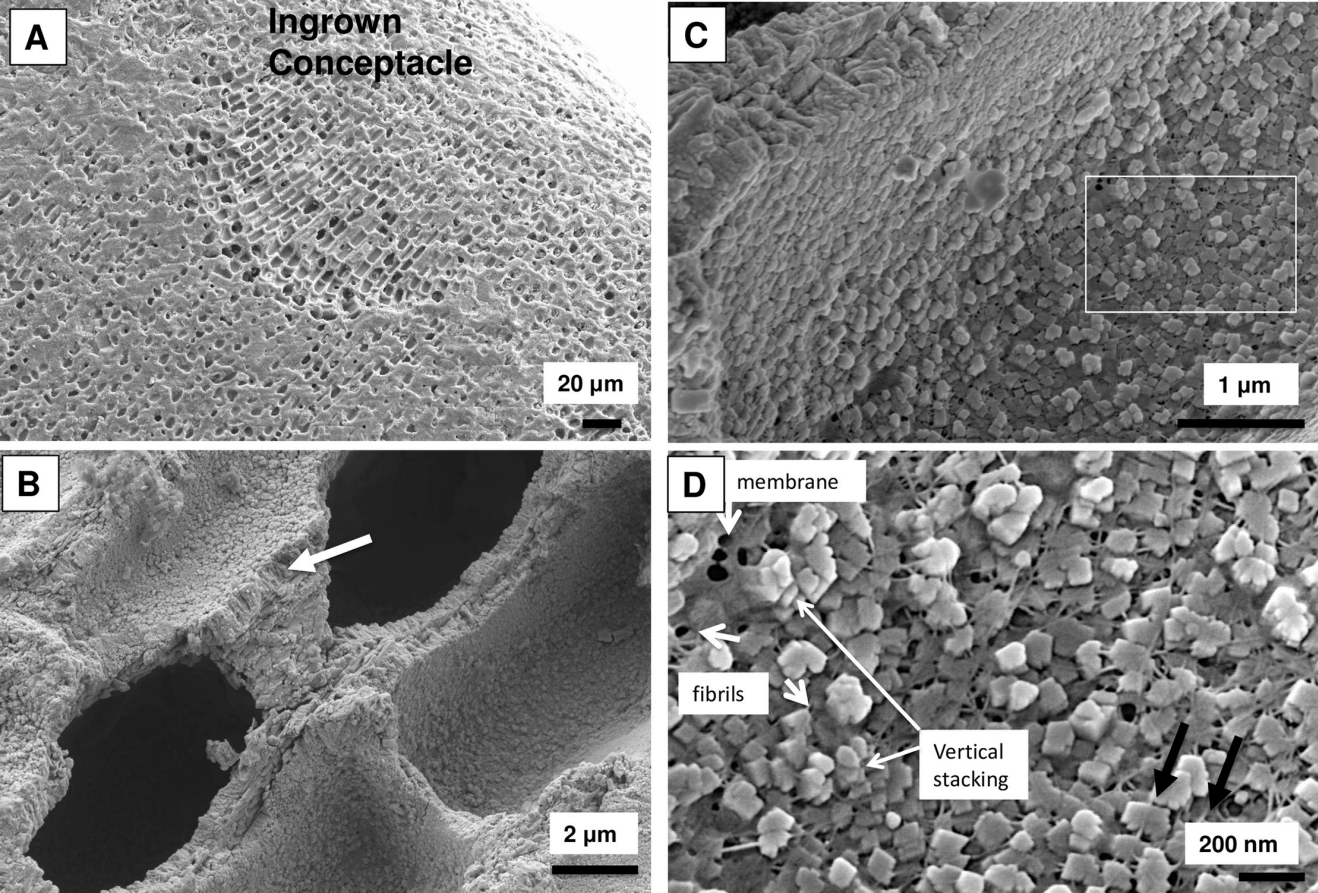
PCW-only cell walls in cells infilling emptied conceptacles of *Phymatolithon rugulosum*, (Iceland). (A) Overview. (B) Cell walls at this scale appear to have radial vertical structure (white arrow). (C) Minimal interfilament between cell walls. White box enlarged in D. (D) Cell wall grains have well defined crystal faces and are enmeshed within a fibrous mesh. Fibrils are visible running through the interior of crystals (black arrows). Image republished from Nash and Adey [39] under a CC BY license, with permission from John Wiley and sons, Copyright 2017.

**Fig 17:**
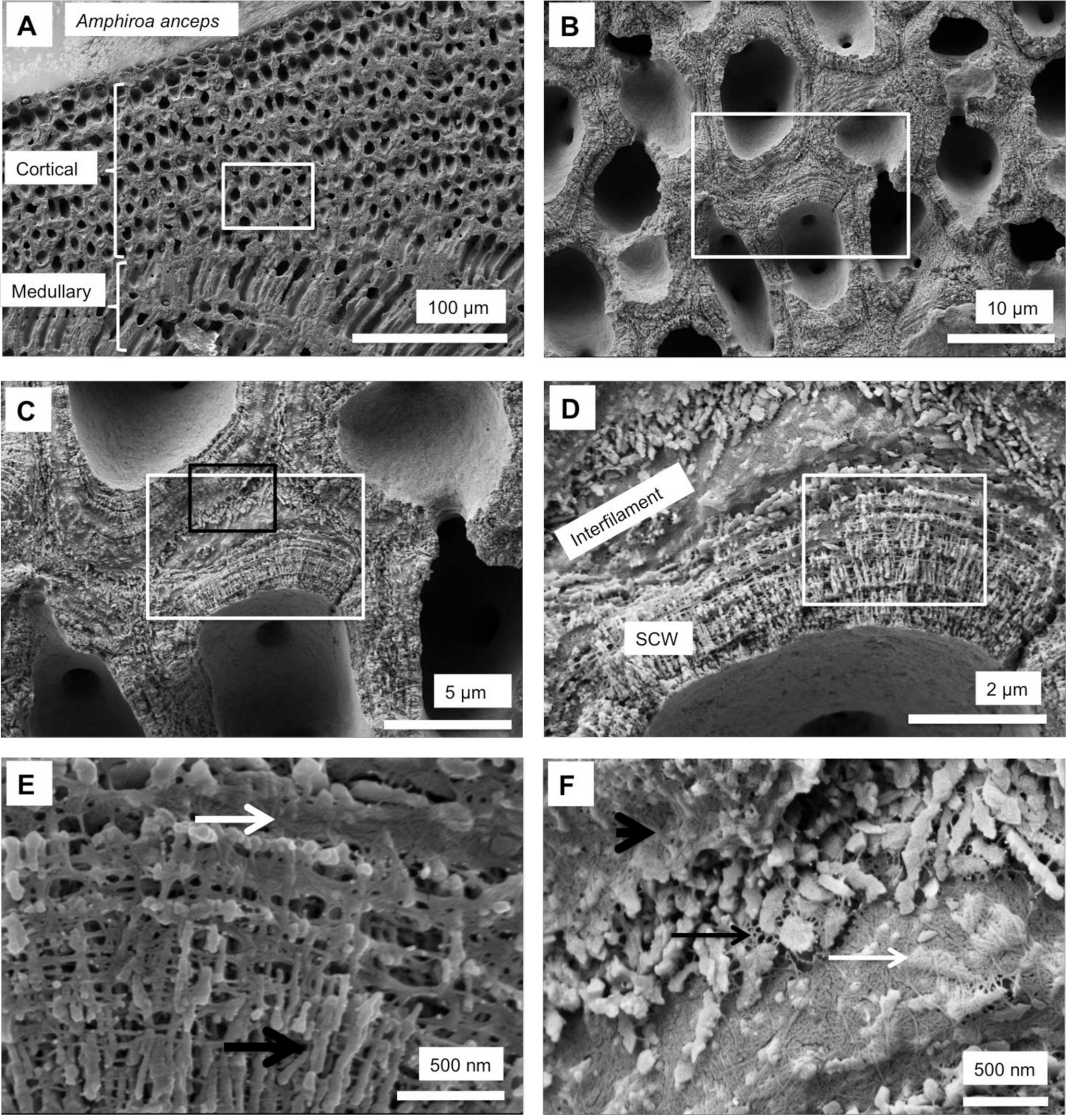
Secondary cell wall in *Amphiroa anceps*, (Warrnambool, Victoria, Australia). Etched for 35 minutes. (A) Overview of cortical cells with secondary cell wall and medullary cells with primary cell walls. White box enlarged in B. (B) Radial Mg-calcite in cell walls. These features were not readably visible in un-etched samples. (C) Overview of features enlarged in D, E (White box) and F (Black box). (D) Interfilament grains are generally rice grain shape with edges that have a serrated appearance. The SCW radial Mg-calcite grains are present as multiple layers of shorter radial grains. White box enlarged in E. (E) Radial grains are consistently cylindrical with smooth sides (black arrow). Laminar fibrils form dense mesh at outer perimeter of cell wall (white arrow). (F) Interfilament grain edge serrations appear to form where fibrils are attached (black arrow). Grains appear to form within the dense mesh (white arrow), although this appearance may be an artifact of the etching. Outer edge of adjacent cell wall bordered by dense mesh of fibrils (black arrowhead).

**Fig 18:**
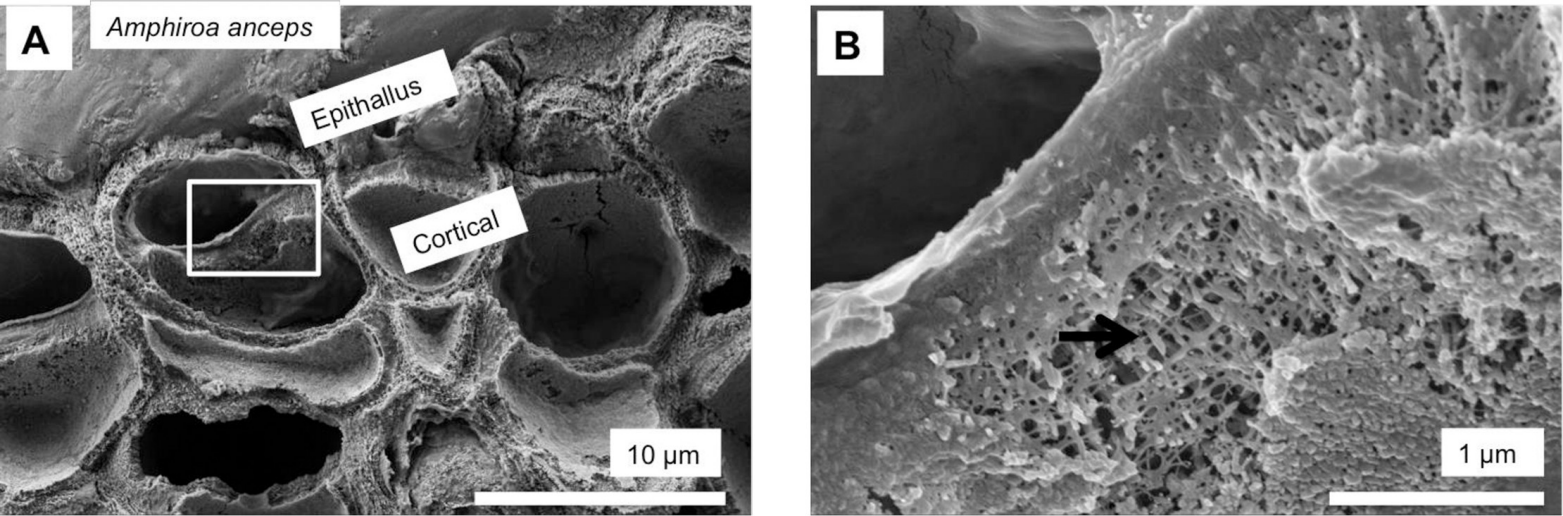
Surficial cells with developing secondary cell walls in *Amphiroa anceps*, (Warrnambool, Victoria, Australia). Sample etched for 35 minutes. (A) Meristem cell forming epithallial cells outwards (up) and cortical cells inwards. White box enlarged in B. (B) The beginnings of radial Mg-calcite development are visible (black arrow) within the bands of laminar fibrils.

**Fig 19:**
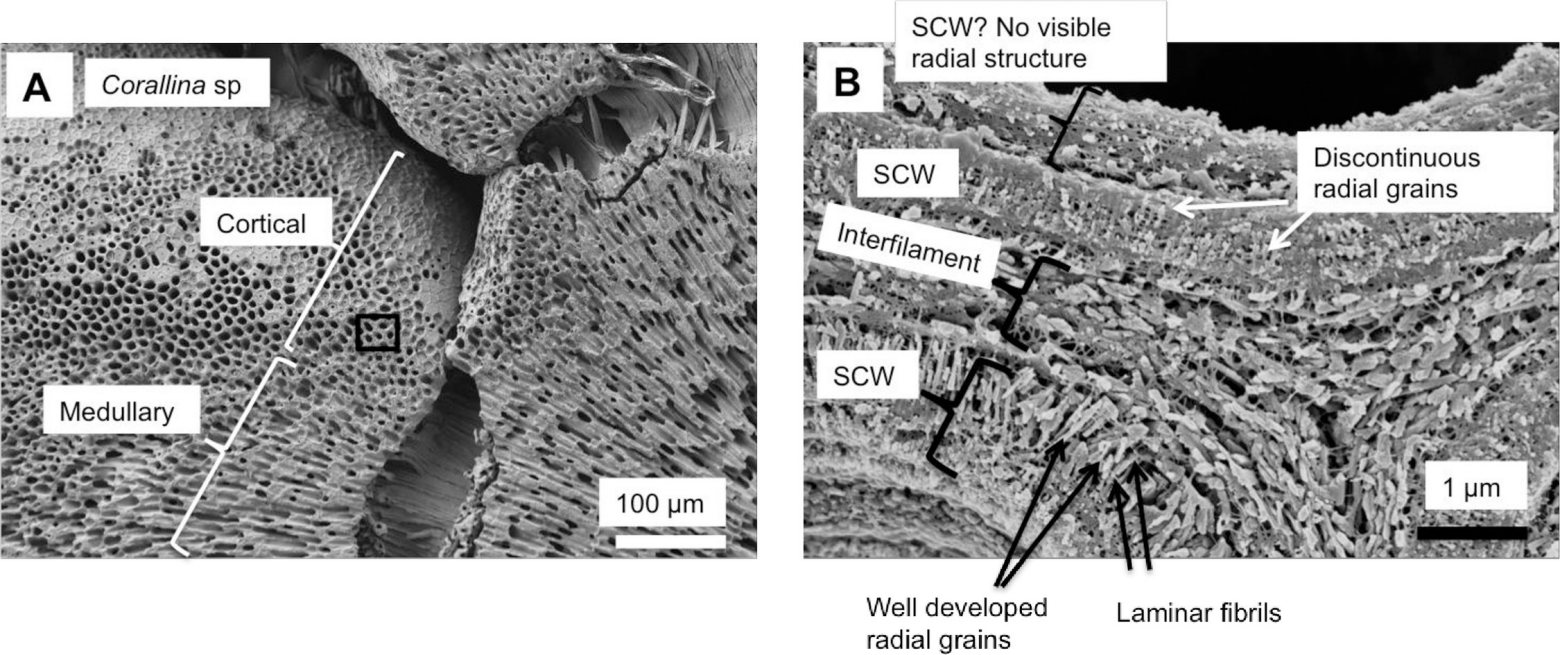
*Corallina* sp., (Coffs Harbour, New South Wales, Australia). Etched for 35 minutes. (A) Overview of cortical and medullary cells. Black box enlarged in B. (B) Lower half of image shows a cortical cell with well-developed secondary cell wall and radial Mg-calcite. The upper cell SCW has radial Mg-calcite in the SCW but the grains are discontinuous. Possibly this may be an artifact of etching. There is a separate inner wall that may be a poorly-developed part of the SCW. There is no visible radial structure.

**Fig 20:**
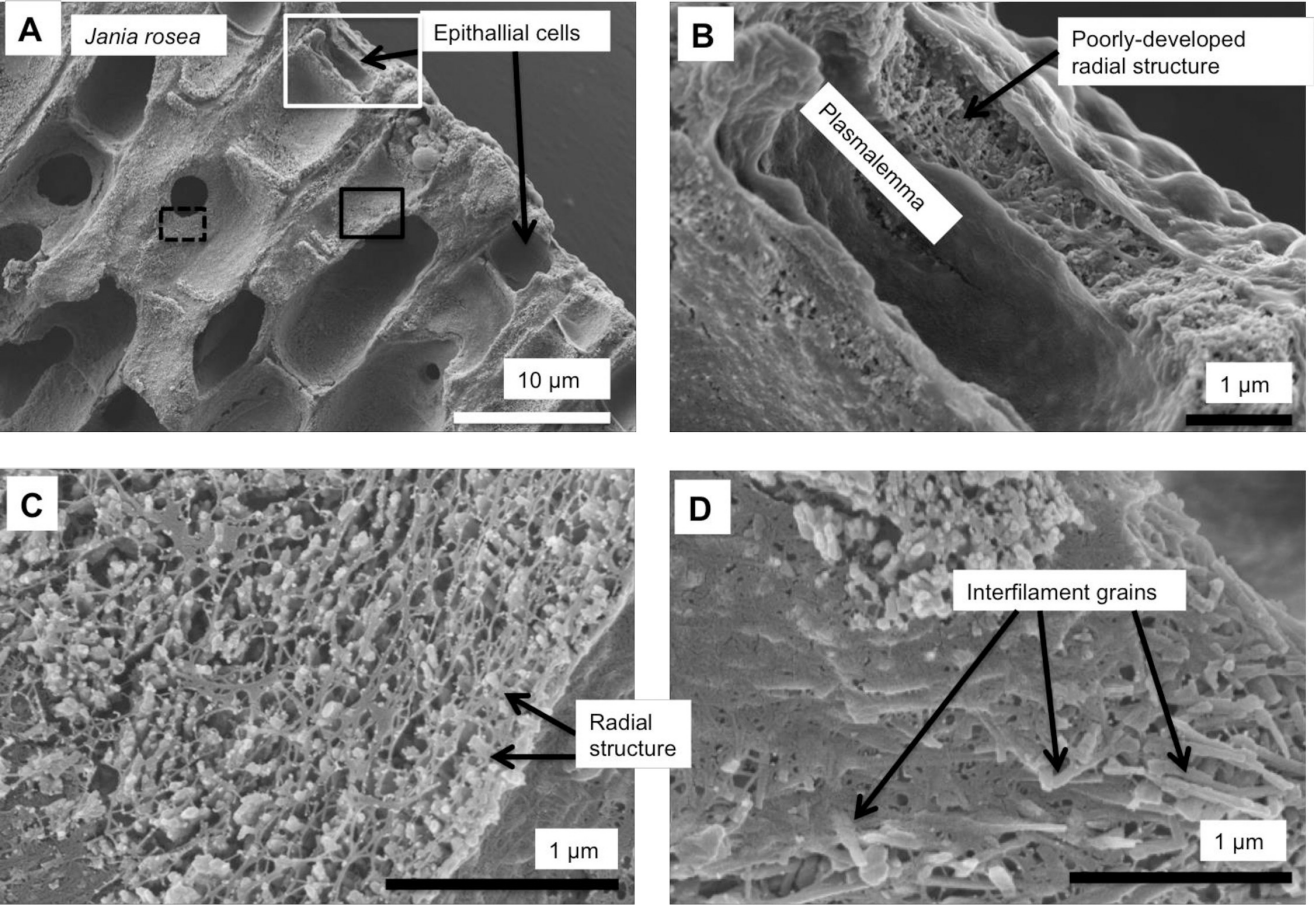
Possible development of SCW and interfilament in *Jania rosea*, (Tathra, New South Wales, Australia). Sample etched for 35 minutes. (A) Overview of surficial cells. White box enlarged in B. Black box enlarged in C. Black dashed box enlarged in D. (B) Epithallial cell appears to have a poorly-developed radial structure. It is not clear if this is a typical SCW radial Mg-calcite. (C) Partial growth of Mg-calcite perpendicular to cell wall. This may be poorly-developed SCW. (D) Interfilament grains are visible through the cell wall fibrillar mesh. These grains are comparable to the rice-shaped grains in other species but are generally longer (~ 500 nm compared to ~200 nm in most species) similarly to the elongate flat grains on surficial cells (Fig 11).

**Fig 21:**
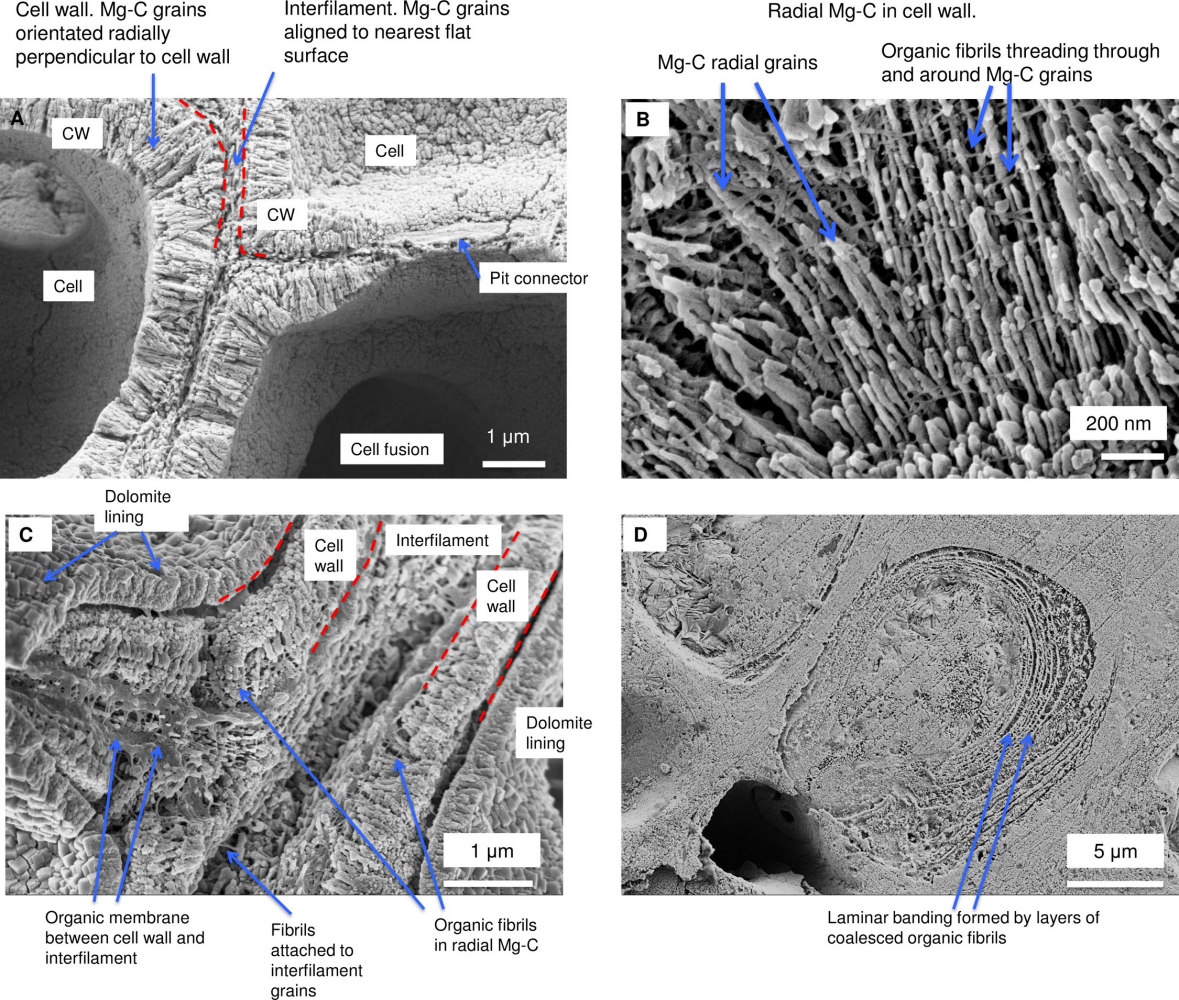
Perithallial cellular calcification features in *Porolithon onkodes*, (Heron Is., Great Barrier Reef, Australia [10]). (A) Perithallial cell immediately beneath epithallus. (B) Radial grains within cell wall have semi-regularly spaced organic fibrils threaded through and around the Mg-calcite radial grains. This location in the crust had been naturally etched by micro-boring activity. (C) The boundary between the cell wall and interfilament has both a fibrillar mesh and patches of organic membrane. Sample was etched for 50 minutes in deionised water then sonic cleaned for 2 minutes. Most of the interfilament carbonate is removed by this preparation processes. (D) This site is near the base of the crust and has been exposed to seawater. The cell wall and interfilament has undergone remineralisation. However, the cell wall fibrils appear to have coalesced and formed distinct laminar bands. Panels A, B, D reproduced with permission from [10].

**Fig 22:**
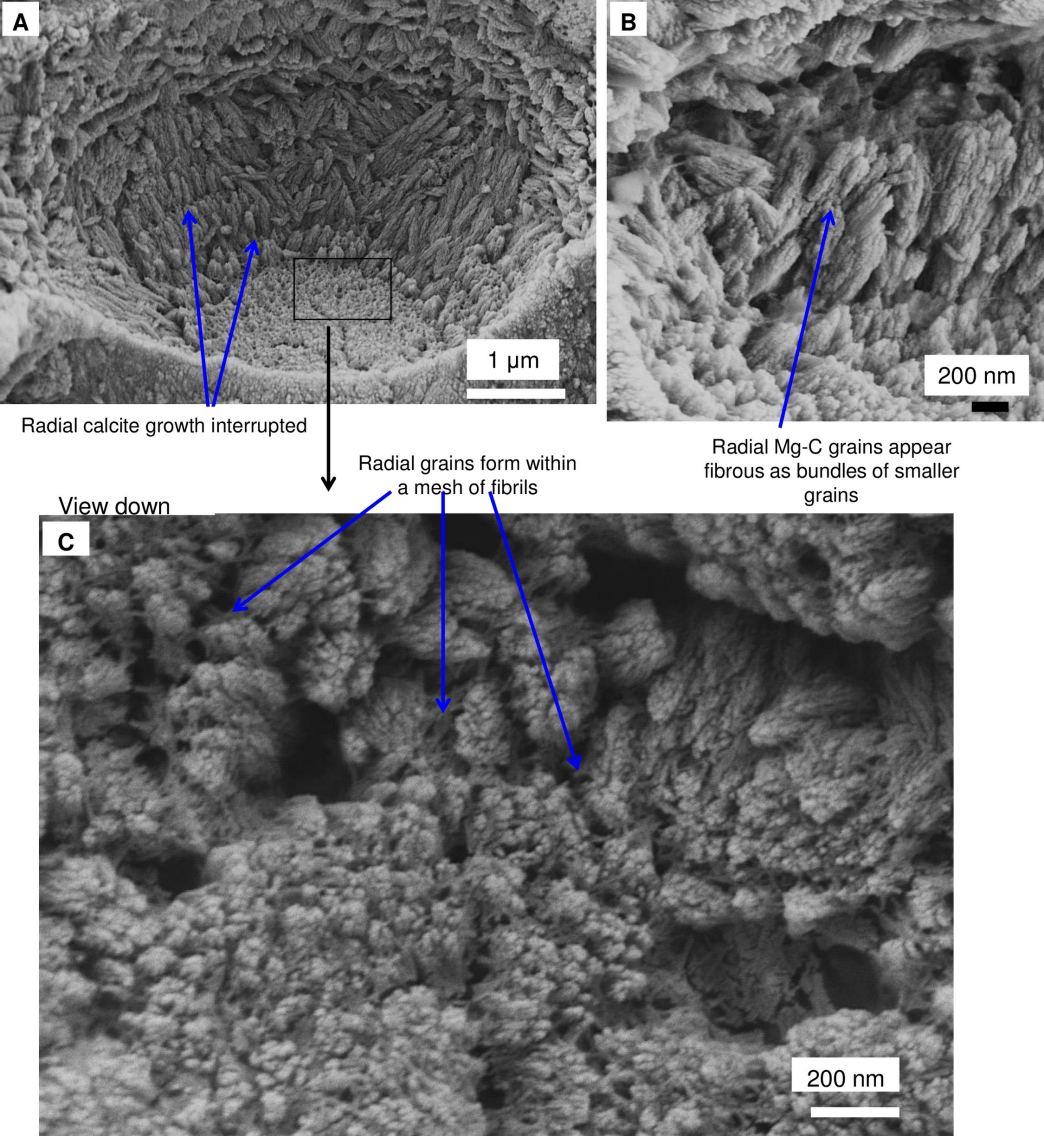
*Porolithon onkodes*, (Heron Is. Great Barrier Reef, Australia [10]). Cell wall exposed by internal bioerosion etching the surface. (A) Radial Mg-calcite is segmented smaller grains instead of a long continuous grain as in previous image. Possibly the joint lines are the site of fibrils removed by the etching. (B) Grains appear fibrous as if bundles of smaller thinner grains. (C) View into vertically-facing radial Mg-calcite grains. A mass of fibrils enmesh the Mg-calcite grains. Spaces between radial grains are filled with irregularly shaped Mg-calcite nano-granules. Figure adapted with permission from [10].

**Fig 23:**
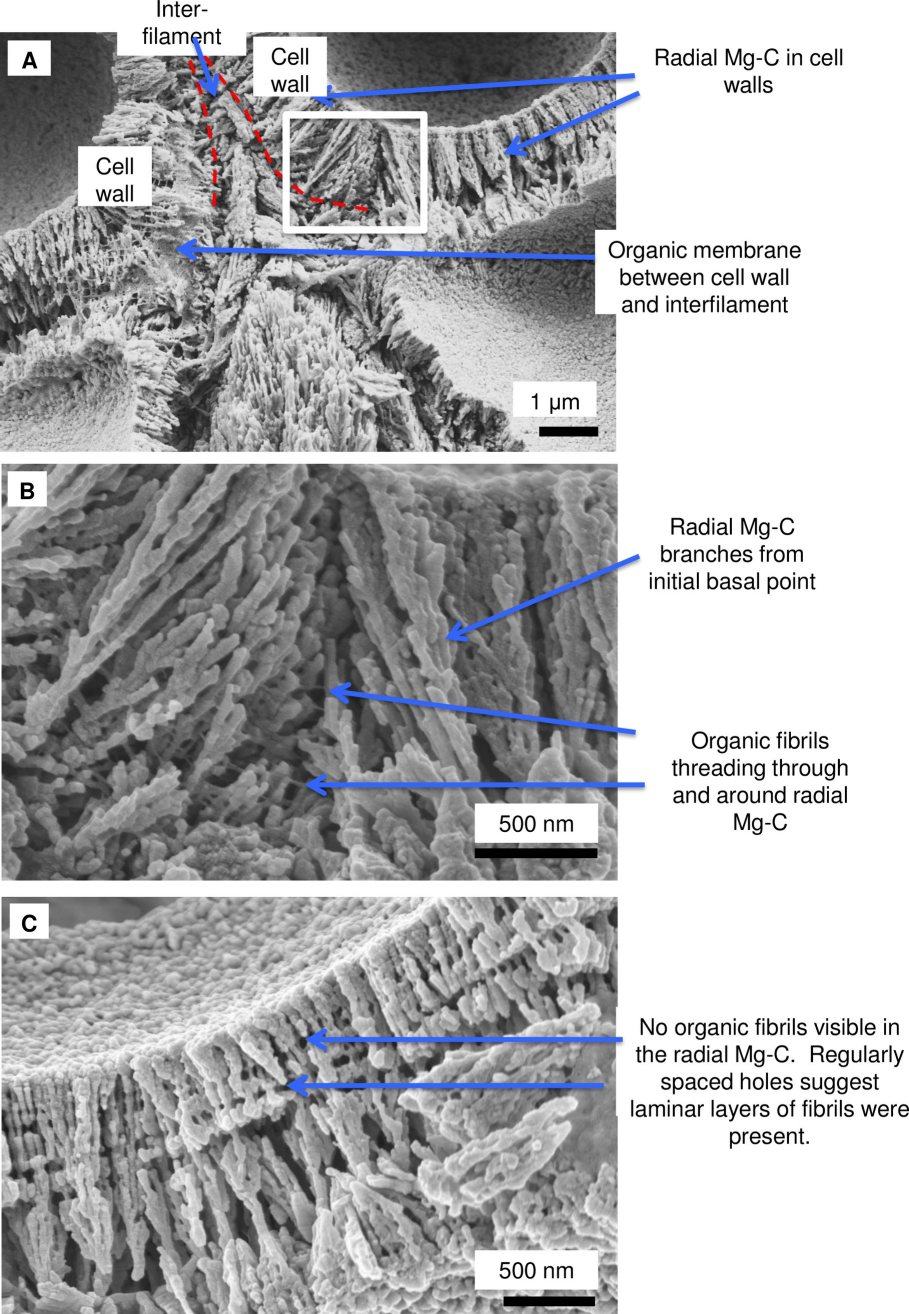
Perithallial cell wall in *Clathromorphum compactum*, (Greenland). Sample etched 20 minutes in deionised water, sonic cleaned for 2 minutes. Approximately 300 microns below surface. (A) Radial Mg-calcite in cell wall. There is a patch of membrane between the cell wall and interfilament. White box enlarged in B. (B) Organic fibrils thread between and through the Mg-calcite grains. (C) Laminar banding of regularly spaced holes where fibrils presumably were present prior to etching.

**Fig 24:**
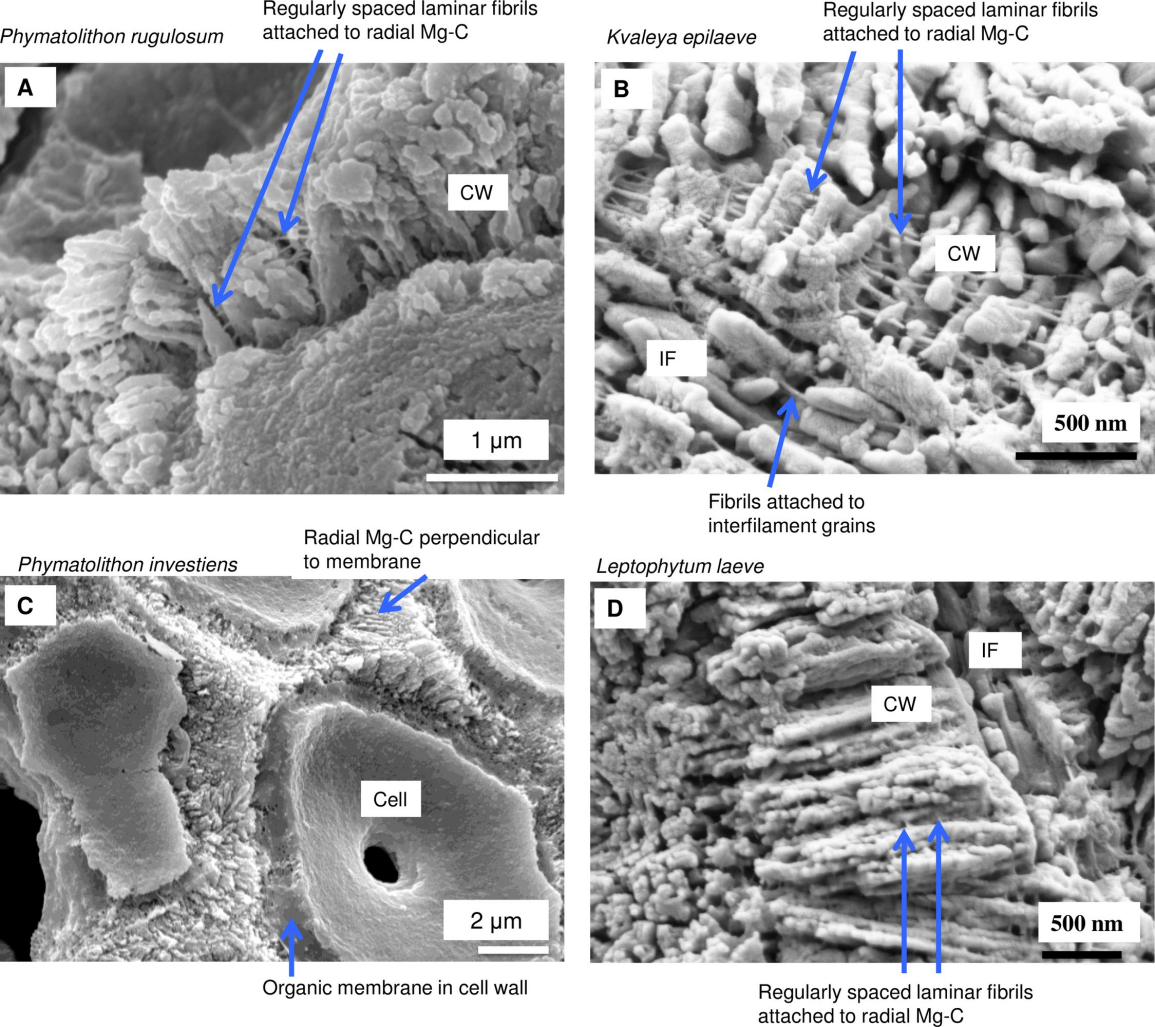
Northern boreal CCA species with radial calcite and microfibrils. (A) *Phymatolithon rugulosum* (Newfoundland). (B) *Kvaleya epilaeve* (Labrador). (C) *Phymatolithon investiens* (North Norway). (D) *Leptophytum leave* (Labrador). The radial Mg-calcite in *K*. *epilaeve* and *L*. *leave* is thicker (100 nm and 60 nm) in diameter than the *P*. *onkodes* (20–25 nm) and C*lathromorphum* (40–50 nm). Images for *Phymatolithon* species republished from Nash and Adey [39] under a CC BY license, with permission from John Wiley and sons, Copyright 2017. *K*. *epilaeve* and *L*. *leave* Nash and Adey [38] under a CC BY license, with permission from Copernicus, Copyright 2017.

As in higher plants, coralline algae can have both a primary cell wall (PCW) and secondary cell wall (SCW) (Fig 2), similarly to plants. The PCW is solely present in fast growing elongated cells (Fig 6). These PCW-only cells form the central medullary cells in articulated coralline algae (Figs 7–12) and the hypothallial (Fig 13–15), wound repair (Fig 6) and conceptacle ingrowth cells (Fig 16) in CCA. The SCW is present in addition to the PCW in the cortical cells of the articulated corallines (Figs 17–20) and the CCA perithallial cells (Figs 21–24). The PCW is thin (< 500 nm) and may be poorly to densely calcified with micro-granules of Mg-calcite, and occasionally is without calcification. The SCW is thick (1–2 microns) and is consistently comprised of radial Mg-calcite with micro-granules filling remnant spaces. The PCW carbonate varies in crystal size and shape. These carbonate granules may be densely infilling the PCW (Fig 7), present as vertical stacking of micro-granules (Figs 7 and 16), poorly infilling with grains spaced apart on a fibril mesh (Figs 9, 11, 15 and 16), and as an apparent mineral infill within a membrane (Fig 7). Bands of fibrils (~10–20 nm width), where visible, are parallel to the cell wall (Fig 14), with thinner fibrils present throughout the PCW forming a mesh (Fig 12). The PCW cell wall thickness ranges from ~50 nm to ~500 nm. The wider PCW in CCA may be incorporating the start of the SCW (Fig 14). There is a notable species difference amongst the articulated corallines. *Amphiroa* PCW (Figs 7 and 8) are more densely calcified than those of *Corallina* (Figs 9 and 10) and *Jania* (Figs 11 and 12) and appear to be less prone to breaking. The medullary walls of the *Corallina* and *Jania* are poorly developed and the boundary between the external edge of the cell wall, the middle lamella and adjacent medullary cell is not always visible (Fig 11). The production of the SCW in the CCA perithallial cells starts with the addition of a wider thickness of carbonate (Fig 21). The radial grains generally appear as a continuous grain (Figs 21, 23 and 24), but may also be present as continuous series of connected stacked grains (Figs 17 and 22). The radial grains appear to be comprised of clumped smaller fibrous grains (Fig 22). Etching removes smaller space-filling carbonate granules and reveals the laminar bands of fibrils throughout the SCW (Figs 17–19 and 21–23) and in a concentrated mass around the cell wall perimeter. Thinner fibrils weave apparently randomly throughout the SCW (Fig 22). The fibrils are seen both on the surface of the radial grains and appearing to be encapsulated within the grain (Figs 17–19 and 21) and threading through it, similarly to wire through a fence post (Figs 17 and 21). The corners of the articulated medullary cells may have partially formed SCW (Fig 20).

### Primary cell wall

### Secondary cell wall

#### Epiphytes

**Fig 25:**
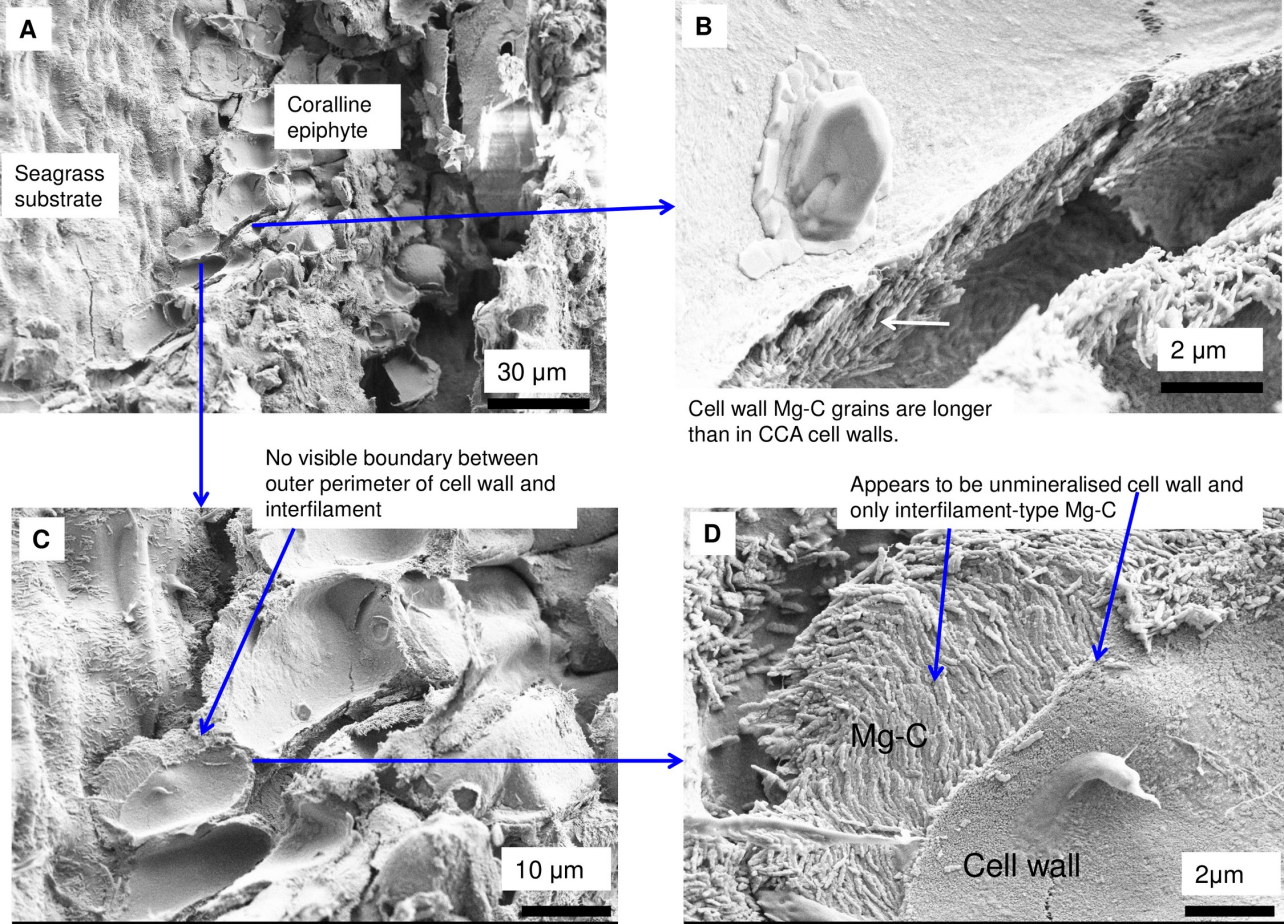
Coralline epiphytes (Mediterranean, samples from [94]). (A) The epiphytes form a single cell layer on the seagrass substrate. (C) There is no clear boundary between a calcified cell wall and interfilament. (B, D) The cell wall appears not to be calcified and elongate Mg-calcite, similar in form to CCA interfilament, Mg-calcite fills space between cell wall and seagrass surface.

**Fig 26:**
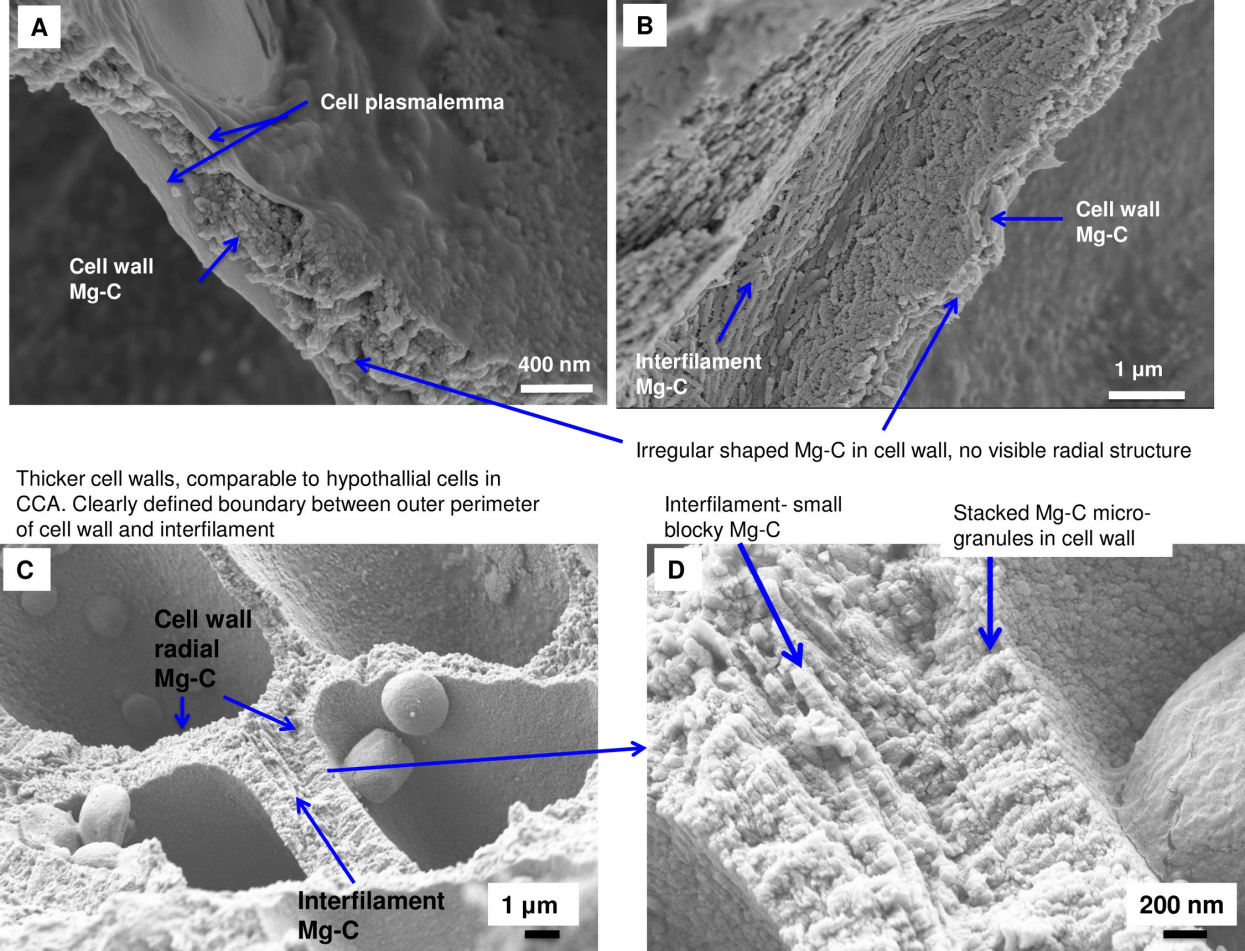
Coralline epiphytes (Mediterranean, samples from [94]). **(**A, B) Calcification within the primary cell wall and interfilament. (C, D) Parts of the coralline epiphyte have well developed PCW calcification comparable to CCA PCW.

In contrast, in epiphyte CCA the primary cell wall may be uncalcified with the only carbonate present being interfilament grains or it may have thin calcified PCW similarly to CCA hypothallial cells [94] (Figs 25 and 26).

#### Formation of interfilament carbonate

**Fig 27:**
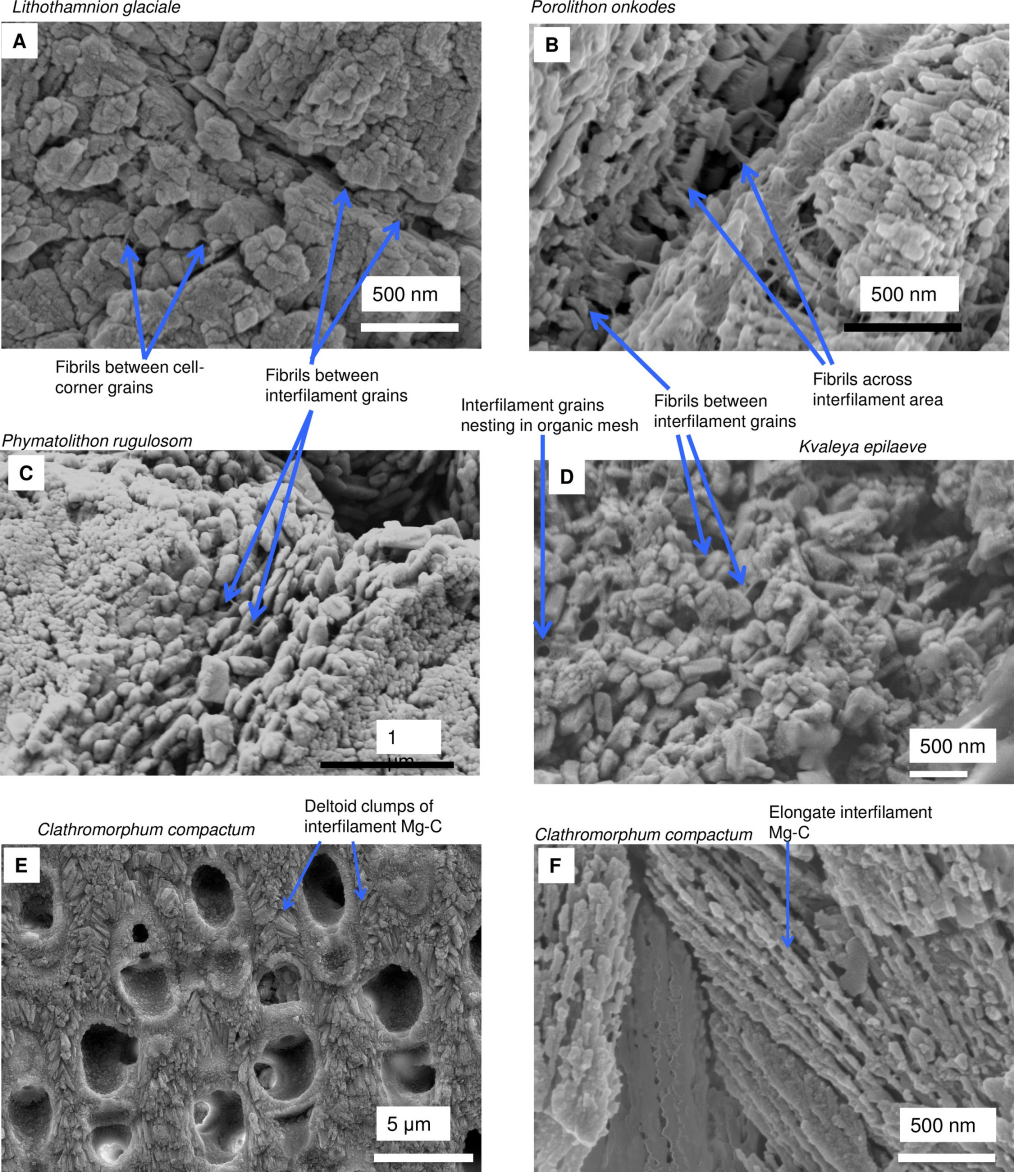
Mg-calcite and fibrils in the interfilament. (A) *Lithothamnion glaciale* (Scotland, sample from [18]). (B) *Porolithon onkodes* (Heron Is. Great Barrier Reef, Australia). (C) *Phymatolithon rugulosom* (Newfoundland). (D) *Kvaleya epilaeve* (Labrador). (E, F) *Clathromorphum compactum* (Greenland) with distinctive deltoid interfilament made of clumps of elongate thin cylindrical Mg-calcite. Images for *Phymatolithon* species republished from Nash and Adey [39] under a CC BY license, with permission from John Wiley and sons, Copyright 2017. *K*. *epilaeve* and *L*. *leave* Nash and Adey [38] under a CC BY license, with permission from Copernicus, Copyright 2017.

Interfilament carbonate is that forming in the area between both vertically and horizontally adjacent cells, between the base and top of cells within a filament, and between the side-walls of cells in adjacent filaments. Interfilament carbonate is typically rice-grain-shaped Mg-calcite, sub-micron length, generally orientated parallel to the proximal cell wall surface (Fig 20) and may have serrated edges (Fig 17). These features are typical for all genera but *Clathromorphum*. Thin fibrils connecting grains may be visible (Fig 27). In *Clathromorphum*, elongate (2–3 microns) thin cylindrical grains clump together into a deltoid formation, this is discussed in detail in the *Clathromorphum* calcification model paragraphs. In CCA epiphytes (Fig 25) where the PCW is uncalcified, longer (~1–2 microns) interfilament grains are typical. Interfilament between calcified cells of epiphytes can be small blocky grains (Fig 26). Interfilament carbonate is consistently present between CCA perithallial (Figs 21, 23 and 24) and hypothallial (Figs 6 and 13–15) cells. However interfilament is minimal or absent between CCA wound repair (Fig 6C–6E) and conceptacle infill cells (Fig 16). Articulated corallines have substantially less interfilament calcification present and it is generally absent or minimal between the long edges of medullary cells (Figs 8–10 and 12); it is predominantly present at the junctions of cells (Fig 8).

#### Magnesium content

**Fig 28:**
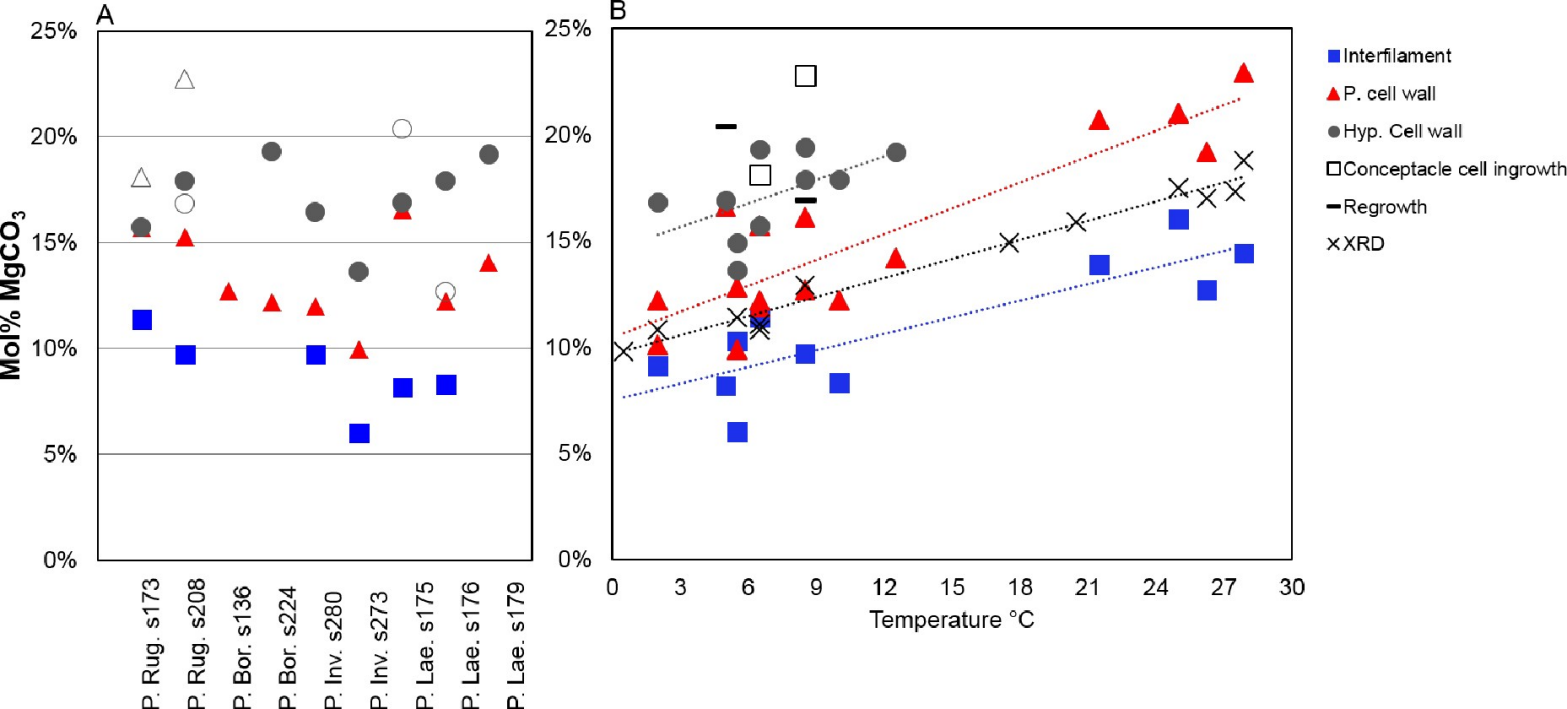
Mg-content of PCW, SCW and interfilament of *Phymatolithon Rugulosum*, *P*. *borealis*, *P*. *investiens*, *P*. *laevigatum*. There is a consistent offset in Mg-content between the SCW (perithallial cell wall) and interfilament. PCW (hypothallial cell wall) Mg-content is higher but the offset from PCW is not consistent. P. cell wall: perithallial cell wall. Hyp. cell wall: hypothallial cell wall. Image republished from Nash and Adey [39] under a CC BY license, with permission from John Wiley and sons, Copyright 2017).

**Fig 29:**
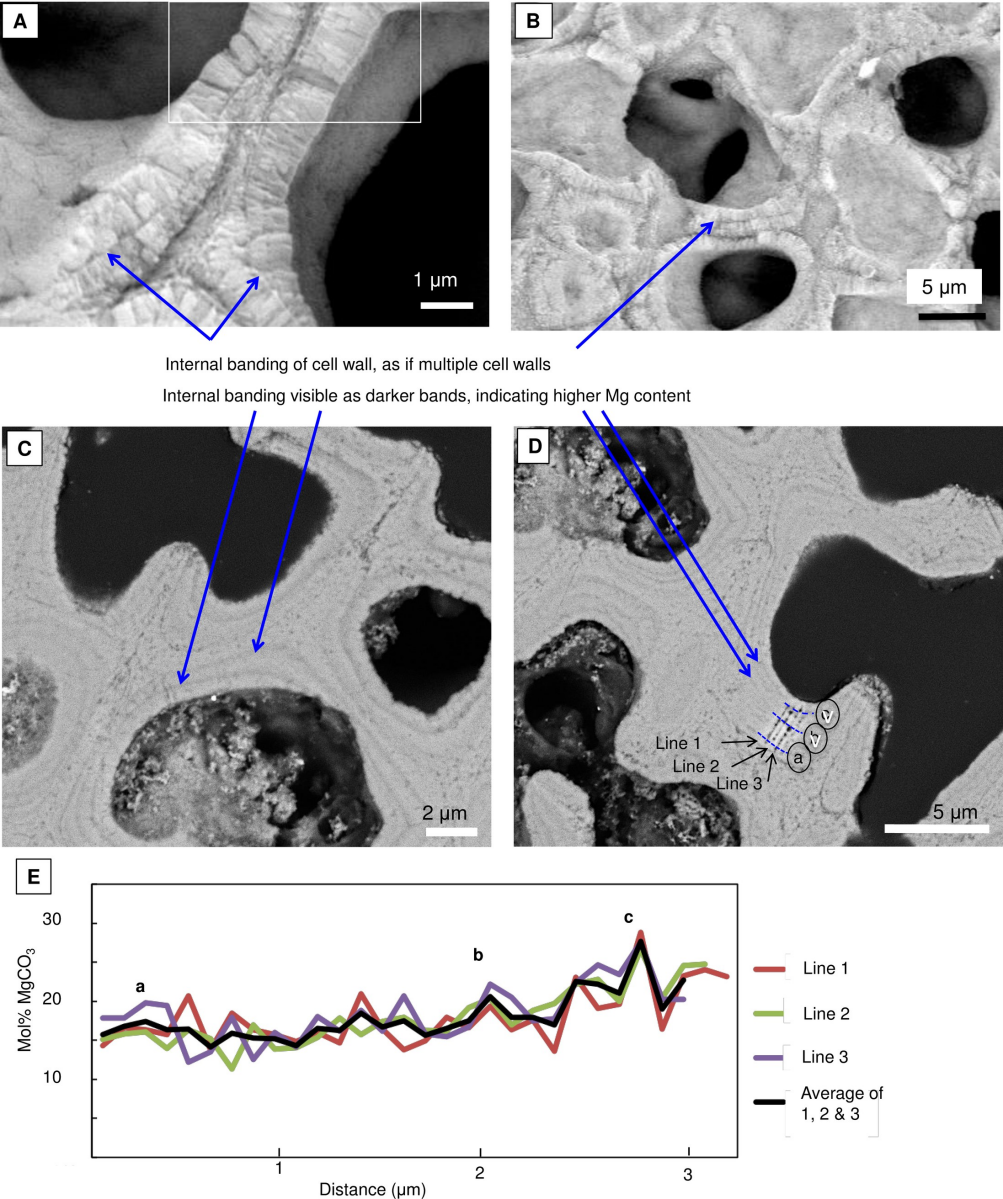
SCW and Mg-content in *Porolithon onkodes* (Heron Is. Great Barrier Reef, Australia). (A, B) Cell wall in *P*. *onkodes* with internal banding possibly from an interruption in growth resulting in segmentation of the radial Mg-calcite. White box is image in Fig 21A. (C, D) SEM (carbon coated as this is best for showing the contrast between Mg and Ca whereas platinum is better for imaging). (C) Dark bands are associated with the internal edges of the cell wall segmentations. Dark bands have elevated Mg. Bands are ~0.6 to 1 micron apart. (D) Site for EDS lines, letters are matching mol% MgCO_3_ values in E. (E) EDS lines across the cell wall in D. Highest Mg bands labeled. Peaks of high Mg are ~ 300–400 nm apart. Lines 4, 5, 6 are top, middle and bottom EDS lines in D.

The Mg content is higher in PCW relative to the SCW within the same crust (Fig 28) [10,38,39]. In CCA perithallial cells, where bands of higher Mg content are present, these appear to be the portion of PCW at the outer and inner perimeter of the SCW (Fig 29). Any additional elevated-Mg bands within the SCW are at sites where the wall appears segmented suggesting there may have been a growth interruption in the radial calcite (Fig 29) and temporary reversion to PCW. The higher Mg values recorded in the cell wall bands range from very high magnesium calcite (VHMC) (25–36 mol% MgCO_3_) up to dolomite composition (>37 mol% MgCO_3_ [95,96]). These higher Mg content carbonates are grouped together and referred to as ‘D-type’ carbonate. D-type is used to reflect their range of Mg composition from higher Mg-calcite up to dolomite composition, but to differentiate from dolomite composition carbonate found as cell lining in *Porolithon onkodes* [8]. D-type was found in *Hydrolithon*, *Porolithon*, *Lithothamnion*, *Lithophyllum*, *Mesophyllum*, *Tenarea*, *Pneophyllum*, *Spongites* and *Amphiroa* species (S3 Table).

**Fig 30:**
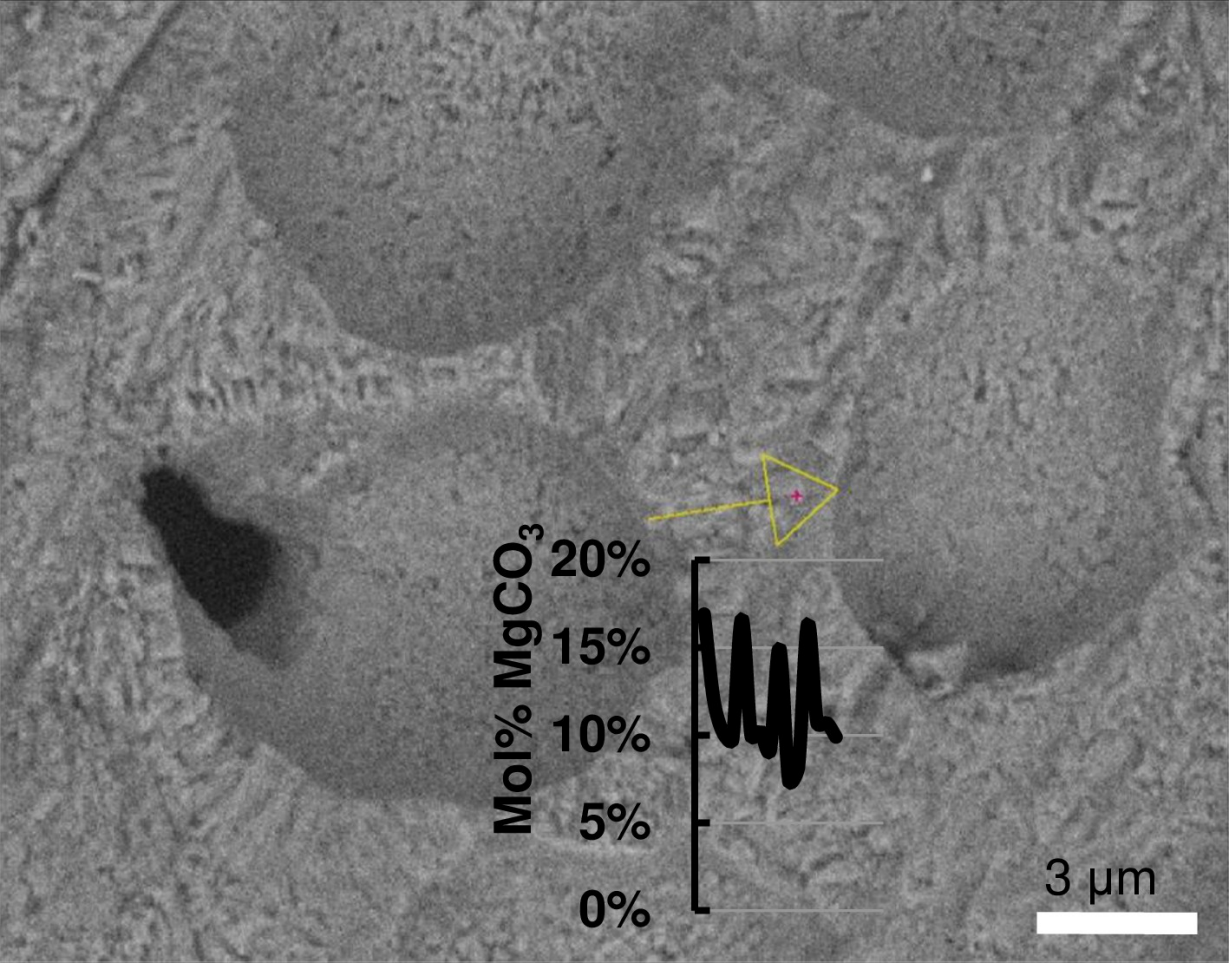
*Leptophytum laeve* (sample from Nash and Adey [38]) SEM-EDS line. Mg content range 7.4–16.9 mol% MgCO_3_. Highest Mg content at internal edges and near external edges of cell walls. Yellow arrow- EDS line, 14 sample points.

While elevated Mg around the perimeter of the cell wall was prevalent in all samples tested, not all CCA had Mg-content elevated to D-type levels. *Leptophytum laeve* (Fig 30), an Arctic species, had a range of Mg of 7.4–16.9 mol% MgCO_3_ in the perithallial cell wall with the highest values at the perimeters. Because the higher Mg carbonate is present differently to the bulk of the radial and interfilament Mg-calcite, but does not reach the high values for D-type grouping, we propose to refer to these bands of elevated Mg as ‘M-type’. M-type was found in *Leptophytum*, *Phymatolithon*, *Hydrolithon*, *Sporolithon*, *Neogoniolithon*, *Mastophora*, *Metagoniolithon*, *Jania*, *Corallina*, *Lithothrix* and *Mesophyllum* (S3 Table). *Clathromorphum* species were predominantly M-type, but two samples had elevated Mg in the D-type range in some hypothallial cells indicating that there was the capacity to form D-type carbonate, but typically it was M-type.

With the possible exception of the *Clathromophum* sp. (S2 Table), interfilament carbonate always has lower average Mg-content than the cell wall (Fig 28) [38,39]. Analyses of *Clathromorphum* sp. found areas of crust where cell wall had higher Mg as well as areas where cell wall Mg content was not significantly different from interfilament Mg-content.

### Matrix fluids and biomineralisation model

#### Organics and biomineralisation

From our detailed SEM imaging from this and other studies, knowledge of plant anatomy and organic composition, and mineral formation processes demonstrated using a cellulose substrate, we propose the following process for the interaction between organics and biomineralisation. The structural organics within the PCW are predominantly hemicellulose microfibrils whereas the SCW has additionally, cellulose as extruded cellulose microfibrils (CMF) (Fig 3), similarly to other plants. The outer perimeter of the cell wall is a permeable porous mesh of fibrils (Figs 3A and 17) similarly to plants [77] whereas the inner perimeter is bounded by the impermeable plasmalemma lipid membrane. Based on the absence of impermeable barriers other than the plasmalemma, we propose that seawater penetrates down the interfilament, through the outer perimeter of the cell wall, mixing with the organism-produced fluids resulting in a mixed matrix fluid that enables Mg-calcite mineral formation. The particular matrix fluid composition would at the least include sulfated xylogalactans, but not 3,6-anhydrosgalactose. We propose that the PCW carbonate (Fig 3A) is predominantly a mineral formation *on*, rather than *of*, an organic surface. That is, micro-granules precipitate out of the mixed matrix fluid *onto* any available surfaces. We base this proposal on the small grain size, range of crystal shapes formed, and sometimes absences of mineral formation. There may be mineralisation of hemicellulose fibrils, however this either does not happen as they are extruded, or the mineral formation is not robust enough to cement the fibrils perpendicular.

We propose that in the SCW (Fig 3A and 3B), as the CMF is extruded, it soaks in the mixed matrix fluid and becomes mineralised while it is still perpendicular to the cell wall membrane, forming the radial Mg-calcite (Fig 3). As bone mineral growth experiments have shown, cellulose can form a substrate for mineral formation after being soaked in a calcium-rich fluid (0.05M CaCl_2_, [91]) then placed in simulated body fluids (noting that this Ca concentration is higher than in seawater and the concentration at the site of calcification in corallines is not known). Considering that the cellulose extruded in the SCW would be immediately exposed to the calcium and magnesium in seawater, it is reasonable to consider probable that this exposure together with the coralline matrix fluids results in mineralisation of the cellulose. If this were to occur rapidly after the cellulose was extruded, then the CMF would be mineralised in the perpendicular position (Fig 3B and 3C) and not able to fold over and run parallel as it does in plants and fleshy algae (Fig 1). The key difference between the SCW and PCW is that the SCW radial calcite is mineralisation *of* the organic substrate, whereas the PCW carbonate granules are precipitating *on* the organic substrate. Within all the corallines, smaller micro-granules fill in spaces between the radial grains. Most probably these granules precipitate out from the mixed matrix fluid, and the hemicellulose and cellulose fibrils provide an initial nucleating substrate [86]. These micro-granules were readily removed by etching for 20 minutes in deionised water. The ease of dissolution relative to the radial Mg-calcite would in part be due to their smaller size, but also suggests that radial calcite has a degree of protection as would be conferred by an organic scaffold.

It is not clear from this study how the interfilament carbonate forms. Interfilament is readily removed by etching, lending support to interfilament being a precipitate, or having substantially less organic matrix than the radial Mg-calcite.

### *Clathromorphum* calcification model

**Fig 31:**
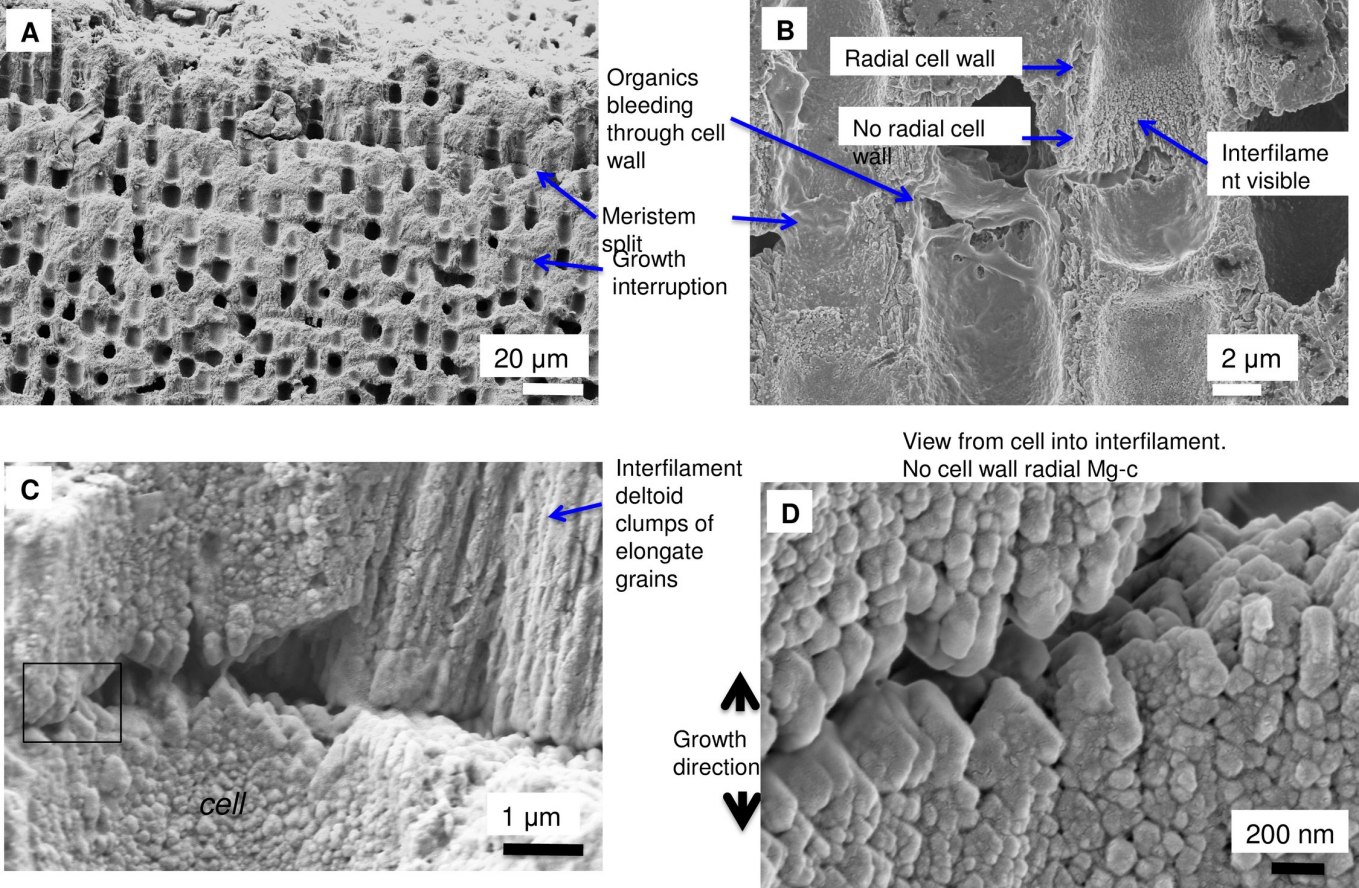
Meristem split in *Clathromorphum compactum* (Gulf of Maine, USA). (A) Overview. Cell layers above the split break off. (B) Absence of radial calcification in cell wall nearest to meristem split, organics leak into interfilament region. (C) Split forms laterally across cell and interfilament. Black box enlarged in D. (D) Growth features mirror both up and down from split.

**Fig 32:**
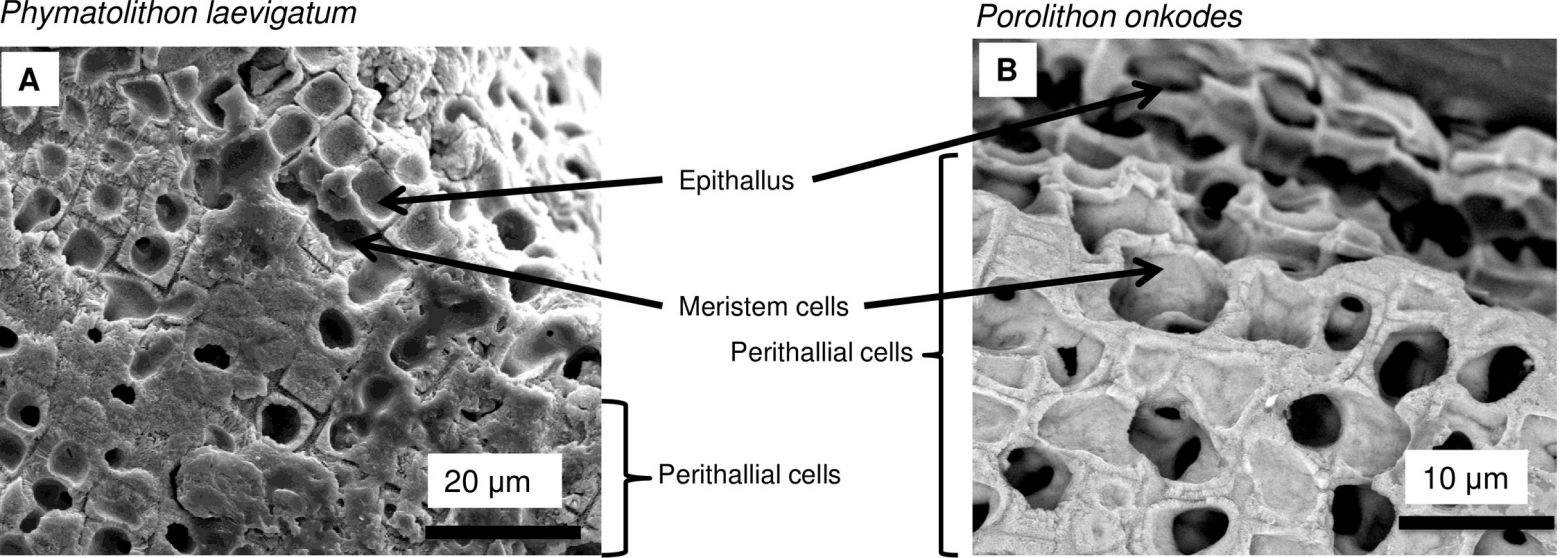
Meristem cell layers without split. (A) *P*. *leavigatum*, (sample from [39]) meristem visible between the change from thinner cell walls of the epithallus to thicker perithallial cell walls. (B) *Porolithon onkodes* (Heron Is. Great Barrier Reef, Australia). The epithallus peels off, but there is no split along the meristem cell layers. Reproduced with permission from [10].

**Fig 33:**
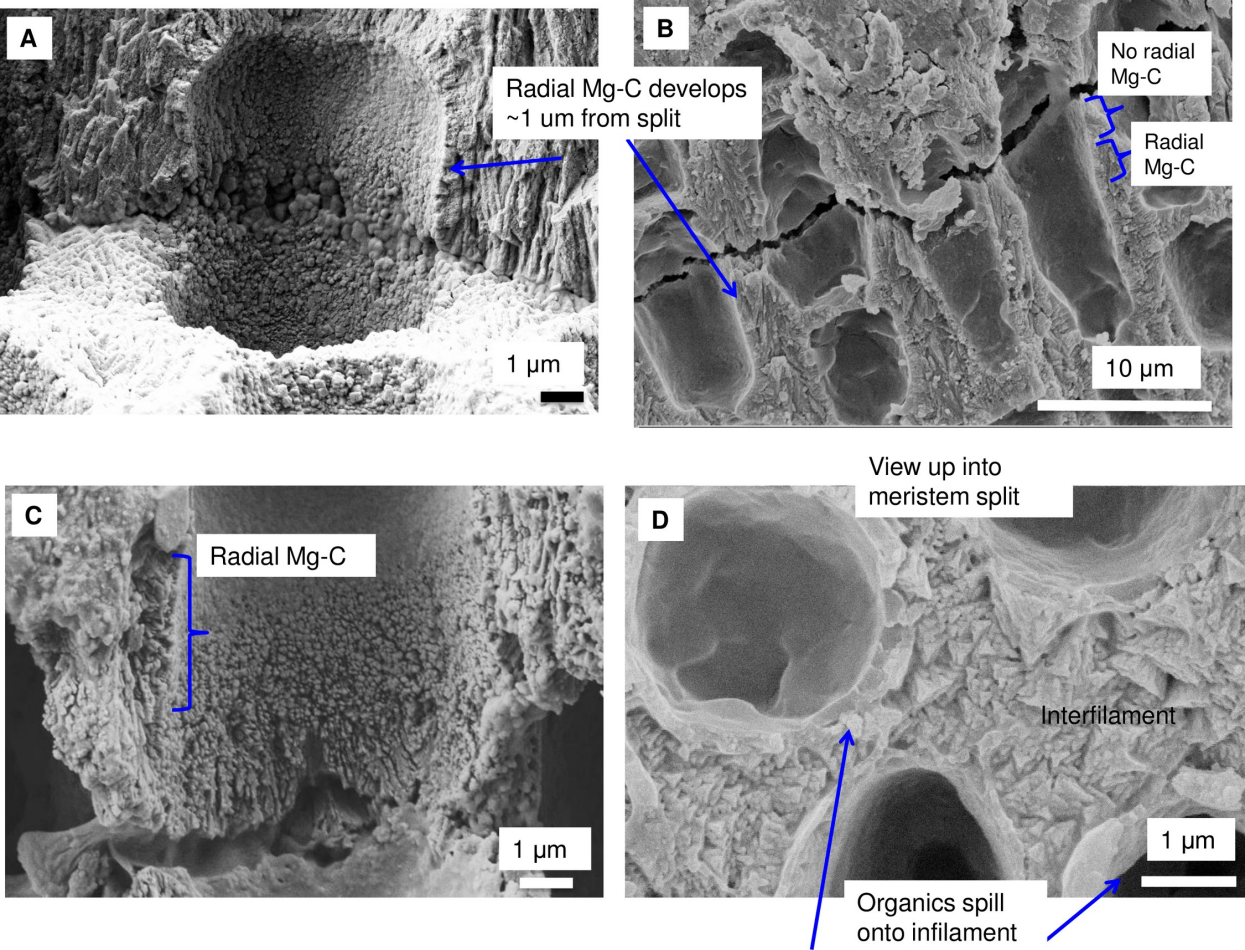
Location and development of radial cell wall in *Clathromorphum*. **(**A, B, C) *C*. *compactum* (Gulf of Maine, USA). Radial calcification present ~1 μm from split. (D) *C*. *nereostratum* (Bering Sea). View up into perithallial cells above meristem split.

**Fig 34:**
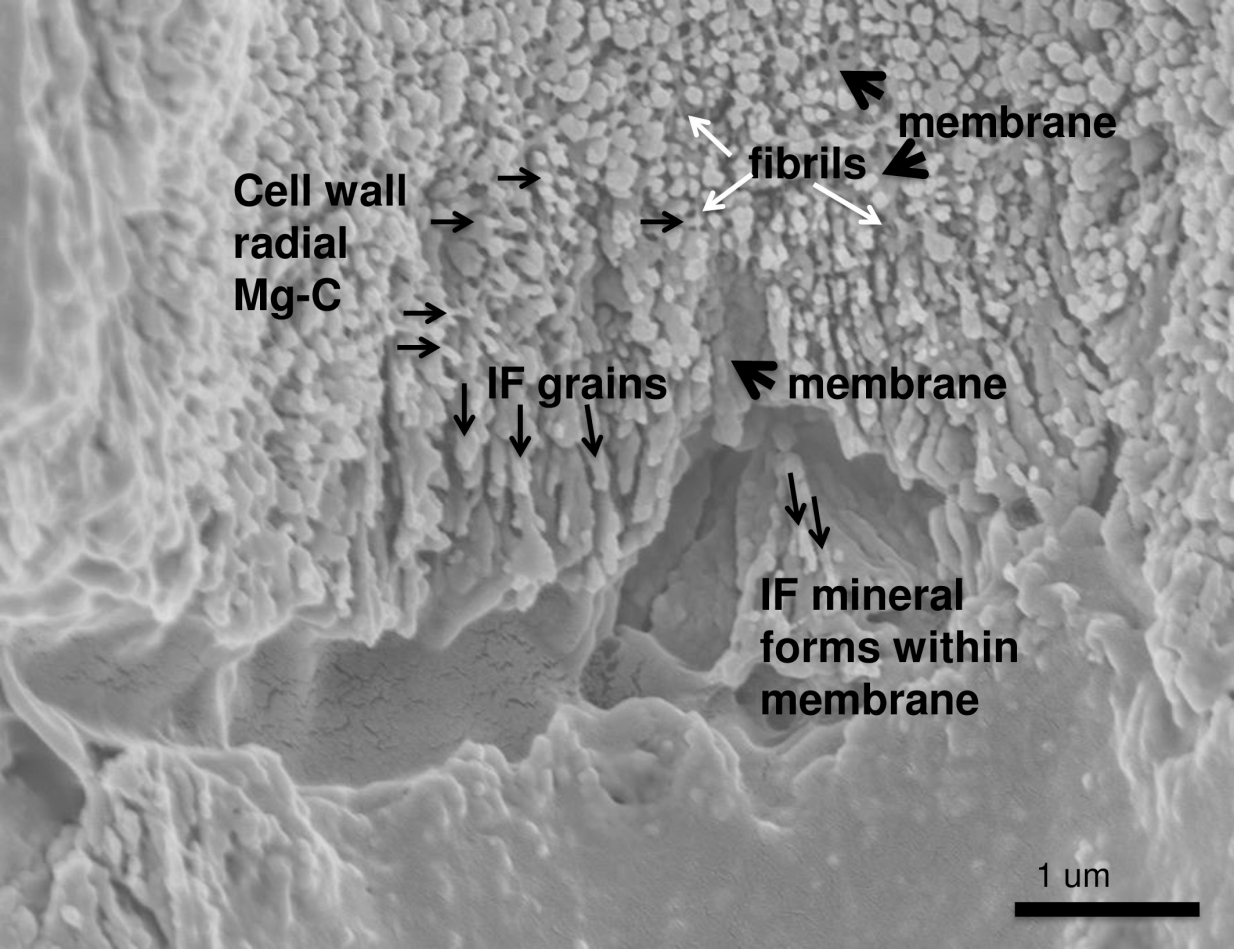
Formation of the interfilament and delayed development of SCW at meristem split. *Clathromorphum compactum*, (Gulf of Maine, USA). At the split there is no calcified material. Organic material leaks through the split into the interfilament area outside of the cell. Calcification commences with the formation of interfilament grains vertically orientated. The perpendicular radial Mg-calcite forms ~1 micron above the split and is longer, further from the split. Fibrils are visible stretching between the cell wall radial Mg-calcite crystals. In this image there is no readily visible PCW carbonate.

Species in the genus *Clathromorphum* genera are unique amongst CCA as they are the only known species that develop a split along the meristem cell layer [14]; it is in this split that cellular growth, calcification and cell division occurs (Fig 31). Other genera of CCA provide cell division in the meristem, but cell elongation growth is progressive (gradual) away from the meristem (Fig 32). For this reason the coralline model (Figs 2 and 3) is adapted (Figs 4 and 5) to account for the unique features present in the *Clathromorphum*. The cell formation outwards (up) and inwards (down) from the *Clathromorphum* meristem split is a mirror image (Fig 33). Both sides show ~ 1 micron of PCW-only above and below the split line (stage 1, Figs 4 and 5) (Fig 34) through which CMF leaks into the interfilament zone (stage 2, Figs 4 and 5) (Figs 33 and 34). The interfilament is initially the bulk of the calcification formed at the split (stage 3, Figs 4 and 5), with minor mineralisation within the PCW (stage 4, Figs 4 and 5) followed by formation of the radial SCW (stage 5, Figs 4 and 5). This thinner cell wall creates a zone of weakness that is the split, resulting in easy removal of the surficial cell layers (epithallial and 1–7 perithallial cells) (Fig 31). The leaking of organic material into the interfilament (Figs 33 and 34) has not been observed for any other coralline algae we have imaged, nor have we found any other examples in published literature. There is no typical PCW carbonate visible prior to the formation of the radial Mg-calcite (Fig 34) and it is possible that there is minimal PCW carbonate present in the side cell walls.

**Fig 35:**
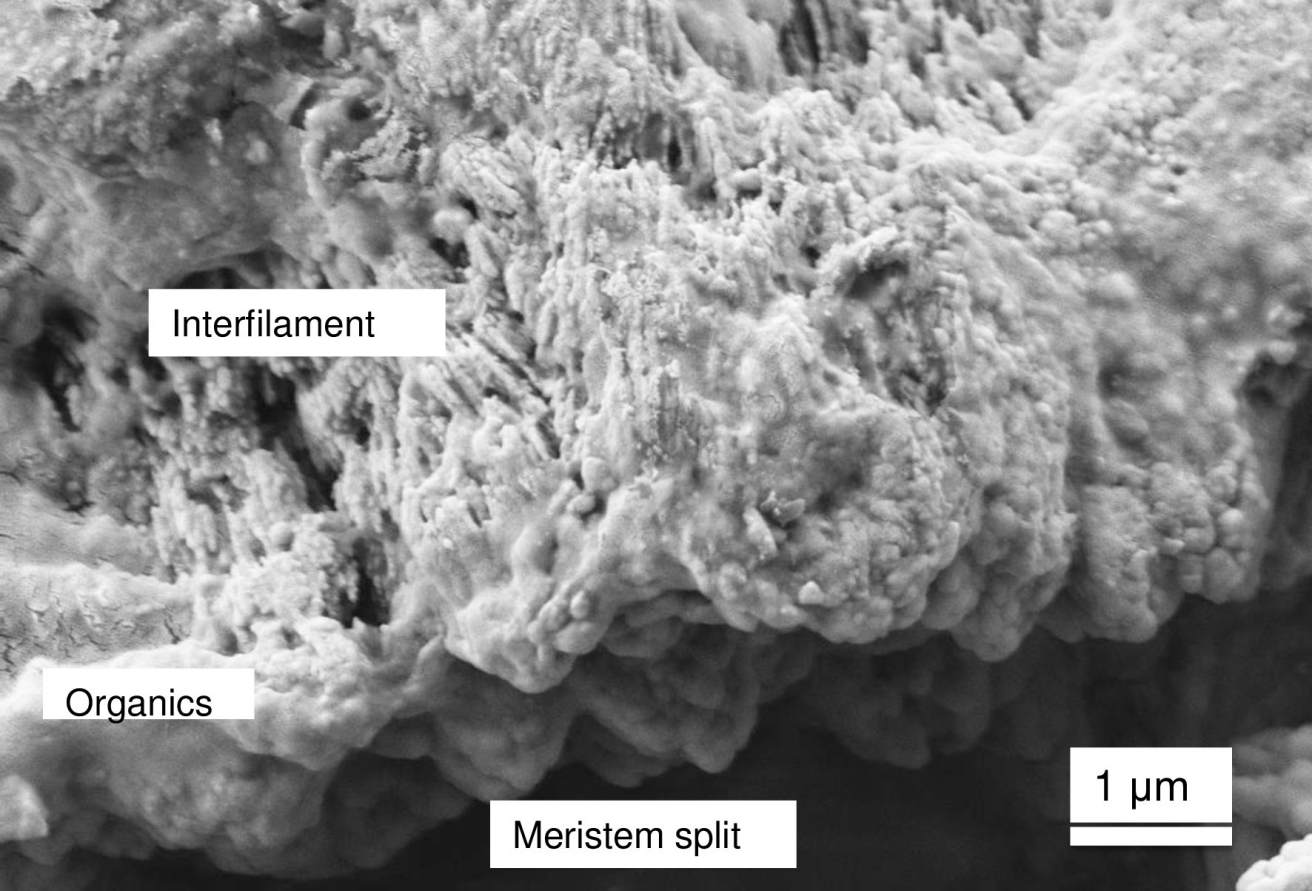
Interfilament Mg-calcite in *Clathromorphum compactum* (Greenland). Carbonate along meristem split is coated with organic material.

**Fig 36:**
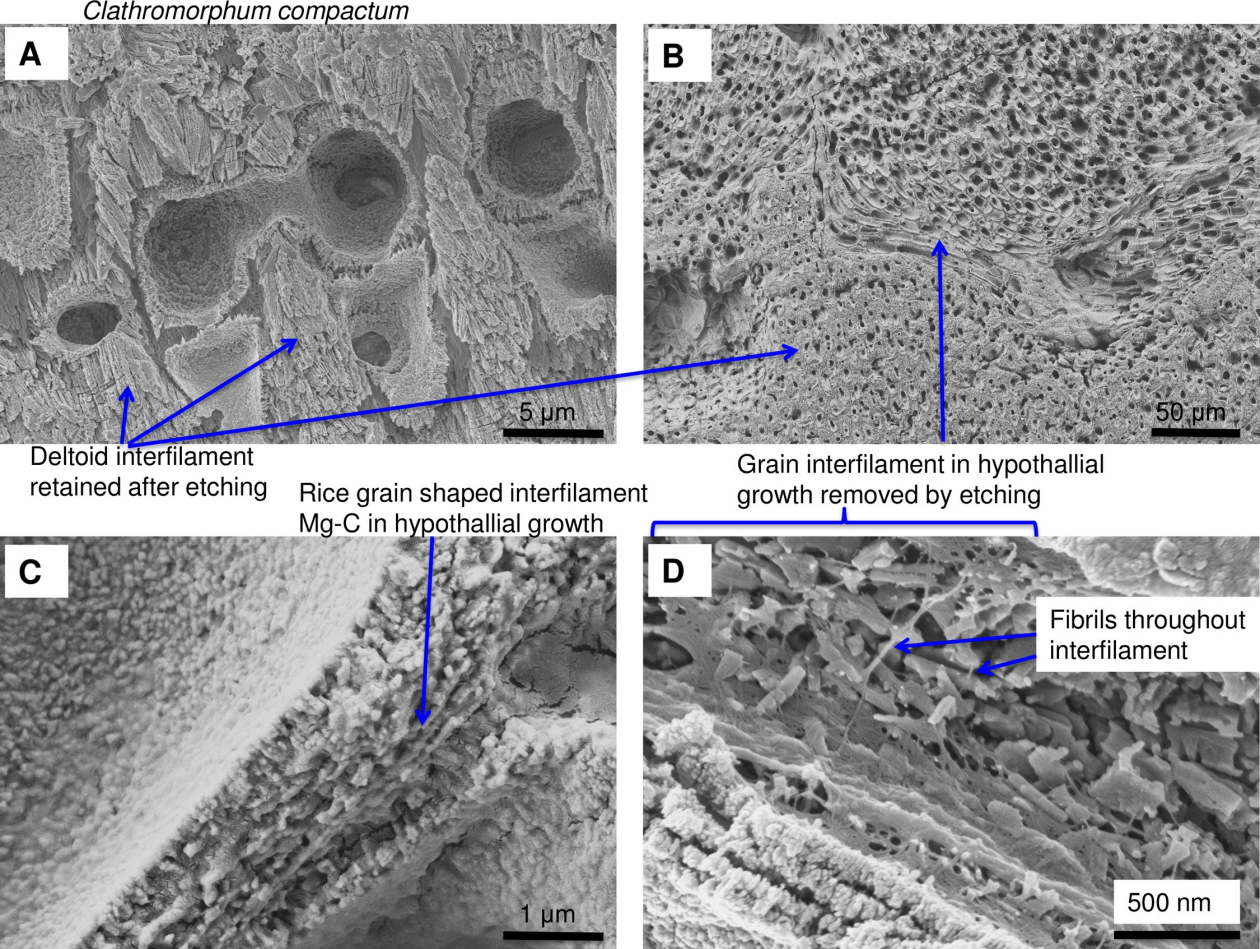
Rice-grain interfilament and response to etching in *Clathromorphum compactum* (Greenland) perithallial and hypothallial growth. (A) Deltoid interfilament is not removed by etching. (B) Rice-grain interfilament in hypothallial is removed by etching. (C) Intact hypothallial interfilament. (D) Close-up from B, showing fibrils throughout the hypothallial interfilament, visible after etching. The *Clathromorphum* hypothallial interfilament is comparable to hypothallial interfilament in other CCA genera.

The interfilament of the *Clathromorphum* sp. is distinctly different from the other genera we analysed. Morphologically, the crystals (~ 2–3 microns long) are ~5–15 times the length of interfilament Mg-calcite grains in other genera. The shape is thin and cylindrical (Figs 27 and 35), in contrast to the rice-grain shape in other genera. Further, in other genera, the interfilament crystals are generally aligned with the nearest flat surface [38,39], whereas in *Clathromorphum* sp. the grains form into a deltoid clump (Figs 27 and 36) with an arrowhead along the meristem split (Fig 35). In our SEM analyses, the etching method that dissolved the interfilament of other genera had no apparent impact on the deltoid interfilament (Fig 36) remaining intact similarly to the radial Mg-calcite. The rice-grained shaped interfilament between the *Clathromorphum* hypothallial cells was removed by the etching, indicating the *Clathromorphum* deltoid interfilament has a degree of extra protection relative to the rice-grain interfilament. Considering that the interfilament responds similarly to the radial Mg-calcite under etching conditions, and the consistency of its morphology, we propose that the deltoid interfilament in the *Clathromorphum* is mineralised CMF (stage 3, Figs 4 and 5), similarly to the radial Mg-calcite in the SCW.

**Fig 37:**
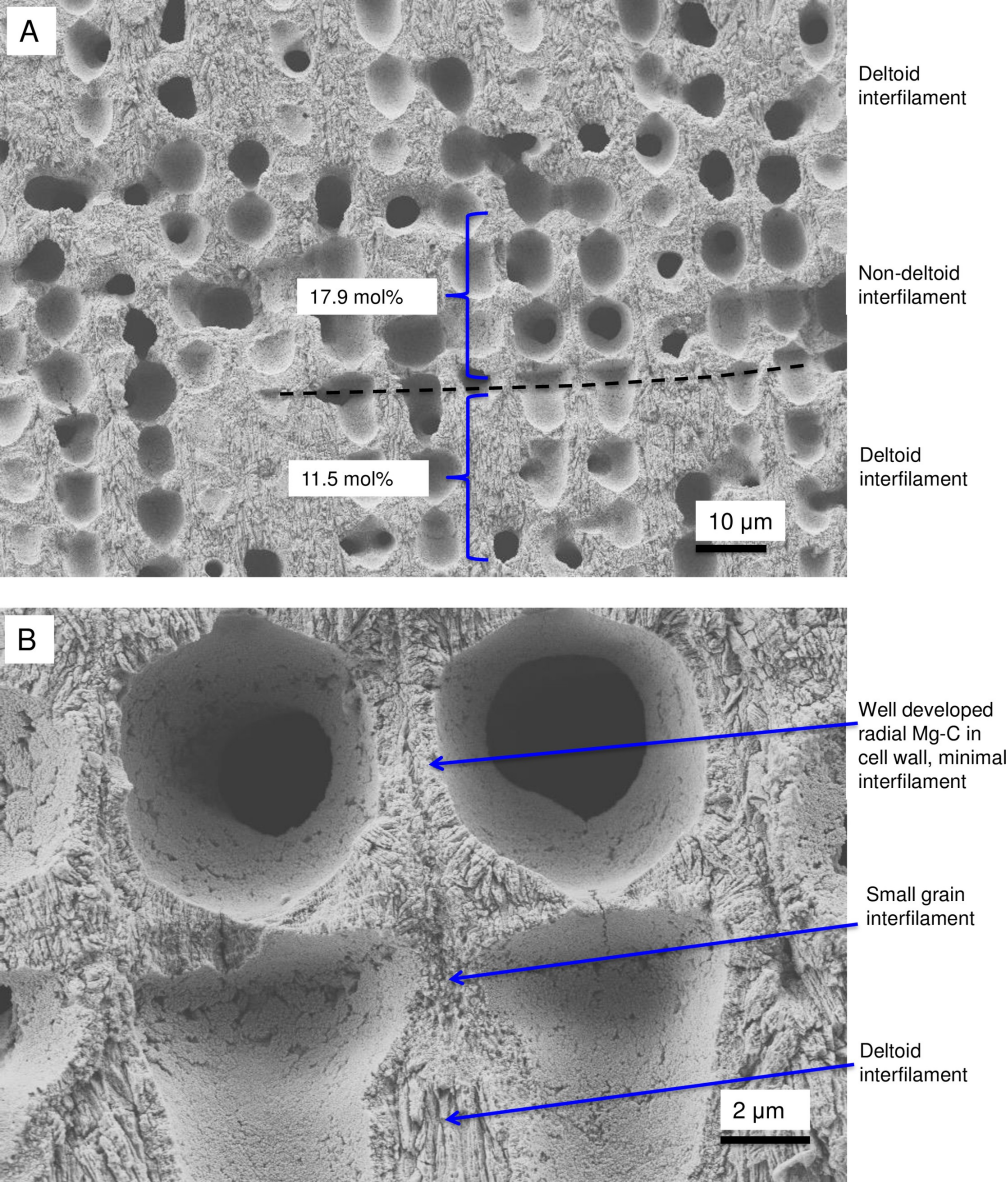
Interfilament in *Clathromorphum circumscriptum*, (Norway). (A) Transition from deltoid to non-deltoid interfilament corresponds with change to higher mol% MgCO_3_. (B) Corresponding with the absence of deltoid interfilament is thicker side cell wall and shorter cell length.

Within discrete parts of *Clathromorphum* crust, there can be a switch from the deltoid interfilament, to minimal rice-grain type interfilament while the cell wall continues to form radial Mg-calcite (Fig 37). This results in a decrease in the total thickness of the wall and interfilament. The Mg-content is higher for the non-deltoid cell wall relative to cell wall with deltoid (17.9 and 11.5 mol% MgCO_3_ respectively) (S2 Table). It is not clear what drives the switch to non-deltoid interfilament. Possibly this is an abrupt response to an environmental change such as resumption of growth of winter period growth cessation [14] or regrowth after shallow wounding. The higher Mg content in the cell walls of the non-deltoid interfilament region cell walls provides support for the possibility that there is an absence of PCW carbonate in cell walls associated with the delayed formation of the SCW.

In other genera we have studied (*Porolithon*, *Phymatolithon*, *Leptophytum*) [10,38,39], the Mg content of the interfilament Mg-calcite is always lower than the cell wall Mg-calcite Mg content. In contrast, the results for the *Clathromorphum* are inconclusive. Mg content for interfilament and SCW were not different for *C*. *circumscriptum*, [Cell wall is 13.8 mol%, (n = 8), interfilament 13.1 mol% (n = 8), p = 0.39] but were different for *C*. *compactum* [interfilament 13.2, mol%, n = 8, SCW 16.1 mol%, n = 8, p = <0.01)] (S2 Table).

#### Possible linear cellulose synthase complex in *Clathromorphum*

**Fig 38:**
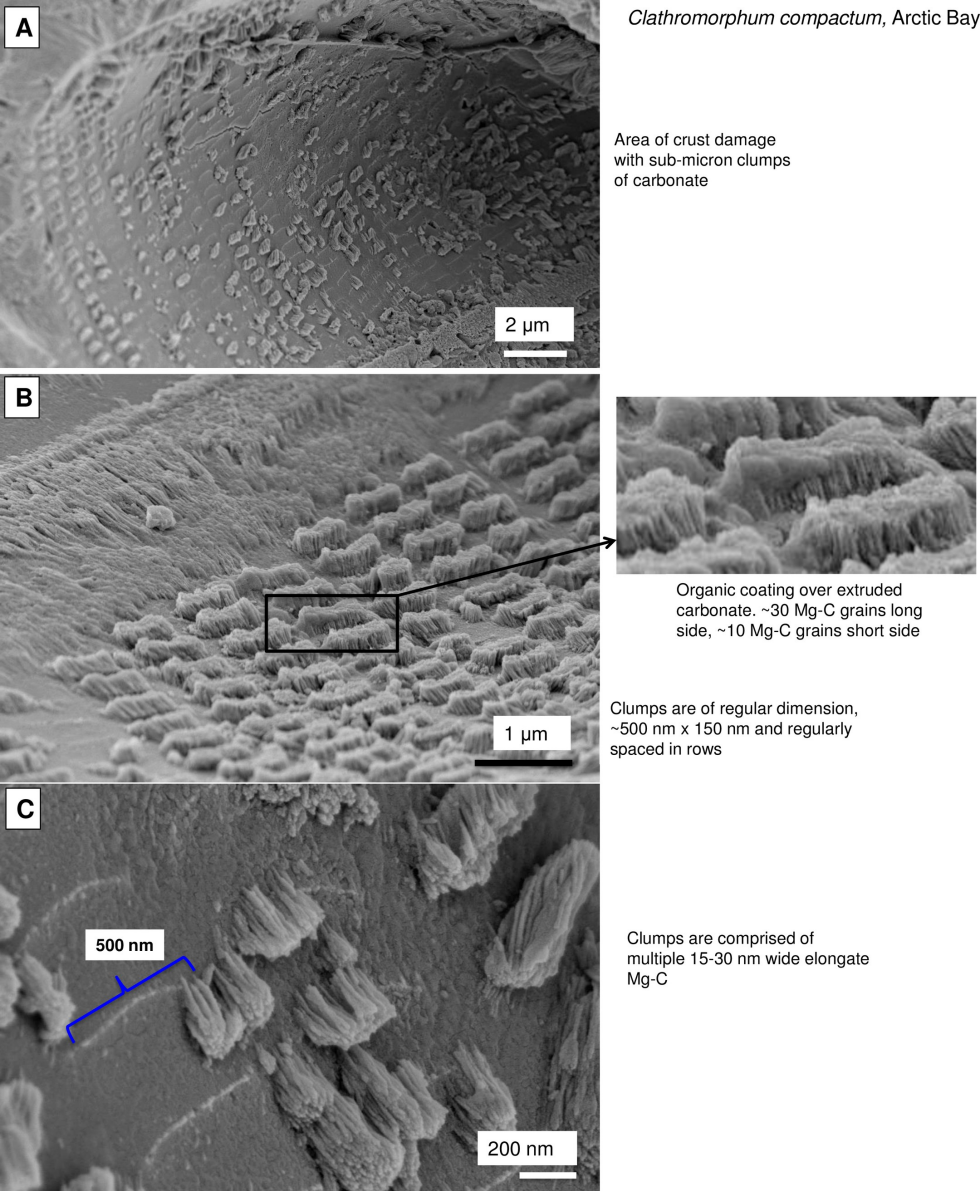
Rows of carbonate clumps within a portion of damaged crust in *Clathromorphum compactum*, (Arctic Bay). The regularity of their size and spacing suggests a strong bio-controlled process. Possibly these are cellulose synthase complexes (or terminal complex) that normally would be active within the cell wall and have reactivated after damage to the crust.

Tsekos [70] proposed that in marine algae, the CSC’s are in linear grids, not the rosette formation found in higher plants. The form of the CSC’s in the corallines was not specifically investigated in this study. However, support for a linear grid formation in the corallines may be found in an area of damage and partial regrowth imaged in a *C*. *compactum* sample (Fig 38). There is a regular grid pattern of rectangular dimensioned carbonate extrusions. These extrusions are clumps of mineralised fibrils of comparable diameter to the radial Mg-calcite and interfilament cylindrical grains. There are ~30 fibrils on the long side and ~10 on the short side. We can find no other explanation for these features, other than they are developed from CSC’s. However, specifically targeted analytical work would be required to confirm this proposition.

#### The change from primary to secondary cell wall

**Fig 39:**
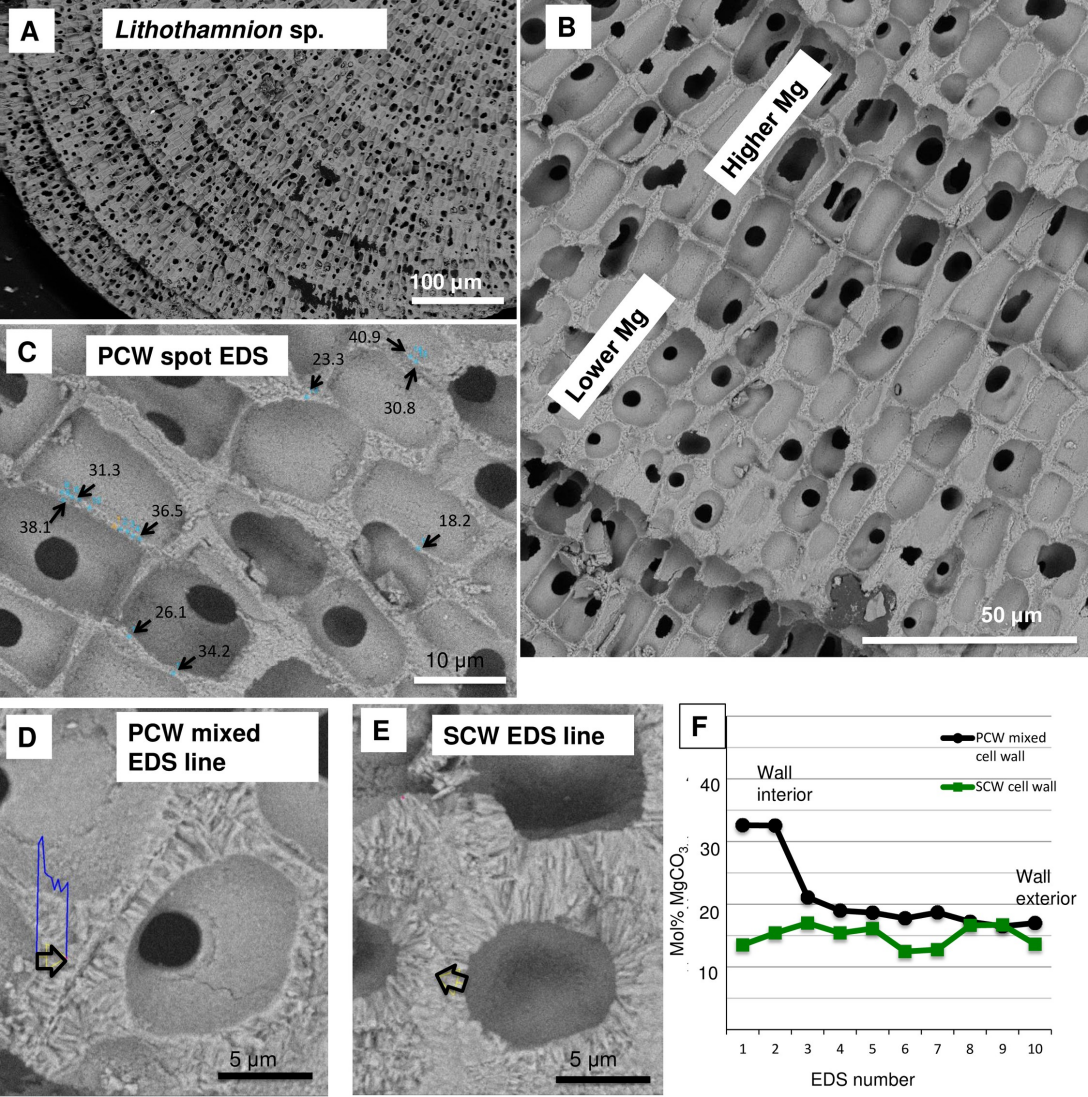
Transition from SCW to PCW cells in *Lithothamnion* sp., and Mg content change (Panama, sample from [26]). (A) Banding. (B) Overview of Mg-content change. (C) EDS spot analyses on PCW-only cell walls. Values range from VHMC (25–36 mol% MgCO_3_) to dolomite composition (>37 mol% MgCO_3_). (D) EDS line (black arrow) across transitioning cell wall. Blue plot is the relative change Mg content by weight percent, mol% values plotted in F. (E) EDS line across perithallial SCW cell wall, mol% values plotted in F. (F) Mol% MgCO_3_ values for the EDS lines in D and E. The interior edge of the transition cell wall has elevated Mg values, comparable to PCW, the remainder of the cell wall with developing radial Mg-calcite has values comparable to the SCW-only perithallial cell wall.

**Fig 40:**
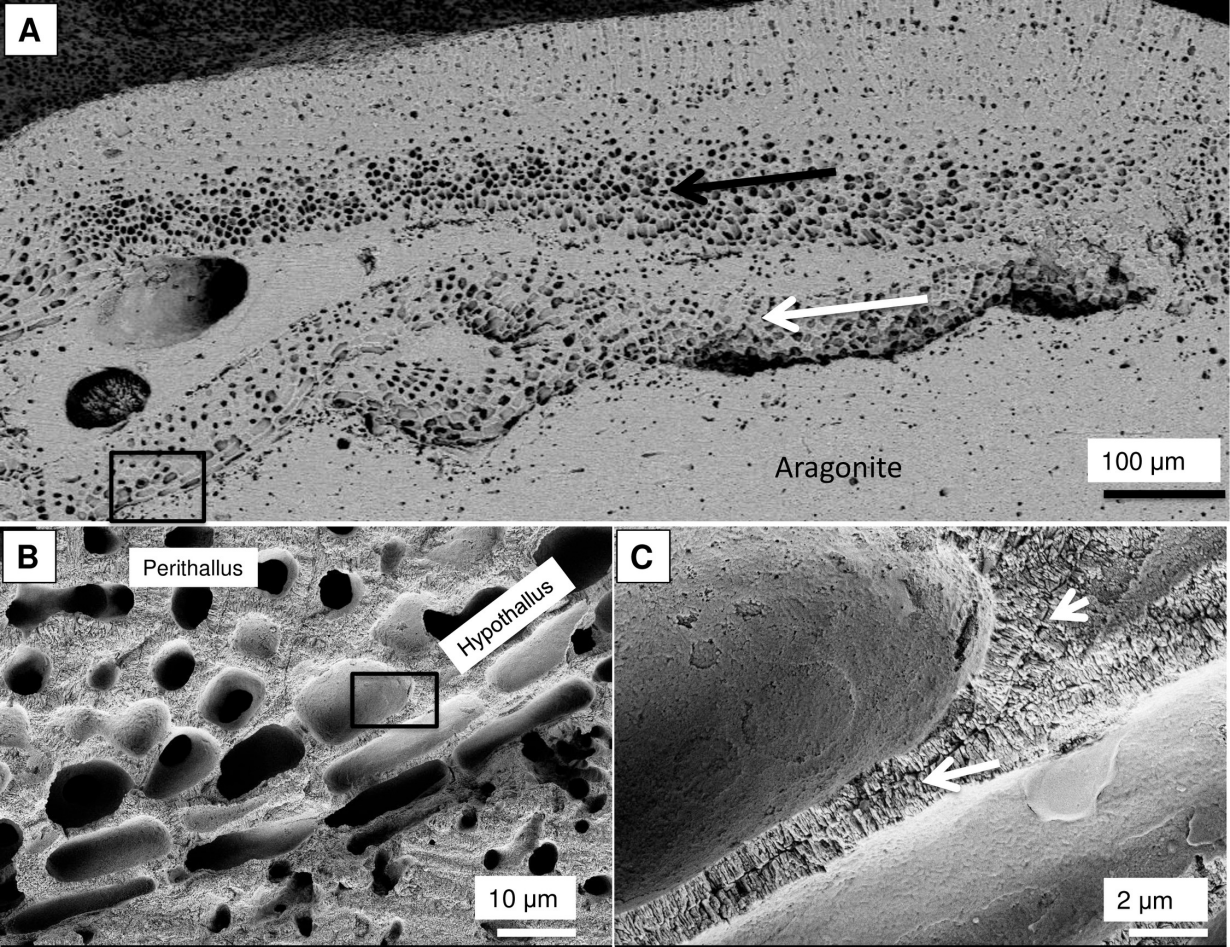
Hypothallial tissue in *Phymatolithon investiens*, (North Norway). (A) Overview (BSE). Basal hypothallus along field of view (white arrow) and into field of view (black arrow). CCA is growing over old CCA crust that has been transformed to aragonite. Black box enlarged in B. (B) Transition from PCW hypothallial cells to perithallial cells with typical radial cell wall and minimal interfilament. Gradual transition from PCW hypothallial to SCW perithallial. This transition is typical for CCA. Black box enlarged in C. (C) Hypothallial cell wall grains appear vertically stacked (white arrows) and second bands of vertical structure walls are forming (white arrowhead). Image republished from Nash and Adey [39] under a CC BY license, with permission from John Wiley and Sons, Copyright 2017.

**Fig 41:**
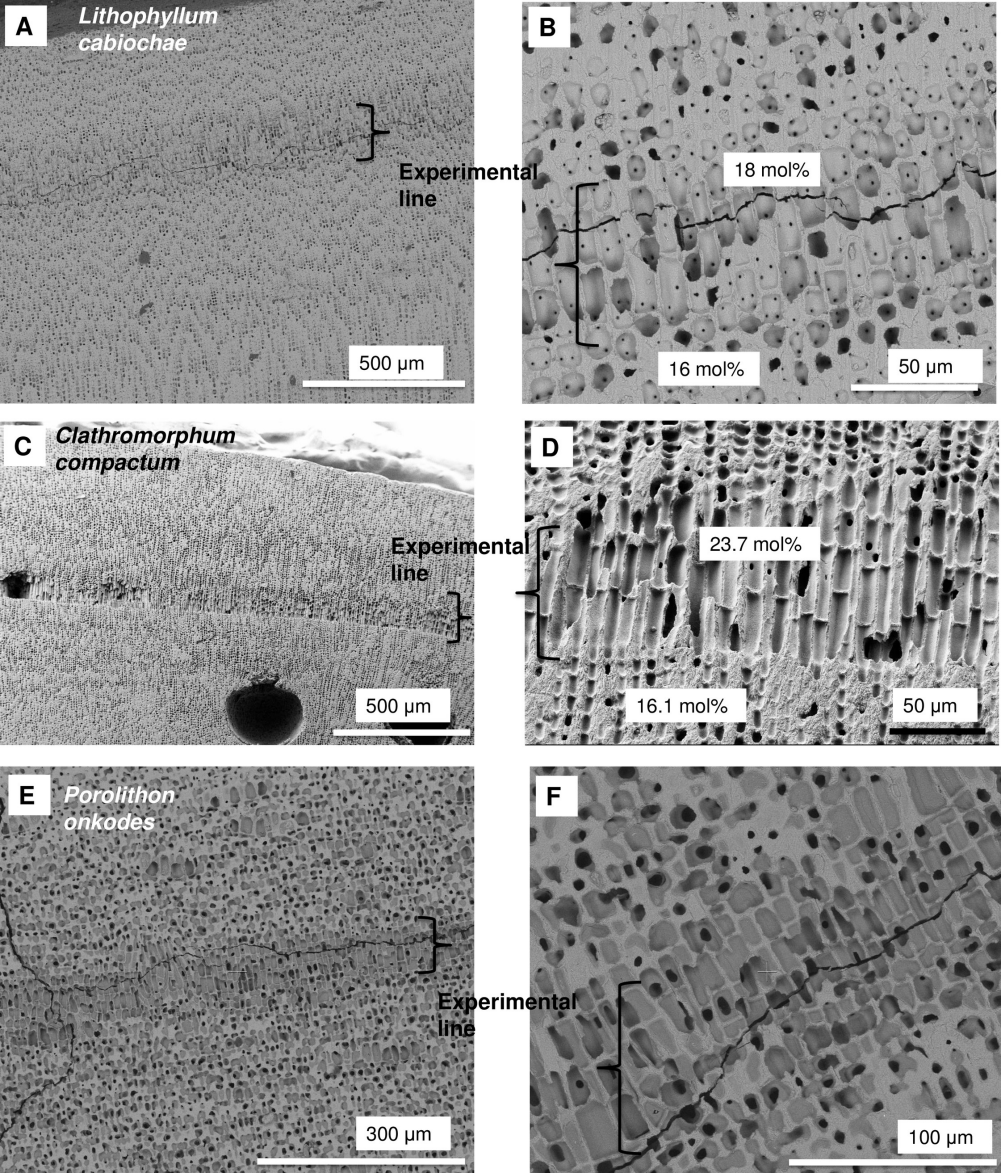
Experimentally induced abrupt shifts from SCW perithallial cells to PCW-only cells. (A, B) *Lithophyllum cabiochae*. Collected from the Mediterranean and placed in aquaria, not stained (sample from [97]). (A) The base of the switch is equivalent to the time of transfer. The switch comprises a row of 3–4 elongate, PCW-only cells followed by a return to perithallial SCW cells. (B) Highest mol% MgCO_3_ of cell walls below and above transfer line from an EDS line transect. (C, D) *Clathromorphum compactum*. Collected from the Gulf of Maine and placed in aquaria, not stained (supplied by J Halfer, University of Toronto). A row of 2–3 elongate, PCW-only cells. The return to cell structure comparable to pre-transfer takes ~10 cells. Average mol% MgCO_3_ of cell walls below and above transfer line (S2 Table). (E, D) *Porolithon onkodes* collected from Heron Island, Great Barrier Reef, transferred to aquaria, embedded in resin and returned to the reef slope within 7 days (sample from [33]). This particular sample was recollected every three months over the following 15 months, kept in aquaria for ~7 days, then returned to the reef. The elongate cell row is only present the first time it was collected. Higher Mg content in elongate cells indicated by darker grey shades. Values ranged up to 80 mol% MgCO_3_.

**Fig 42:**
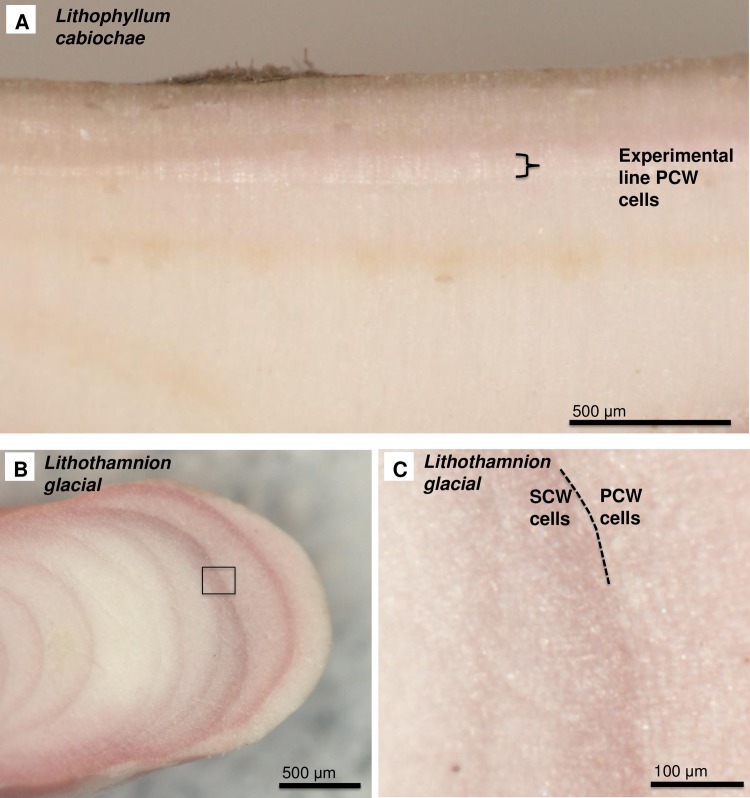
Pigment in SCW and PCW. (A) Experimental crust of *Lithophyllum cabiochae*, (Mediterranean, sample from [97]). Where the crust switches to elongate cells photosynthetic pigment is mostly absent. (B, C) Rhodolith *Lithothamnion glaciale*, (Scotland, sample from [18]). Pigment concentrates with increased SCW-only cells at the edge of the bands. The abrupt shift to PCW-only cells corresponds with reduction in pigment, which gradually builds up again as the cells shift back to perithallial SCW cells.

Within a CCA, the change from cells with SCW to PCW-only cells is always seen as abrupt switch with a clear change in cell wall structure across a < 1 micron length (Figs 6C, 6E and 39). In contrast, a shift from the SCW-only to PCW is usually gradual (Figs 13A and 40). The SCW thickens over 2–10 cells before forming the complete SCW. The abrupt switch to PCW after a wounding event can be explained by the physical removal of the shallow SCW perithallial cells, followed by a switch of the remaining cell to repair mode, hence being only PCW. However, there are examples where there have been abrupt switches without wounding. In three CCA used in growth experiments in previously published studies [33,97] and an unpublished study (S3 Table), we identified bands of elongate PCW-only cells, with elevated Mg, that formed immediately after the time of collection (Fig 41) (S2 Table). The three samples containing this band were three different genera (*Lithophyllum*, *Clathromorphum*, *Porolithon*), collected from three very different environments, (Mediterranean, Gulf of Maine, Great Barrier Reef) and all subject to different treatments (*Lithophyllum cabiochae*—no staining, transfer to higher CO_2_ and temperature treatment; *Clathromorphum compactum*—staining which did not take and transfer to aquaria; *P*. *onkodes*—staining and embedding in resin, 7 days in total in aquaria then returned to the reef site). The only common element was removal from the natural environment and transfer to aquaria. It is not known what drives this growth response to changing culturing conditions. In *L*. *cabiochae* there is also an abrupt absence of photosynthetic pigments in this transfer band (Fig 42).

### Banding in rhodoliths

**Fig 43:**
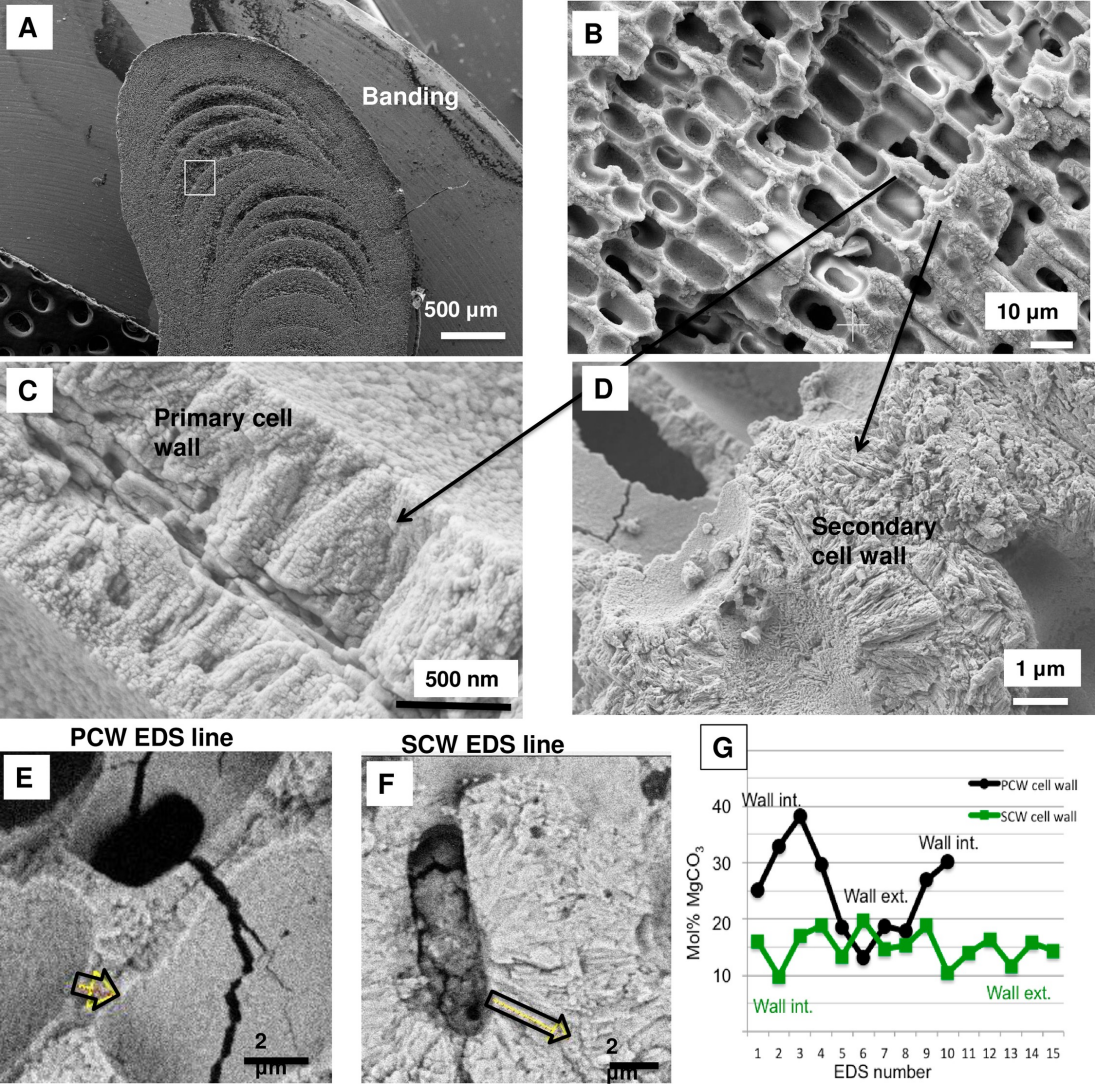
Transition from SCW to PCW cells in *Lithothamnion glaciale*, and Mg-content change (Scotland [18]). (A) Banding in *L*. *glaciale*, white box enlarged in B. (B) Abrupt switch from perithallial cells with SCW to hypothallial-style cells with PCW-only. (C, E) PCW densely calcified, no clear radial calcite structure. (D, F) SCW with radial Mg-calcite. (E, F) SEM-EDS transects across the cell walls (black arrows). (G) EDS mol% MgCO_3_ measurements across the cell walls. The PCW has values up to dolomite composition, with lower values equivalent to SCW radial Mg-calcite. Wall int.: interior edge of cell wall. Wall ext.: exterior edge of cell wall.

The banding of low to high-density cells, as found in many branching rhodoliths [18,26,98] is formed by a periodic abrupt switch from SCW cells to PCW-only cells and the gradual shift back to SCW cells (Figs 39 and 43). This switch can take place across a cell, before the cell has been fully formed, suggesting this change is an abrupt response to an external event. The PCW cells have the highest total Mg-content. Over ~5–10 cells, as the walls thicken with the formation of SCW there is an associated decline in average Mg-content (Fig 39).

**Fig 44:**
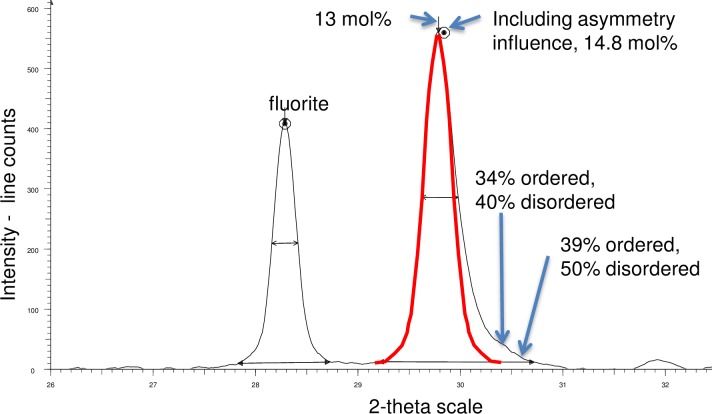
XRD pattern for *Lithothamnion glaciale*, (Scotland, sample from [18]). Black line is *L*. *glaciale*. Red line is symmetrical scan made by mirroring the left side of the Mg-calcite peak. Area between the red and black lines is the asymmetry attributable to higher Mg phases. Based on this and the EDS, the PCW mineral ranges from 30–50 mol%, assuming this is disordered dolomite: % ordered: mol% calculated if using calibration for ordered dolomite. % disordered: mol% calculated if using calibration for disordered dolomite (Methods).

The Mg content for the higher Mg carbonates in the PCW was determined using X-ray diffraction. The *Lithothamnion glaciale* (sample from Scotland) Mg-calcite has 13 mol% MgCO_3_ as determined from X-ray diffraction (XRD) of a bulk sample (Fig 44). However, on the XRD Mg-calcite peak there is distinct asymmetry toward higher Mg extending over the position for 40–50 mol% for disordered dolomite (34–39 mol% for ordered dolomite). SEM-EDS transects across the cell wall return ranges of 25–40 mol% MgCO_3_ (Fig 43) noting these values probably do not capture the highest actual Mg-content due to contribution from surrounding lower Mg values. These range of values are outside of what is considered Mg-calcite, and in the range for VHMC and approaching dolomite values (> 37 mol% MgCO_3_). Photosynthetic pigments increase with the increasing amount of SCW and there is an abrupt cessation of pigments aligned to the change to PCW-only cells (Fig 42). This feature of higher Mg to dolomite composition is also present in *Lithothamnion* sp. from Panama (Fig 39).

### Summary of calcification features

A summary of the calcification features in the coralline algae analysed is presented in Table 2.

**Table 2:** Summary of calcification features.

	Most CCA (non-geniculate)	*Clathromorphum* sp.	Articulated (geniculate)	Epiphytic CCA	Rhodoliths
**Primary Cell Wall–PCW calcification**	Present	Present	Present	Inconsistently present	Present
**Secondary Cell Wall- SCW calcification**	Present	Present but delayed formation	Present	Not observed	Present
**Interfilament calcification**	Present Rice grain shaped	Present Mainly deltoid clumps. Minor rice grain shaped	Minimally present	Present, may be elongated grains	Present Rice-grain shaped
**Interfilament Mg content relative to cell walls**	IF Mg < CW Mg	IF Mg </ = CW Mg	IF Mg < CW Mg	NM	IF Mg < CW Mg

NM- not measured.

## Discussion

### Comparison of coralline algal cell walls with other plants

Our study shows that the coralline PCW-only cells form when rapid growth and flexible shape is required as for wound repair, hypothallial growth and central medullary cells articulate coralline algae. In contrast, SCW develops in mature cell growth where consistent shape and strength is required as in the perithallial cells of CCA. This distinction is similar to higher plants, where the PCW consists of the flexible polysaccharides cellulose, hemicellulose and pectin; the SCW also has rigid lignin. Lignin and the formation of a non-calcified secondary cell wall have been found in the decalcified genicula of the articulated geniculate coralline, *Calliarthron heilosporioides* [75]. It is tempting to equate SCW calcification in CCA to the lignin of woody plants. Indeed, it does appear that calcification allows the vertical accretion of the CCA, similarly to the strengthening by lignin enabling load-bearing wood.

### Insights from cellulose biomineralisation experiments

Experimental work using cellulose to induce mineral formation provides support for our proposal that biomineralisation proceeds via the soaking of cellulose in seawater, together with the organism-produced organic compounds. Abundant calcium-phosphate formed when cellulose was pre-soaked in calcium-rich fluid then soaked in compounds found in mammalian bone [91]. Mg-calcite formed when coralline algal organic extracts were added [99], or the concentration of the cellulose derivative was increased [100]. It is also conceivable that the organism fluids are not required for carbonate induction where cellulose is present. Carbonate minerals have formed in beaker conditions where the wood or cellulose derivative has been simply soaked in or seawater-similar [100] or calcium-rich fluids [101]. Borowitzka [43] observed that fleshy algae have a greater number of complex organic compounds than calcifying algae (including non-corallines). Borowitzka proposed that within these extra compounds there may be a carbonate inhibitor, and that but for this inhibitor, calcification could proceed in all marine algae. Thus, although we propose in our model that organic compounds are part of the process, it is possible that the coralline cellulose soaking in seawater, in semi-closed conditions and without a calcification inhibitor, is sufficient for carbonate to form.

### Is calcification controlled or induced?

No model of calcification would be complete without consideration of whether the mineral formation is controlled or induced. This is important because it aids our predictions of how external factors may or may not impact calcification. We specifically clarify here that for this consideration, we are differentiating between the production of the organic cell wall structure, which is a metabolically controlled process, and the subsequent formation of Mg-calcite. While it could be argued that because formation of the organic structure is biologically controlled, and, Mg-calcite formation is dependent those sites for a nucleating substrate, then, therefore Mg-calcite formation is biologically controlled. We specifically address this argument by looking to the characteristics currently accepted [102,103] as features to define controlled mineralisation as compared to biologically induced mineralisation. Before progressing, we clarify the definitions we rely upon in this discussion as they pertain to coralline algae. **Controlled**: a calcification outcome that is not a byproduct of another process, but where that other process exists solely and specifically for the purpose of driving calcification. **Induced**: a calcification outcome that is a byproduct of a process that is not occurring for the purpose of that outcome. **Site of calcification**: the exact point on/in a substrate where mineral nucleation takes place. **Calcification compartment**: the larger space, cell wall or interfilament, containing the sites of calcification. **Organism fluid**: the matrix fluids produced by the coralline and present in the cell wall and interfilament. **Calcifying fluid**: the mix of organism fluid and seawater from which minerals form. **Ion transport**: an active transport mechanism to transport charged ions across an otherwise impermeable barrier.

#### Consideration of features characteristic of controlled biomineralisation

The defining features of controlled biomineralisation are; mineralisation in an isolated compartment, a specialised three-dimensional macromolecule organic matrix for mineral formation and the requirement for active pumping of cations from the cell to the site of mineral formation [102]. These are considered in turn below. Our key assumptions underpinning each conclusion are highlighted.

**Isolated compartment** To be completely isolated, the compartment needs to be bounded by impermeable barrier (typically a membrane) where active pumping is required for ion transport through the barrier. This is in contrast to a compartment being semi isolated, as is the well-known case of calcification in *Halimeda* [42]. In the corallines, neither the interfilament nor the cell walls are externally bounded by impermeable membranes. The interfilament opens to the surface of the alga and there can be visible gaps at the surface where the interfilament is exposed [14]. The cell wall of plants is bounded internally by the phospholipid plasmalemma, an impermeable membrane. However, the external perimeter of the cell wall is a fibrillar mesh [77]. While seawater movement into the coralline cell wall will be slowed, providing a semi-closed system, the mesh is not an impermeable barrier that requires diffusion or active pumping to transport ions across. Based on the observations within this study and associated assumptions, calcification in the coralline algae does not meet this defining feature of an isolated compartment. **Key assumption- that the observed mesh of fibrils at the external perimeter of the cell wall is not impermeable to charged ions.**

**Specialised three-dimensional macromolecule organic matrix for mineral formation** In the context of biomineralisation, this criterion is further clarified as the structures and compositions of these organic frameworks that are genetically programmed to perform essential regulating and/or organising functions that will result in the formation of composite biominerals [102]. This typically includes proteins [53,104,105] with a high proportion of acidic amino acids and phosphorylated groups. There is as yet no genetic analysis that we are aware of, that has specifically quantified genes involved in calcification in the coralline algae. However, none of the anatomical features identified in the coralline algae are unique to coralline algae. The interfilament (middle lamella) is common to all plants and fleshy algae as is the PCW and SCW. We have assumed the radial calcite is mineralisation of cellulose and that cellulose in coralline algae is comparable in composition and production to cellulose in fleshy algae and plants. Thus, even the distinctive radial calcite forms on a non-specialised organic matrix. **Key assumption- that the organics mineralised for the radial Mg-calcite are not substantially different from plant cellulose.**

**Active ion transport of cations from the cell to the site of mineral formation** Active ion transport is only necessary to move charged ions across an impermeable membrane, such as from the ambient seawater, across the plasmalemma into the cell vacuole for metabolic processes. Because there are no impermeable membranes separating the interfilament and cell wall sites of calcification from seawater and the contained ions, seawater can freely exchange throughout the calcification compartments. The depth of penetration of seawater by this exchange is demonstrated by the rapid incorporation of stains, e.g. alizarin red or calcein, with calcofluor white penetrating 100’s of microns into CCA *P*. *onkodes* in less than 10 minutes [106]. As there is not an impermeable membrane separating the sites of calcification from their ion source, active ion transport is not required. Therefore, the coralline algae do not meet this criterion. **Key assumption- that the observed mesh of fibrils at the external perimeter of the cell wall is not impermeable.**

**Consideration of features characteristic of bio-induced calcification** Characteristic features indicating bio-induced mineralisation include; variations in structure and particle size, cell surfaces often acting as nucleating substrates and the biology has little control over the mineral type [102]. We add another two criteria; absence of control over calcifying fluid composition and no dependence on elevated pH for mineral nucleation. These are considered in turn below.

**Variations in structure and particle size** Induced biominerals typically show great variation in external morphology, water content, trace/minor element compositions, structure and particle size [102]. In the corallines the three carbonates present (the interfilament, PCW and radial) differ in shape, particle size and elemental composition (Figs 6–37). The PCW shows the greatest variability in size and structure from being poorly calcified to densely calcified, with irregular grain shapes to plate-like, even being at times not present. The interfilament is generally rice-grain shaped but this can vary, along with the amount of interfilament carbonate present. The radial carbonate is the most consistent of the three, being consistent within and across species. When compared to the precise and consistent controlled mineral formations of mollusk nacre [107] and coccolith plates [108] the mineral structures and particle sizes exhibit great heterogeneity. The coralline algae meet this criterion. **Key observation- minerals in CCA are variable in structure and size.**

**Cell surfaces often acting as nucleating substrates** Based on the images presented in Figs 6–27, the plasmalemma membrane itself does not appear to act as a nucleating substrate for the cell wall carbonate. However, the cell wall organics do act as substrates for both the PCW and radial carbonate. The external surface of the cell wall appears to act as a nucleating substrate for interfilament-edge carbonate, but the interfilament grains themselves do not appear to nucleate on the cell wall surface (Figs 17, 20 and 24). In the coralline algae, organic surfaces within the cell wall act as nucleating substrates. This provides support for the conclusion of biologically induced mineralisation. **Key observation- Mg-calcite forms on surfaces.**

**Biology has little control over the mineral type** This is an interesting criterion for biologically induced calcification, and possibly misleading. Biologically induced calcification can form the same type of mineral within all species in the genera, for example, aragonite is always formed in *Halimeda* spp. Clearly the organic environment produced and controlled by the organism influences the type of mineral that could form, should the micro-environmental conditions induce mineral nucleation. There are many examples of marine organisms, which are accepted to have controlled calcification, able to produce both aragonite and calcite (gastropods- [109]) and specific proteins control the switch to aragonite (molluscs- [110]). The demonstration of multiple types of Mg minerals found across micron-scale areas within corallines is however, suggestive of a lack of control on the mineral type. While the suite of carbonate minerals found in CCA, both tropical and Arctic, suggest the corallines meet this criteria for biologically-induced calcification, we question the robustness of this criterion as a characteristic feature. **Key observation- multiple types of Mg-carbonates present within the cell wall. Key assumption- that there are no organics produced by the coralline specifically *for the purpose of* forming Mg-calcite.**

**Absence of control over calcification fluid** We have proposed that calcification in coralline algae is dependent on the mixed matrix fluid, i.e. a calcification fluid that is the combination of the organism fluid with seawater. As the seawater penetration cannot be controlled because the interfilament and external perimeters of the cell wall are porous, it follows that the proportions of seawater and organism fluid in the final mixed matrix fluid cannot be controlled. Thus, while the organism-produced fluid is a result of controlled metabolic activities, the final mixed matrix fluid, i.e. calcifying fluid, is not controlled. This provides further support for calcification being induced, rather than controlled. **Key assumption- that the observed mesh of fibrils at the external perimeter of the cell wall is not impermeable and that calcification is dependent on the mixed matrix fluid.**

**Mineral nucleation not dependent on organism-driven elevated pH** Firstly, we define elevated pH as pH above ambient seawater pH. Recent research has demonstrated pH is elevated above ambient seawater in the calcification fluid of *Neogoniolithon* [40,111], *Sporolithon* and *Amphiroa* [40], and *Clathromorphum* [31]. These findings provide support for elevated pH, however, the lowest reconstructed pH was 8.15 (*Amphiroa*) and that is equivalent to ambient pH in many marine environments [112]. Mg-calcite can form in semi-closed spaces (dead cells and interfilament) in parts of CCA crust that has been damaged where seawater can penetrate, or at the base of the crust where the organism is no longer metabolically active (images in [113]). Beaker experiments using organic extracts from corallines have formed Mg-calcite without elevated pH [99]. A range of Mg-calcite compositions were formed at starting pH ~7.7–7.9 [100] using cellulose and agar extracts. Calcification experiments on dead *Amphiroa* found that calcification continued in dead skeleton, in the dark and at low pH 7.2 [45]. More recently, calcification-inducing proteins extracted from coral, were able to induce aragonite formation at pH of 8.2 and 7.6 [104] indicating that high pH is not necessarily required for mineral formation. These studies indicate that calcium carbonate is not always dependent on elevated pH to nucleate. Thus caution must be exercised when drawing a causal relationship from the determination of elevated pH in coralline carbonate to concluding that elevated pH is a requirement for mineral nucleation.

While it is well established that elevated pH will increase rates of precipitation, and pH in the coralline cell walls and boundary layer will be elevated by photosynthesis and possibly other metabolic processes [37,40] thus increasing rates of precipitation, there is no evidence to suggest that calcification cannot proceed without elevated pH. Indeed, the continuation of calcification in dark conditions [14,37] indicates that this process is at the least not dependent on photosynthetic or light driven pH elevating processes, the only pH elevating-processes known for the corallines at this time. Even with elevated pH driving faster rates of calcification, the total amount of carbonate will be limited by the amount of cell wall and interfilament space available. As evidence indicates that Mg-calcite can form in solution below ambient pH and there is no evidence thus far demonstrating that elevated pH is required for calcification in corallines, this suggests that there is no requirement for the organism to actively elevate pH for the purpose of calcification. Therefore, this criterion is supported. **Key assumption- that mineral nucleation can proceed within the coralline at the same solution pH levels demonstrated experimentally.**

**Support for biologically induced calcification** The main criteria for induced calcification in coralline algae are either proven or supported, while there is little support for criteria defining controlled calcification. Thus it appears that calcification in coralline algae is probably an induced process, not specifically controlled by the organism. Clearly the organic structures of the coralline and its internal processes enable calcification, but *enable* is not the same as *for the purpose of* that is required to meet the definition of controlled calcification. It is this difference that we have explored with this discussion and found that this purpose has not been demonstrated. It is important to note our key assumptions underpinning our conclusion for induced calcification and that should any of these be proven in the future to be incorrect then our conclusion of induced would need to be revisited. At this time we are not aware of any data that would suggest our key assumptions are wrong.

### Rates of calcification and consideration of active ion transport

Although we suggest that calcification is not dependent on active ion transport to the site of calcification, active transport of ions for metabolic activity must have an influence on calcification rates. For coralline algae, active HCO_3_^-^ transporters (CO_2_ concentrating mechanisms) are used to move DIC across the plasmalemma to elevate CO_2_ at the site of Rubisco, as evidenced by organic δ^13^C values > -29 [36,114,115]. It is likely that a proportion of this respired CO_2_ is incorporated into the carbonate. Continued growth and calcification of coralline algae in dark conditions [14,37] and without photosynthesis (*Kvaleya epilaeve* Adey & Sperapani) [46] indicate that calcification is not limited by photosynthesis, other than for the initial provision of stored energy required to continue growth. Active pumping for metabolic purposes will likely increase the rates of seawater exchange as ions are drawn from the solute in the cell wall and utilized in growth processes and this will likely increase the rate of calcification.

### Impacts of warming on *Clathromorphum* species

Understanding the differences in calcification that are associated with the meristem split in *Clathromorphum* is important as calcification may respond quite differently to other coralline algae under warming temperatures. Because of the meristem split, penetration of the ambient seawater to the site of calcification will likely be greater than for CCA without this split. Some of the compounds in red algal cells are water-soluble [99,116] and solubility increases with elevated temperatures [117]. The distribution of *Clathromorphum* is restricted to cooler waters of the Sub-arctic and adjacent Boreal regions (< 12^o^ C max for summer growth [14]) indicating this unique calcification may already be restricted by temperature effects on algal compounds. Further research is required to test this proposal, however, climate change research on this genera should take into account this unique calcification process when considering how temperature change may impact calcification.

### Relevance for climate archiving

Understanding controls on Mg content is important because the amount of Mg incorporated into carbonate can reflect a change in mineral type and, Mg is the most commonly used element for temperature reconstruction (e.g. [18,98]). It has previously been established that hypothallial-style growth needs to be avoided when measuring Mg for climate proxy work [38,39]. That caution has been reaffirmed by the findings in this study. Our study has established the Mg bands within cell walls recently documented [8,118] are likely remnants of PCW, and that XRD patterns showing dolomite asymmetry off the Mg-calcite peak indicate the presence of D-type carbonate in these bands. Ragazzola et al., [118] showed that these bands in *Lithothamnion glaciale*, could be seasonally affected, being absent in winter growth, and were absent in higher CO_2_ treatments. We have not attempted to answer the question of why these bands may be absent, or if present why they would have lower Mg. Finding the dolomite-composition values in cell-wall banding in CCA from colder climates of Scotland, to tropical environments of Panama and Ashmore Reef (S3 Table) indicates that temperature is not the predominant control. Further an absence of measurable change in the higher Mg-calcite composition for experimental CCA *Lithophyllum cabiochae* [97] over a 3°C offset further supports a lack of temperature control. The *L*. *cabiochae* experiment also found no effect of higher CO_2_ on the higher Mg-calcite composition. Thus response of the cell-wall Mg banding to temperature and CO_2_ may be species-specific or inconsistently present.

This improved understanding of calcification in coralline algae will aid in making more accurate paleo-environmental reconstructions. A key message from this study is the importance of comparing corallines with the same type of morphology, that is, temperature calibrations developed for rhodoliths with internal banding and D-type carbonate are not applicable for encrusting CCA such as *Clathromorphum*. Further, it is important to ensure that Mg measurement transects do not include switches to PCW-only cell walls as these could be interpreted falsely as a warming signal.

Our model shows that the bands generally considered to represent summer and winter (e.g. [18]), may instead be representing a switch in growth type that could be unrelated to seasonal changes and perhaps are driven by light changes [26] or other as yet unidentified drivers. Where the bands in rhodoliths have been experimentally demonstrated to form annually [18] the key question that needs to be answered before using the higher Mg for summer temperature proxies, is how much of this higher Mg is actually driven by temperature, as compared to the anatomical change. Assuming that growth rate is consistent over the year and using branch axial distance as a proxy for time may also be problematic as the PCW-only long cells typically are faster growing than the thick-walled SCW perithallial cells. Furthermore, the rhodolith Mg-content shift represents a change to different minerals, i.e. VHMC, dolomite. Therefore, it is critically important for climate proxy work to consider whether the Mg-content reported is determined by mineral analyses, i.e. XRD, which determines the changes in Mg-content of the Mg-calcite phases and shows when other phases are present, or by bulk methods such as electron microprobe analysis, ion microprobe analysis, SEM-EDS, laser ablation, which only measure total Mg-content without identifying minerals present.

## Concluding remarks

The calcification model we have developed is consistent with cell wall formation in both higher plants and fleshy red algae and accounts for the complex array of carbonate forms and Mg-compositions present within coralline algae. Support is in favour of calcification being an induced, not a controlled process. Further work is required to determine the key organic compounds required for each type of calcification. This improved understanding will allow identification of the response of each component part to different environmental conditions, and could aid in understanding responses to climate change. Future experimental work should look to the properties of plant cell walls to guide investigations.

## Methods

### Scanning electron microscopy- Energy dispersive spectroscopy

SEM-EDS was made using a Zeiss UltraPlus field emission scanning electron microscope (SEM) equipped with HKL electron backscatter diffraction (EDS). Samples were polished, mounted on stubs with crystal bond and platinum or carbon coated. SEM using the Zeiss was carried out at the Australian National University, Centre for Advanced Microscopy. SEM-EDS was used for spot analyses to quantify the elemental composition of representative parts of the CCA crust and confirm mineral distribution within the crust. For EDS the operating voltage was 15 kV with 11 mm working distance. A range of settings were used for imaging; secondary electron (SE) showing topography and backscatter electron imaging (BSE) which shows higher magnesium areas as darker carbonate and is useful for rapid visual identification of mineral distribution. The EDS data for comparison of the cell wall and interfilament in the *Clathromorphum circumscriptum* and *C*. *compactum* was obtained using the Zeiss.

A second round of SEM and EDS was undertaken using a NOVA NanoSEM FEI at the Smithsonian Institution National Museum of Natural History’s Department of Mineralogy with the aim of measuring the Mg content of 200–300 nm bands visible with SEM-BSE within the cell walls. A setting of 7 kV, working distance 6.0 mm and 0.34 nA current was found most effective. This setting results in a smaller interaction volume (measurement field) than the 15 kV. This lower voltage was used for the EDS line. Further information on the calibration undertaken for this EDS can be found in Nash and Adey [38].

### X-ray diffraction

The rhodolith samples were ground to a fine powder using a mortar and pestle. Fluorite was added as an internal standard to allow precise alignment of the XRD pattern. The powder sample was placed on a quartz low-background slide. Powder X-ray diffraction was carried out at the Australian National University with a SIEMENS D501 Bragg-Brentano diffractometer equipped with a graphite monochromator and scintillation detector, using CuKα radiation. Scan range was 25°- 33° 2-theta, covering the aragonite, Mg-calcite, dolomite (Ca_0.5_Mg_0.5_CO_3_) and magnesite (MgCO_3_) main peaks, step size 0.02°, 2-theta, and scan speed was 1°/min. XRD scan processing used the SIEMANS software package, Diffrac-plus EVA 10. Peak interpretation followed Nash et al., [119] to determine the mol% MgCO_3_ of the Mg-calcite and to check for and quantify peak asymmetry attributable to the presence of higher Mg-phases.

### Imaging

Imaging of the crusts for the photosynthetic pigment patterns was carried out using an Olympus (Tokyo, Japan) DSX-100 opto-digital microscope with e16x zoom optics and 4 segment polarized LED ring light in the Smithsonian Institution, National Museum of Natural History, Department of Botany Microscopy Section.

### Sample collection

Samples were supplied from existing collections or were samples used for previously published studies referenced herein, and additionally [120,121]. Samples analysed, collection details and relevant references are in S3 Table.

### Etching

CCA were polished on fine sandpaper then cleaned in a sonic bath of deionised water, pH ~6–6.5, for 20–50 minutes.

## Supporting information

S1 Table Species table. Samples examined by SEM.
(DOCX)

S2 Table EDS mol% MgCO_3_ data. (DOCX)

S3 Table Sample collection locations and mineralogy type. (DOCX)
