# Supplementary material for: Coralline algal calcification: A morphological and process-based understanding
Source: PLoS One. 2019 Sep 26;14(9):e0221396. doi: 10.1371/journal.pone.0221396 (PMC6762179; doi:10.1371/journal.pone.0221396)
Supplement: S1 Table — Samples examined by SEM. (DOCX) [file pone.0221396.s001.docx]

Supplementary information, Nash et al.

**SI Table 1- Species table.** Samples examined by SEM. NF-figure not included.

| Family | Subfamily | Genus | Species | PCW | SCW |
| --- | --- | --- | --- | --- | --- |
| Hapalidiaceae | Melobesioideae | *Kvaleya* | *epilaeve* | ✔ | ✔ |
|  |  | *Leptophytum* | *leave* | ✔ | ✔ |
|  |  | *Clathromorphum* | *circumscriptum* | ✔ | ✔ |
|  |  |  | *compactum* | ✔ | ✔ |
|  |  |  | *nereostratum* | ✔ | ✔ |
|  |  | *Phymatolithon* | *borealis* | ✔ | ✔ |
|  |  |  | *investiens* | ✔ | ✔ |
|  |  |  | *laevigatum* | ✔ | ✔ |
|  |  |  | *rugulosum* | ✔ | ✔ |
|  |  |  | *squamulosum* NF | ✔ | ✔ |
|  |  | *Lithothamnion* | *glaciale* | ✔ | ✔ |
|  |  | *Mesophyllum* | *erubescens*  NF | ✔ | ✔ |
| Corallinaceae | Lithophylloideae | *Lithophyllum* | *cabioache* | ✔ | ✔ |
|  |  |  | *kotschyanum*  NF | ✔ | ✔ |
|  |  | *Titanederma* | *bermudensis*  NF | ✔ | ✔ |
|  | Porolithoideae    Hydrolithoideae  Neogoniolithoideae | *Porolithon* | *onkodes* | ✔ | ✔ |
|  |  | *Hydrolithon* | *reinboldii*  NF | ✔ | ✔ |
|  |  | *Neogoniolithon* | *brassica-florida*  NF | ✔ | ✔ |
|  |  | *Corallina* sp |  | ✔ | ✔ |
|  | Lithophylloideae | *Amphiroa* | *fragilissima* | ✔ | ✔ |
|  | [Corallinoideae](https://species.wikimedia.org/wiki/Corallinoideae) | *Jania* | *rosea* | ✔ | ? |
| Sporolithaceae | - | *Sporolithon* | *durum*  NF | ✔ | ✔ |

.
