# Supplementary material for: Coralline algal calcification: A morphological and process-based understanding
Source: PLoS One. 2019 Sep 26;14(9):e0221396. doi: 10.1371/journal.pone.0221396 (PMC6762179; doi:10.1371/journal.pone.0221396)
Supplement: S2 Table — (DOCX) [file pone.0221396.s002.docx]

**S2 Table EDS mol% MgCO_3_ data**

**SI Table 2A. EDS Mol% MgCO_3_ for cell wall and interfilament of *Clathromorphum circumscriptum*, Norway.**

| **Spectrum** | **Species** | **Cell type** | **Mol%** |
| --- | --- | --- | --- |
| Spectrum 93 | *C. circumscriptum* | IF above meristem | 13.9 |
| Spectrum 94 | *C. circumscriptum* | IF above meristem | 12.1 |
| Spectrum 95 | *C. circumscriptum* | IF above meristem | 10.8 |
| Spectrum 96 | *C. circumscriptum* | IF above meristem | 15.7 |
| Spectrum 97 | *C. circumscriptum* | IF above meristem | 14.0 |
| Spectrum 98 | *C. circumscriptum* | IF above meristem | 12.3 |
| Spectrum 99 | *C. circumscriptum* | IF above meristem | 14.4 |
| Spectrum 100 | *C. circumscriptum* | IF above meristem | 12.0 |
|  |  | **Average** | **13.1** |
| Spectrum 101 | *C. circumscriptum* | Cell wall above meristem | 14.4 |
| Spectrum 102 | *C. circumscriptum* | Cell wall above meristem | 13.4 |
| Spectrum 103 | *C. circumscriptum* | Cell wall above meristem | 12.6 |
| Spectrum 104 | *C. circumscriptum* | Cell wall above meristem | 12.0 |
| Spectrum 105 | *C. circumscriptum* | Cell wall above meristem | 15.6 |
| Spectrum 106 | *C. circumscriptum* | Cell wall above meristem | 12.8 |
| Spectrum 107 | *C. circumscriptum* | Cell wall above meristem | 14.6 |
| Spectrum 109 | *C. circumscriptum* | Cell wall above meristem | 15.0 |
|  |  | **Average** | **13.8** |
| Significantly different (two-tailed t-test)? No | | | P=0.392 |

**SI Table 2B: EDS Mol% MgCO_3_ for cell wall and interfilament of *Clathromorphum compactum*, (Gulf of Maine, USA).**

| Spectrum | Species | Cell type | Mol% |
| --- | --- | --- | --- |
| Spectrum 9 | C. *compactum,* GOM | Interfilament mid crust | 13.6 |
| Spectrum 13 | C. *compactum*, GOM | Interfilament mid crust | 11.7 |
| Spectrum 14 | C. *compactum*, GOM | Interfilament mid crust | 14.5 |
| Spectrum 15 | C. *compactum*, GOM | Interfilament mid crust | 13.0 |
| Spectrum 16 | C. *compactum,* GOM | Interfilament mid crust | 12.6 |
| Spectrum 17 | C. *compactum,* GOM | Interfilament mid crust | 13.5 |
| Spectrum 18 | C. *compactum,* GOM | Interfilament mid crust | 10.1 |
| Spectrum 19 | C. *compactum,* GOM | Interfilament mid crust | 16.4 |

|  |  | **Average** | **13.2** |
| --- | --- | --- | --- |
| Spectrum 20 | C. *compactum,* GOM | Cell wall mid crust | 15.5 |
| Spectrum 22 | C. *compactum*, GOM | Cell wall mid crust | 18.3 |
| Spectrum 23 | C. *compactum*, GOM | Cell wall mid crust | 14.6 |
| Spectrum 24 | C. *compactum*, GOM | Cell wall mid crust | 14.4 |
| Spectrum 27 | C. *compactum,* GOM | Cell wall mid crust | 17.7 |
| Spectrum 28 | C. *compactum,* GOM | Cell wall mid crust | 14.5 |
| Spectrum 29 | C. *compactum,* GOM | Cell wall mid crust | 16.0 |
| Spectrum 31 | C. *compactum,* GOM | Cell wall mid crust | 18.0 |
|  |  | **Average** | **16.1** |
| Significantly different (two-tailed t-test)? Yes | | | **P=0.005** |

**SI Table 2C. EDS Mol% MgCO_3_ for cell wall with deltoid and without deltoid interfilament of *Clathromorphum circumscriptum*, (Greenland).**

| **Spectrum** | **Species** | **Cell type** | **Mol%** |
| --- | --- | --- | --- |
| Spectrum 126 | *C. circumscriptum* | Cell wall with deltoid | 15.6 |
| Spectrum 127 | *C. circumscriptum* | Cell wall with deltoid | 10.1 |
| Spectrum 128 | *C. circumscriptum* | Cell wall with deltoid | 12.2 |
| Spectrum 129 | *C. circumscriptum* | Cell wall with deltoid | 10.5 |
| Spectrum 130 | *C. circumscriptum* | Cell wall with deltoid | 12.5 |
| Spectrum 131 | *C. circumscriptum* | Cell wall with deltoid | 11.7 |
| Spectrum 132 | *C. circumscriptum* | Cell wall with deltoid | 9.6 |
| Spectrum 133 | *C. circumscriptum* | Cell wall with deltoid | 9.2 |
|  |  | **Average** | **11.5** |
|  |  |  |  |
| Spectrum 118 | *C. circumscriptum* | Cell wall without deltoid | 15.7 |
| Spectrum 119 | *C. circumscriptum* | Cell wall without deltoid | 19.0 |
| Spectrum 120 | *C. circumscriptum* | Cell wall without deltoid | 20.5 |
| Spectrum 121 | *C. circumscriptum* | Cell wall without deltoid | 21.4 |
| Spectrum 122 | *C. circumscriptum* | Cell wall without deltoid | 15.0 |
| Spectrum 123 | *C. circumscriptum* | Cell wall without deltoid | 16.6 |
| Spectrum 124 | *C. circumscriptum* | Cell wall without deltoid | 17.2 |
| Spectrum 125 | *C. circumscriptum* | Cell wall without deltoid | 18.2 |
|  |  | **Average** | **17.9** |
| Significantly different (two-tailed t-test)? Yes | | | **P<0.001** |

**SI Table 2D: EDS Mol% MgCO_3_ for cell wall of elongate cells at transfer line of *Clathromorphum compactum*, (Gulf of Maine, USA) (Figure 41C,D).**

| Spectrum | Species | Cell type | Mol% |
| --- | --- | --- | --- |
| Spectrum 1 | C. *compactum,* GOM | Long cells transfer line | 28.1% |
| Spectrum 2 | C. *compactum*, GOM | Long cells transfer line | 21.7% |
| Spectrum 3 | C. *compactum*, GOM | Long cells transfer line | 24.2% |
| Spectrum 6 | C. *compactum*, GOM | Long cells transfer line | 22.5% |
| Spectrum 7 | C. *compactum,* GOM | Long cells transfer line | 24.7% |
| Spectrum 8 | C. *compactum,* GOM | Long cells transfer line | 24.0% |
| Spectrum 41 | C. *compactum,* GOM | Long cells transfer line | 21.4% |
| Spectrum 42 | C. *compactum,* GOM | Long cells transfer line | 24.1% |
|  |  | **Average** | **23.7%** |
| Significantly different from cell wall beneath (two-tailed t-test)? Yes | | | **P=<0.001** |
