# Supplementary material for: Coralline algal calcification: A morphological and process-based understanding
Source: PLoS One. 2019 Sep 26;14(9):e0221396. doi: 10.1371/journal.pone.0221396 (PMC6762179; doi:10.1371/journal.pone.0221396)
Supplement: S3 Table — (DOCX) [file pone.0221396.s003.docx]

**S3 Table. Sample collection locations and mineralogy type**

**SI Table 3A: Non-geniculate coralline algae.**

| Genera | Species | Sample id. | Location collected | Date  Collected | Identified by | Reference | XRD mol% | Asymm mol% | Type | Id. method |
| --- | --- | --- | --- | --- | --- | --- | --- | --- | --- | --- |
| *Porolithon* | *onkodes* (Heydrich) Foslie | HF35 | Heron Is.  GBR  Australia | November 2013 | G. Diaz-Pulido | [10] |  |  | D | EDS, BSE |
|  |  | H45 | Heron Is. GBR | December 2010 | M. Nash | [8] |  |  | D | BSE |
|  |  | H301 | Heron Is. GBR | December 2010 |  |  |  |  | D | BSE |
|  |  | GD71 | Lizard Is.  GBR | 2009 | G. Diaz-Pulido | [29] |  |  | D | BSE |
|  |  | GD99 | Lizard Is. GBR | 2009 |  |  |  |  | D | BSE |
| *Hydrolithon* | *reinboldii*  (Weber-van Bosse & Foslie) Foslie | ZR127-1 | Reef slope Ashmore reef, Indian Ocean, Australia | September 2013 | A. Harvey |  |  |  | D | EDS |
|  |  | ZR127-2,3,4,5  (4 samples) | Reef slope Ashmore reef. |  |  |  | 19.0 | 20.8 | D | XRD |
|  |  | ZR129-1 | Reef flat Ashmore reef. |  |  |  |  |  | D | EDS |
|  |  | ZR127-2,3,4,5  (4 samples) | Reef flat Ashmore reef. |  |  |  | 19.7 | 20.9 | D | XRD |
|  |  | AK 11-1067 | Micro atoll  Ryukyu Islands  Japan | 2011 |  | [121] | 17.9 | 19.1 | D | XRD |
|  |  | AK 11-1068 |  |  |  |  | 18.1 | 18.4 | M | XRD |
|  | sp | SI 71-50-100 | Hawaii | 1971 | W. Adey |  |  |  | M | EDS |
| *Clathromorphum* | *circumscriptum*  (Strömfelt) Foslie | SI  69-13-010U | Nth Norway | 1969 | W. Adey | [40] |  |  | M | EDS |
|  | *compactum*  (Kjellman) Foslie | SI 174 | Arctic Bay, Baffin Island |  | W. Adey | [14] |  |  | M & D* | EDS |
|  |  | UT-GRI5-3 | Aasiaat Greenland | June 2015 | J. Halfar |  |  |  | M | XRD  EDS |
|  |  | UT-site 6 | Nuuk, Greenland | June 2013 |  |  |  |  | M | XRD  EDS |
|  |  | UT MN11 | Gulf of Maine, USA |  |  |  |  |  | M & D* | EDS |
| *Phymatolithon* | *leavigatum*  (Foslie) Foslie | SI 64-26-1C s175 | Humber Arm, NF  USA | July 1964 | W. Adey | [38] |  |  | M | XRD |
|  |  | SI 64-5, 10-30G, s176 | Bras D'or Lakes, NS,  USA | June 1964 |  | [122] |  |  | M | XRD |
|  |  | SI 61-43-1, s179 | Woods Hole, Nantucket Sound MA, USA | November 1961 |  | [43] |  |  | M | XRD |
|  | *rugulosum*  Adey | SI 64-46, 30-50B, s173 | Great Isle of Valen, NF, USA | August 1964 |  | [122] |  |  | M | XRD |
|  |  | SI 66-27, 30-50c, s208 | Heimey, Westmanneyar, Iceland | August 1966 |  |  |  |  | M | XRD |
|  | *borealis* | SI 66-26-4, 0-10, s136 | Grindavik, Iceland | November 1966 |  |  |  |  | M | XRD |
|  |  | SI 66-42-1, 0-10F, s 224 | Trondheim fjord, S. Norway | November 1963 |  |  |  |  | M | XRD |
|  | *investiens*  Foslie | SI FT-233 s273 | Lyngo, Tromso, N. Norway | 1890’s | M. Foslie |  |  |  | M | XRD |
|  |  | SI 69-8(2) 0, s280 | Revsbotn  Rolfroysund, N. Norway | June 1969 | W. Adey |  |  |  | M | XRD |
| *Leptophytum* | *leave* | SI 2013-11(1) | Port Manvers Bay, Labrador  *K. epilaeve* growing on *L. laeve* | July 2013 |  | [37] |  |  | M | XRD |
| *Kvaleya* | *epilaeve* |  |  |  |  |  |  |  |  |  |
| *Spongites* | | PNG DA | Milne Bay, PNG | April 2011 | A. Harvey |  | 17.9 | 20.6 | D | XRD,  SEM |
| *Lithothamnion* | sp. | LLC control | Panama |  |  | [63] |  |  | D | EDS |
|  | *glaciale* | NK SID 21 T4 | Scotland |  |  | [18] | 13.0 | 14.8 | D | XRD, EDS |
|  | *pygmaeum* | GD | Lizard Is. GBR, Australia | November 2009 | G. Diaz-Pulido |  | 18.1 | 21.0 | D | XRD, SEM |
| *Lithophyllum* | *kotschyanum* | GD |  |  |  |  | 17.3 | 19.1 | D | XRD, SEM |
|  |  | AK 07-833 | Ryukyu Islands  Japan | 2007 |  | [121] | 18.1 | 20.4 | D | XRD  EDS |
|  | *cabiochae* | 10 samples | Mediterranean |  | S. Martin | [97] |  |  | D | XRD |
|  |  | 400T(2) |  |  |  |  |  |  | D | EDS |
| *Neogoniolithon* | *brassica-florida* | AK 07-886 | Ryukyu Islands  Japan |  |  | [121] | 18.3 | 18.3 | M | XRD  EDS |
| *Sporolithon* | *durum*  (Foslie) R.A.Townsend & Woelkerling | State Herbarium of SA AD-A64581 | 6 m depth Kangaroo Is.  SA, Australia | 1995 |  |  |  |  | M | EDS |
|  |  | PNG  T1 S2 | 3-5 m Milne Bay  PNG | April 2011 | A. Harvey |  | 18.0 | 18.9 | M | EDS |
| *Tenarea* | *bermudense*  [Lithophyllum stictiforme (Areschoug) Hauck](http://www.algaebase.org/search/species/detail/?species_id=kb1ea21689f1a9778&sk=0&from=results)  is the current accepted name of this entity | SI- 72-20-7A |  | 1972 |  |  |  |  | D | EDS |
| *Mesophyllum* | *erubescens*  (Foslie) Me.Lemoine | AK 07-927 | Ryukyu Islands,  Japan | 2007 |  | [121] | 17.3 | 19.0 | D | EDS |
| *Pneophyllum* | *conicum*  (E.Y.Dawson) Keats, Y.M.Chamberlain & M.Baba | AK 07-967 |  |  |  | [121] | 17.2 | 19.2 | D | XRD |
|  |  | AK 07-836 |  |  |  |  | 17.8 | 20.7 | D | XRD |
| *Mastophora* | *rosea*  (C.Agardh) Setchell | AK 07-843 |  |  |  |  | 12.3 | 12.3 | M | XRD |

LTB- Latrobe University collection, Melbourne, Victoria. SI- Smithsonian Institution collection. GD- Collection at Griffith University, QLD. ZR- Museum of Western Australia collection. MEL- National Herbarium of Victoria. JCU- James Cook University herbarium collection. UT- samples held at University of Toronto. ANU- samples held at Australian National University. Id- analytical method used to identify M or D type. BSE- back scatter electron imaging, shows higher Mg bands as darker lines. EDS- EDS spot measurements. XRD- based on the amount of asymmetry off the higher Mg side of the Mg-calcite peak. * Type M & D – most analyses indicated M type, but elevated Mg in some hypothallial cells suggests the capacity to be D-type or an environmental control on whether or not M or D type forms.

**SI Table 3B: Geniculate species**

| Genera | species | Sample id | Location collected | Date  Collected | Identified by | Comment | XRD mol% | Asymm. Mol% | Type | Id. method |
| --- | --- | --- | --- | --- | --- | --- | --- | --- | --- | --- |
| *Corallina* | sp*.* | LTB 20635 | 13-14 m Solitary Is. Coffs Harbour, NSW, Aust. | August 1996 | A. Harvey |  |  |  | M | EDS |
|  |  | LTB 20625 | 15 m ,Korffs Islet, Coffs Harbour,  NSW, Aust. |  |  |  | 14.4 | 15.2 | M | XRD |
|  |  | LTB 20486 | 15-17 m, South West side Split Solitary Island  NSW, Aust. | June 1996 |  |  | 15.3 | 16.3 | M | XRD |
|  |  | LTB20635 |  |  |  |  | 15.9 | 16.7 | M | XRD |
| *Jania* | *rosea* (Lamarck) Decaisne | LTB 20077 | 17 m Jervis Bay, NSW, Aust. | March 1996 | A. Harvey |  | 15.0 | 15.0 | M | XRD |
|  |  | LTB 20548 | Intertidal pool, Lennox Head, NSW, Aust. | May 1997 |  |  | 15.8 | 16.3 | M | XRD |
|  |  | LTB 18204 | 1-2 m  Coffs Harbour,  NSW, Aust. | February 2013 |  |  | 16.3 | 16.8 | M | XRD |
|  |  | LTB 18193 |  |  |  |  | 15.3 | 15.8 | M | XRD |
|  |  | LTB 18212 |  |  |  |  | 15.7 | 15.9 | M | XRD |
|  |  | LTB 18182 | 1-2 m Woolgoolga,  NSW, Aust. | February 2013 |  |  | 16.0 | 17.0 | M | XRD |
|  |  | LTB-18127 | Tathra,  NSW, Aust. | December 2013 |  |  |  |  | M | EDS |
|  |  | LTB 18272 | Intertidal Phillip Is.  Victoria, Aust. | October 2014 |  |  | 13.8 | 14.4 | M | XRD |
|  |  | LTB 18161 | Rockpool  Phillip Is.  Victoria, Aust. | December 2012 |  |  | 14.3 | 14.8 | M | XRD |
|  | *pedunclata* J.V.Lamouroux | LTB 18347 | 0-2 m, Jervis Bay, NSW, Aust. | March 1996 |  |  | 14.3 | 14.3 | M | XRD |
|  |  | LTB 18183 | 1-2 m Woolgoolga,  NSW, Aust. | February 2013 |  |  | 15.4 | 15.7 | M | XRD |
|  |  | LTB 18198 | 1-2 m, Coffs Harbour  NSW, Aust. | February 2012 |  |  | 15.3 | 15.6 | M | XRD |
|  | *crassa* J.V.Lamouroux | LTB 18190 | 1-2 m, Coffs Harbour NSW, Aust. | February 2013 |  |  | 16.1 | 17 | M | XRD |
|  |  | LTB 18218 |  |  |  |  | 15.6 | 15.7 | M | XRD |
|  |  | LTB 18178 | 1-2 m, Woolgoolga,  NSW, Aust. | February  2013 |  |  | 16.5 | 16.8 | M | XRD |
|  | *sagittata* (J.V.Lamouroux) Blainville | LTB 18215 | 1-2 m, Pebbly Beach,  NSW, Aust. | February  2013 |  |  | 17.1 | 18 | M | XRD |
|  |  | LTB 18189 | 1-2 m, Woolgoolga  NSW, Aust. | February 2013 |  |  | 16.1 | 17.4 | M | XRD |
|  |  | LTB 18197 | 1-2 m, Coffs Harbour  NSW,  Aust. | February 2013 |  |  | 16.9 | 17.9 | M | XRD |
|  |  | LTB 18165 | Rockpool Phillip Is.  Victoria, Aust. | December 2012 |  |  | 14.8 | 15.8 | M | XRD |
|  |  | LTB 18252 | 2-3 m Phillip Is.  Victoria  Aust. | January 2013 |  |  | 15.1 | 16.5 | M | XRD |
| *Lithothrix* | *aspergillum* J.E.Gray | LTB 16545 | 3-5 m, Santa Barbara  USA | June 1999 |  |  | 13.8 | 14.4 | M | XRD |
| *Amphiroa* | sp. | LTB 20547 | Lennoxhead  NSW  Rockpools | May 1997 | A. Harvey |  | 18.1 | 21 | D | XRD |
|  |  | ANUMN49 | 3-5 m, Sunshine coast  Queensland, Aust. | March 2015 | B. Lewis |  | 18.0 | 20.7 | D | XRD |
|  |  | ANUMN50 |  |  |  |  | 18.4 | 20.7 | D | XRD |
| *Amphiroa* | *fragilissima* (Linnaeus) J.V.Lamouroux | ANUMN 53 | 6 m, Kirra Beach  Queensland, Aust. | May 2014 | A. Harvey |  | 18.4 | 21.2 | D | XRD |
|  |  | JCU S-6346.1 | Seawards of lagoon, Mauritius | 1984 | I. Price |  | 18.3 | 20.4 | D | XRD |
|  | *foliacea* J.V.Lamouroux | JCU A-4858.1 | Lower intertidal, Badu Is.  Torres Strait | October 1979 |  |  | 18.3 | 20.2 | D | XRD |
|  | *anceps* Lamarck) Decaisne | Mel 504227 | Shaded rockpool South Gippsland,  Waratah Bay,  Victoria, Aust. | November 1973 | H. Womersley |  | 15.3 | 17.8 | D | XRD |
|  |  | LTB 18327 | 3-4 m Warrnambool  Victoria,  Aust. | August 1999 |  |  |  |  | D | EDS |
| *Amphiroa* | *beauvoisii* J.V.Lamouroux | LTB 20958 | 5-10 m, Tweed heads,  NSW, Aust. | October 1997 | A. Harvey |  | 16.9 | 19.7 | D | XRD |
|  |  | LTB 20980 |  |  |  |  | 17.1 | 19.8 | D | XRD |
|  |  | LTB 20085 | 0-2 m, Jervis bay,  NSW/ACT, Aust. | March 1996 |  |  | 17.6 | 20.1 | D | XRD |
|  |  | LTB 20078 | 17-20 m, Jervis bay,  NSW/ACT, Aust. |  |  |  | 17.9 | 19.9 | D | XRD |
|  |  | LTB 20111 | 2-3 m, Jervis bay,  NSW/ACT, Aust. |  |  | Shorter branches | 15.9 | 17.0 | M? | XRD |
|  |  | LTB 20522 | 11m, Byron Bay,  NSW,  Aust. | April 1997 |  |  | 18.3 | 20.5 | D | XRD |
|  |  | LTB 20561 | 0-1 m, Lennox Head,  NSW, Aust. |  |  |  | 17.8 | 21.1 | D | XRD |
|  |  | LTB  20624 | 15 m,  Coffs Harbour,  NSW, Aust. | August 1996 |  |  | 17.8 | 19.7 | D | XRD |
| *Amphiroa* | *klochkovana* A.S.Harvey, W.J.Woelkerling & A.J.K.Millar | LTB 20485 | 15-17 m, Solitary Is.  NSW, Aust. | June 1996 | Holotype  A. Harvey | Flat branches | 19.2 | 21.7 | D | XRD |
|  |  |  |  |  |  | Round branches | 17.6 | 21.1 | D | XRD |
|  |  | LTB 20637 |  | August 1996 | A. Harvey |  | 18.8 | 21.4 | D | XRD |
| *Mesophyllum* | *engelharti* (Foslie) W.H.Adey | LTB 17974 | 0-2 m, Phillip Is  Victoria, Aust. | January 2000 |  |  |  |  | M | XRD |
| *Metogoniolithon* | *stelliferum* (Lamarck) Ducker | ANU SI 082017 | Phillip Is.  Victoria, Aust. | September 2015 |  |  | 15.0 | 15.0 | M | XRD  EDS |
